# COSMOSol: efficient solvent screening for polymer additives with open-source COSMO-SAC

**DOI:** 10.1039/d6ra05129d

**Published:** 2026-07-21

**Authors:** Adam Bouz, Juraj Kosek, Martin Klajmon

**Affiliations:** a Faculty of Chemical Engineering, University of Chemistry and Technology, Prague Technická 5 166 28 Prague 6 Czechia adam.bouz@vscht.cz

## Abstract

Recycling polymers with solvents requires removing additives efficiently—yet for most of the hundreds of additives used industrially, the efficiency of various solvents to dissolve them is unknown, particularly due to the scarcity of experimental solubility data. This work proposes a systematic computational approach using the quantum mechanics-aided open-source COSMO-SAC model to screen solvents and requires only the fusion data of the additive as external input, or even no empirical input. To validate the method, we compiled solubility measurements for five representative additives across diverse solvent classes. Quantitatively, predictions typically fall within 0.5 log units of measured values (in mole fraction) and, more importantly, qualitatively correctly identify both the best and worst solvent types for each additive. Furthermore, we examined how different modeling choices affect predictions: for instance, values of fusion properties, molecular conformation and σ-profile of the additive, and dispersive interactions. When fusion data are unavailable, the infinite-dilution activity coefficient provides rankings comparable to those obtained from calculated equilibrium solubilities, particularly for poorly soluble additives. Minor limitations of the approach were observed in cases where solvents are structurally very similar—here, fine distinctions between them can be affected by interference from other effects or model simplifications. However, for choosing between different solvent classes—the decision that matters most in practice—the rankings prove robust. We additionally benchmarked COSMO-SAC against HANNA, a machine-learning activity-coefficient model: the two yield comparable solvent rankings. To facilitate reproducibility and extension of this work, we provide COSMOSol, a Python toolset containing all workflows and data. The method offers a practical, first-principles route to solvent screening—not only for polymer additives, but potentially for other compound classes where experimental data are lacking.

## Abstract 2

Open-source COSMO-SAC enables first-principles-aided solvent screening of polymer additives. COSMOSol, our Python tool, makes it accessible—not only for additives, but for other compound classes where experimental data are lacking.

## Graphical abstract

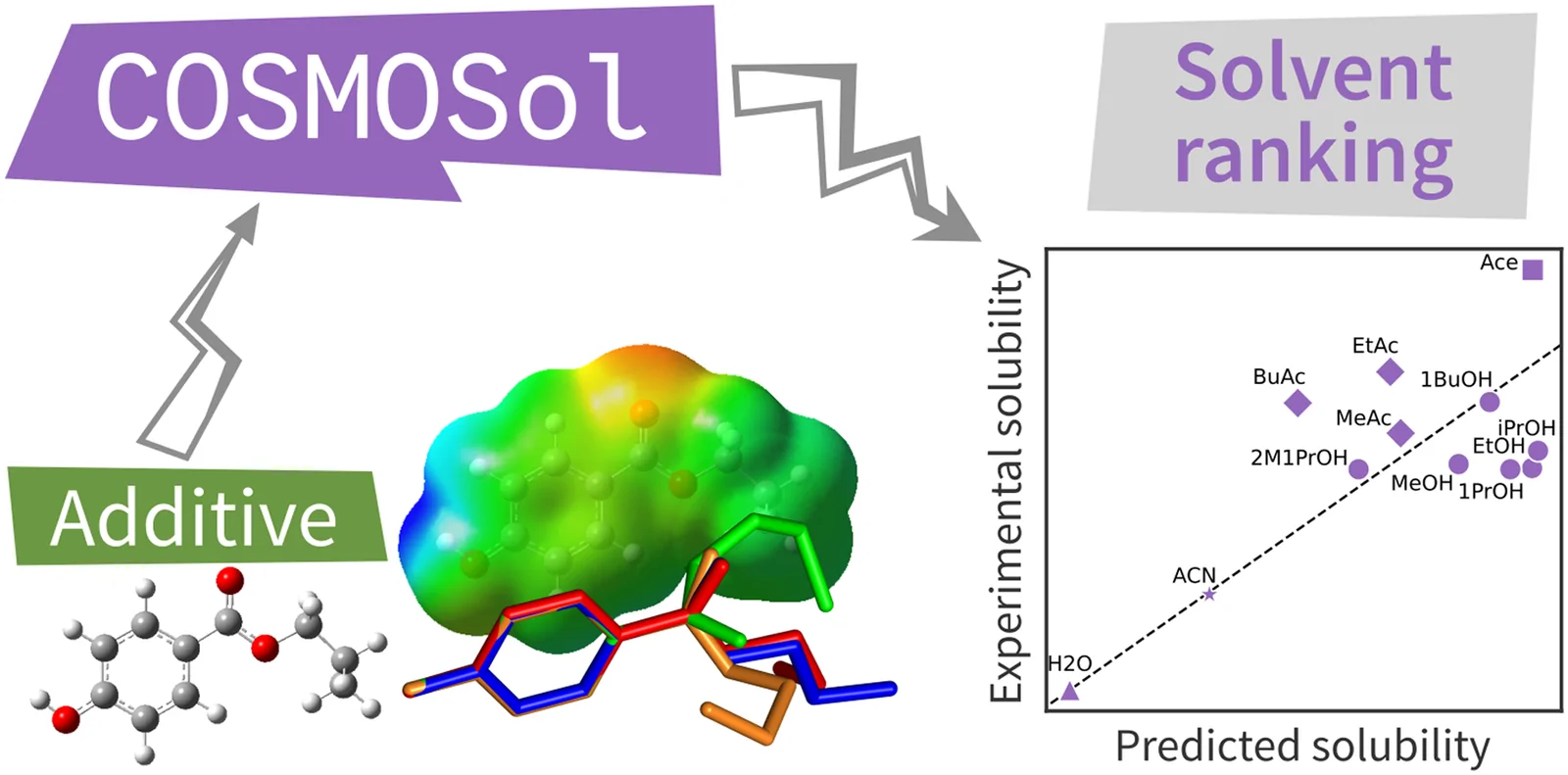

## 1 Introduction

The development of polymer recycling technologies for various plastic waste streams plays a crucial role in advancing the circular economy of plastics. In general, plastic recycling technologies can be categorized into four main classes (ranked from the least to the most energy-intensive): (i) mechanical recycling in an extruder, (ii) solvent-based purification methods, (iii) pyrolysis, solvolysis and chemical, catalytic or enzymatic depolymerization, and (iv) incineration recovery of the energy content.††Incineration is traditionally categorized as recycling in some engineering hierarchies (*e.g.*, ASTM D5033), but current EU frameworks strictly distinguish between recycling (material recovery) and energy recovery. Recent reports^1^ highlight the need for refined definitions and clear guidelines for calculation rules and quality of recyclate. Solvent-based recycling^2,3^ is an up-cycling strategy based on a simple dissolution of the target polymer in appropriate solvents. Most synthetic polymers contain a range of additives (typically about 7 weight% of the polymer^4^) that are dispersed in the polymer material in the form of molecules or small particles. Moreover, a significant fraction of the waste is present in the form of composites, which are immiscible mixtures of polymers and/or other materials. Solvent-recycling aims to recover high-value recyclate by selectively dissolving a specific polymer (*e.g.*, polyethylene and polystyrene). The subsequent extraction steps aim to extract unwanted additives, after which the purified polymer is precipitated back into its solid form. The rationale of such solvent-based technologies is to yield a high-quality, virgin-like resin free from plastic contaminants and intentionally or non-intentionally added substances.^3^ However, the effective removal of these additives represents a major bottleneck for closed-loop recycling.^5^ To illustrate the sheer scale and complexity of this challenge, Ügdüler *et al.* compiled an extensive review of various solvent-based extraction methods and their target additives (see Table 3.1 in ref. 5).

Solvent-based recycling methods will only be effective with the optimal selection of solvents. Although the solubility behavior of common commodity polymers themselves is reasonably well understood experimentally,‡‡Although predicting the solubility of polymers computationally is still a much underexplored field, recent efforts are rapidly advancing this direction. Notably, Zhou *et al*.^6^ established a freely accessible database providing solubility predictions at both room and elevated temperatures for more than 1000 solvents in various key polymers, including EVOH, PE, PP, PS, PET, PVC, and nylon, thereby effectively substituting conventional trial-and-error screening.^3^ Furthermore, Walker *et al.*^7^ successfully integrated COSMO-RS into their solvent screening workflow to guide the thermodynamic design of the Solvent-Targeted Recovery and Precipitation (STRAP) process for recycling multilayer plastic films. While extending our methodology to polymer systems falls outside the scope of the present work, the application of COSMO-based models is clearly advancing in this direction.^8–10^ the main challenge lies in the scarcity of high-quality solubility data for additives. This is largely due to the fact that there are too many additives that serve various functions in polymers (summarized in Table 1). Additives are typically diverse organic molecules and each class of them may include dozens to hundreds of representatives. An additional complication is that the input waste stream rarely contains a polymer with a well-defined additive composition—this composition varies by manufacturer and application area and is often confidential. Consequently, due to the broad variety of chemical properties among both additives and plastics, identifying an effective extraction solvent experimentally relies heavily on resource-intensive trial-and-error approaches.^5^ Fortunately, between 2016 and 2018, a large-scale screening^11^ of most commonly produced additives was carried out in the EU through a collaboration between the European Chemicals Agency and European polymer manufacturers. The outcome of the mapping is a list^12^ of more than 400 additives and pigments registered under REACH regulation in quantities exceeding 100 tonnes per year.

**Table 1:** Overview on the functions of plastic additives (OECD 2019).^13^ Note that each sub-class involves a large body of individual chemicals

Additive function	Sub-class
Additives for processability	Plasticizers
	Lubricants
	Blowing agents
Surface protector/modifier	Antistatic agents
	Antifriction agents
	Adhesion-improving agents
	Anti-fog additives
Material protectants	Antioxidants
	Light stabilizers
	Ultraviolet-absorbing agents
	Thermo-stabilizers
Physical–chemical property improvers	Flame retardants
	Fillers/reinforcement materials
	Compatibilizers
Functionalization agents	Coloring agents

For identifying optimal solvent media for additives that combine effectiveness with an acceptable environmental profile, laborious and costly experiments can, to a large extent, be replaced by computational methods of varying sophistication, such as solubility-parameter approaches,^14^ group-contribution (GC) methods,^15,16^ machine-learning (ML) approaches,^17–20^ and COSMO-based methods.^21–25^ Each of these classes has identified strengths and limitations. For example, traditional GC methods may suffer from incomplete interaction-parameter matrices,^26^ whereas ML approaches may struggle with thermodynamic consistency, particularly when not embedded within an established thermodynamic framework^27^ (however, recent works have begun to address these issues^17,28^). For these and other reasons, ML models are increasingly viewed “as a refinement of physics-based models, rather than as a universal replacement”, as recently aptly concluded by Cysewski *et al.*^29^ Consequently, baseline physically sound theories and their predictive performance remain of central importance.

COSMO-based models, such as the original COSMO-RS^21,22^ and COSMO-SAC,^23,24^ have gained significant attention over the past decades, primarily due to their physical background, relatively straightforward application (provided suitable software is accessible) and their notable performance. These mesoscale models combine quantum-mechanical (QM) determination of molecular properties with statistical thermodynamic processing, employing a surface-interaction model to predict macroscopic solution properties such as the heat of mixing, activity coefficients, and phase equilibria, including solid solubility.^30^ This enables fast, high-throughput, and first-principles-based solvent screening with the molecular structure as the only input and, unlike ML methods, without the need for training on experimental data (certain phase equilibrium types require additional input data, such as fusion properties in case of solid solubility, as detailed in Section 2.1).

The potential of COSMO-based models for qualitative screening of solvents for organic solids has already been examined multiple times, for instance, in the context of drugs,^9,31–35^ pesticides,^32^ polyaromatic hydrocarbons,^31,36^ and biopolymers.^37,38^ In addition, applications are also available, for example for vapor–liquid and liquid–liquid equilibrium, solvation free energies, and related properties.^25,39–42^ Although the quantitative accuracy of the results may significantly vary, the conclusions regarding qualitative performance—which is crucial for solvent ranking—are consistently positive. Highly relevant in the specific context of plastic recycling is the recent work of Van Melkebeke *et al.*,^43^ who successfully employed COSMO-RS to predict the relative affinities of brominated flame retardants to various solvents. These qualitative predictions enabled the rational design of an effective dissolution recycling process for waste polystyrene, directly replacing inefficient trial-and-error experimentation.

However, despite such targeted applications, the application and systematic evaluation of COSMO-type models for a diverse set of polymer additives (antioxidants and/or pigments) is still lacking. Therefore, the goal of this study is to systematically and thoroughly explore and illustrate the capabilities of the open-source COSMO-SAC model^24^ for solvent screening for polymer additives, while also providing Python-based tools that allow other researchers not only to reproduce the calculations performed in this work but also to extend them to other additives, solvents, and conditions, making it useful for open-source, theory-based solutions to practical screening problems.

**Fig. 1:**
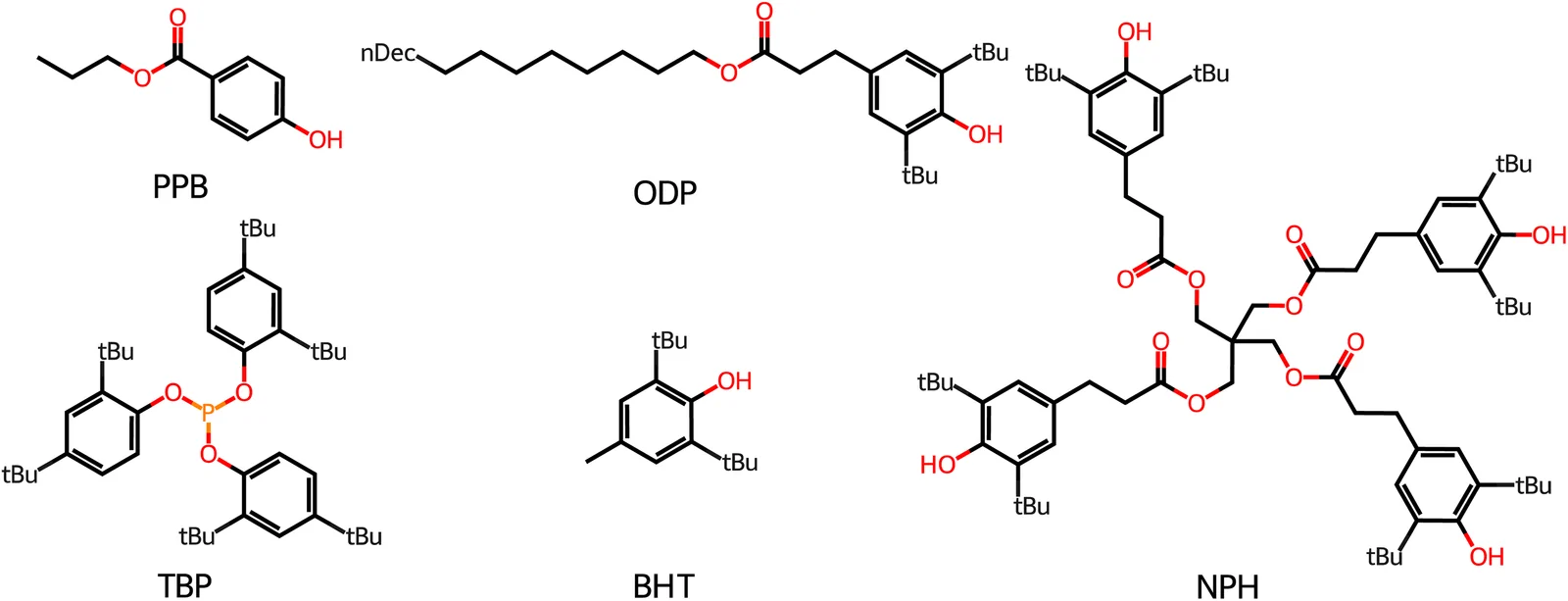
Chemical structures of the investigated additives (tBu = *tert*-butyl, nDec = *n*-decyl).

In this study, we considered five diverse polymer additives: BHT, ODP, TBP, NPH, and PPB. Their full names, chemical identifiers, and structures are provided in Table 2 and Fig. 1, respectively. This selection includes additives currently investigated in our projects, as well as those for which experimental solubility data—used as reference for evaluating COSMO-SAC's performance—are available in the literature. To contextualize the performance of COSMO-SAC relative to contemporary approaches, we compared our results with those obtained using HANNA, a state-of-the-art ML-derived activity-coefficient method.^17^

**Table 2:** Summary of the considered additives and their identifiers^a^

Alias	Name	Industrial standard^b^	Additive class	CAS RN
BHT	2,6-Di-*tert*-butyl-4-methylphenol	BHT	Primary antioxidant	128-37-0
ODP	Octadecyl 3-(3,5-di-*tert*-butyl-4-hydroxyphenyl)propionate	Antioxidant 1076	Primary antioxidant	2082-79-3
TBP	Tris(2,4-di-*tert*-butylphenyl)phosphite	Phosphite 168	Secondary antioxidant	31570-04-4
NPH	Pentaerythritol tetrakis(3,5-di-*tert*-butyl-4-hydroxyhydrocinnamate)	Antioxidant 1010	Primary antioxidant	6683-19-8
PPB	Propylparaben	E216/Nipasol	Antimicrobial agent	94-13-3

a SMILES codes are: BHT: Cc1cc(c(O)c(c1)C(C)(C)C)C(C)(C)C, ODP: CCCCCCCCCCCCCCCCCCOC(=O)CCc1cc(c(O)c(c1)C(C)(C)C)C(C)(C)C, TBP: CC(C)(C)c1ccc(OP(Oc2ccc(cc2C(C)(C)C)C(C)(C)C)Oc3ccc(cc3C(C)(C)C)C(C)(C)C)c(c1)C(C)(C)C, NPH: CC(C)(C)c1cc(cc(c1O)C(C)(C)C)CCC(=O)OCC(COC(=O)CCc2cc(c(O)c(c2)C(C)(C)C)C(C)(C)C)(COC(=O)CCc3cc(c(O)c(c3)C(C)(C)C)C(C)(C)C)COC(=O)CCc4cc(c(O)c(c4)C(C)(C)C)C(C)(C)C, PPB: O=C(OCCC)c1ccc(O)cc1.

b ODP, TBP, and NPH are also known under their commercial names Irganox 1076, Irgafos 168, and Irganox 1010, respectively.

## 2 Computational approach

### 2.1 Thermodynamic descriptors

The solvent screening problem is a combinatorically complex task. To systematically classify solvents, one or more descriptors that quantify the solute–solvent affinity is needed. Macroscopic descriptors directly related to solution thermodynamics are often used for screening purposes, such as the heat of mixing,^44^ activity coefficients,^31,37,38^ solubility parameters,^14^ or solubility itself^9,45^ (when calculable; see text below). For applications where a direct and explicit connection to thermodynamics is not required or not applicable, various other, mainly molecular-level descriptors are employed in the spirit of quantitative structure–property relationships (QSPR), such as dipole moments, solvatochromic parameters, and topological, geometric, or QM descriptors.^46,47^ In this section, the selection of descriptors used in this work is presented and discussed.

All additives considered in this study are crystalline solids at ambient temperature (see Table 2). Therefore, their solubility in solvents (either experimental or predicted) appears to be the most straightforward descriptor of the additive–solvent affinity. The solubility of a pure crystalline solid in a liquid solvent is determined by the phase equilibrium between the solid phase (*i.e.*, pure crystalline additive) and the liquid phase (a saturated solvent–additive solution). In this article, the term solubility refers to the mole fraction of the solute (*i.e.*, the additive) in the saturated solution, *x*^SLE^_a_. This solid–liquid equilibrium (SLE) is mathematically described by eqn (1). To derive this SLE equation, the chemical potentials of the solute in the solid and liquid phases are set equal and expressed relatively to the pure-liquid standard state.^48^1
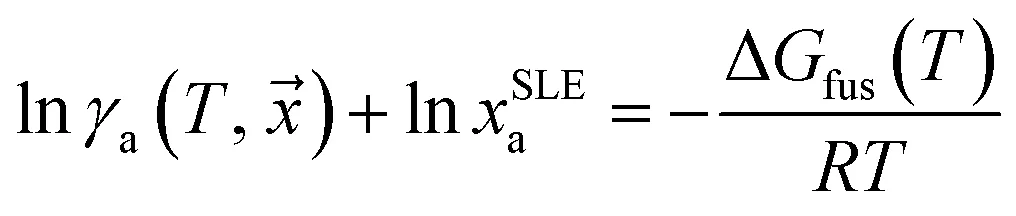


The left hand side of eqn (1) represents the (logarithmic) activity of the solute in the saturated solution (ln *a*_a_), which is further divided into two terms: ln *x*^SLE^_a_ (*i.e.*, the unknown solubility) and ln *γ*_a_, where *γ*_a_ is the activity coefficient of the solute at given temperature (*T*) and composition (*x⃑*). In the context of this study, *γ*_a_ is the key quantity, as it—for a given additive—captures the solvent effect on the solubility and, hence, additive–solvent affinity. Being the (reduced) partial molar Gibbs energy of the solute, *γ*_a_ includes both enthalpic and entropic aspects (*G* = *H* − *TS*). We use the COSMO-SAC model to predict *γ*_a_ (see Section 2.2). At the same time, *γ*_a_ is the only quantity in eqn (1) that is predicted by COSMO-SAC.

The right-hand side of eqn (1) contains the Gibbs energy of fusion, Δ*G*_fus_(*T*), of the pure additive at temperature at which solubility is evaluated. Δ*G*_fus_ depends solely on the properties of the pure additive. Δ*G*_fus_(*T*) can be expressed by thermodynamic integration of the enthalpy and entropy of fusion (Δ*H*_fus_ and Δ*S*_fus_, respectively) from the melting temperature (*T*_m_) to the temperature of interest *T* (*T* < *T*_m_). This leads to the following relation:2
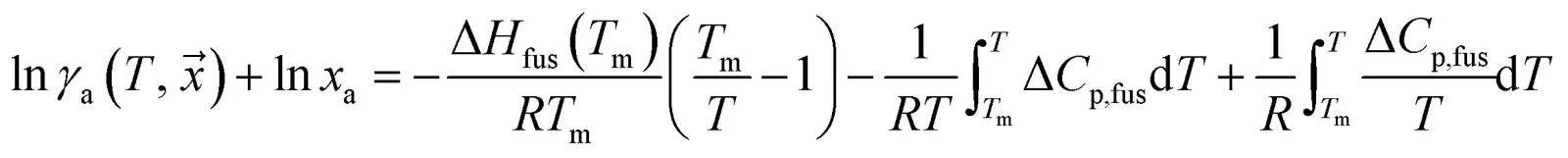
In eqn (2), Δ*C*_p,fus_ is the difference in molar isobaric heat capacity between the pure (subcooled) liquid and crystalline solid states of the solute:^48^ Δ*C*_p,fus_ = *C*^liq^_p_ − *C*^cry^_p_.

Solution of eqn (2) requires the knowledge of *T*_m_, Δ*H*_fus_(*T*_m_), and Δ*C*_p,fus_(*T*). Because *C*_p_ for the liquid phase of a high-melting solid is challenging to measure and, therefore, often unavailable, several simplifying approximations regarding Δ*C*_p,fus_ are commonly made:

(1) Treating Δ*C*_p,fus_ as temperature-independent and taking its value at *T*_m_.

(2) Using the relation Δ*S*_fus_(*T*_m_) ≈ Δ*C*_p,fus_(*T*_m_), justified by the significant gain in conformational flexibility and rotational freedom upon melting for non-rigid molecules;^49^

(3) Assuming Δ*C*_p,fus_(*T*) = 0, justified by the near-cancellation of both heat-capacity terms in eqn (2) when *T* is close to *T*_m_.^48^

The latter option, Δ*C*_p,fus_(*T*) = 0, simplifies eqn (2) to the following form:3
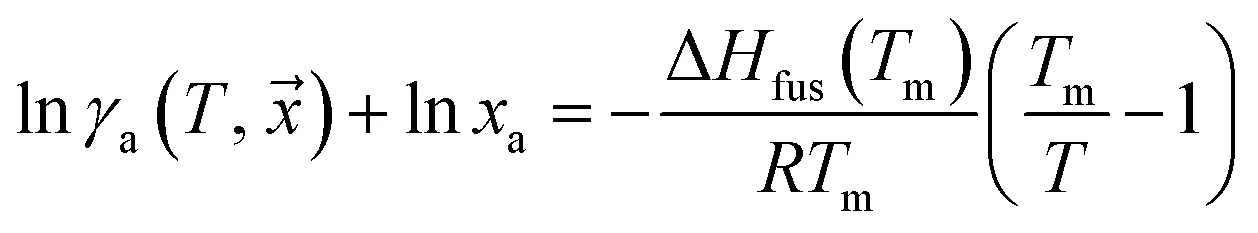


In general, the most rigorous approach is represented by the full eqn (2) or approximation (1) above, provided that *C*^liq^_p_ and *C*^cry^_p_ data are available. Approximations (2) and (3) require only Δ*H*_fus_(*T*_m_) and *T*_m_, but a universal recommendation between them is difficult and solute-dependent. However, it should be emphasized that the applied strategy regarding Δ*C*_p,fus_ is usually not governed by theoretical but rather practical considerations related to data availability. This also applies to the present work—since Δ*C*_p,fus_ was not available for any of the considered additives, we could only choose between two approximations: either neglecting the heat capacity term entirely (Δ*C*_p,fus_ = 0) or estimating it from the entropy of fusion (Δ*C*_p,fus_ ≈ Δ*S*_fus_).

Unlike Δ*C*_p,fus_ and similarly to ln *γ*_a_, the fusion temperature and enthalpy are essential components of the solubility equation (eqn (3)) and cannot be neglected. In principle, three qualitatively different treatments of *T*_m_ and Δ*H*_fus_ can be distinguished. First, the so-called “reference” solubility approach has been proposed, which uses experimental solubility data in selected reference solvents to calibrate Δ*G*_fus_ together with solvent-specific chemical potential corrections.^34^ Since this approach requires experimental solubility data to already be available, we do not consider it further. Second, *T*_m_ and Δ*H*_fus_ can be predicted computationally; however, this is generally a very challenging task. Over the past decade, methods based on molecular simulations, GC, QSPR, and ML have been proposed and tested,^50–54^ showing varying degrees of accuracy. One of the main challenges associated with these properties is polymorphism—*i.e.*, the fact that a single compound with a single SMILES code may adopt different crystal structures, each characterized by different *T*_m_, Δ*H*_fus_, and, consequently, solubility. The prediction of fusion properties is therefore tightly connected with another challenge—crystal structure prediction.^55,56^ In this context, it is somewhat unclear whether and how, *e.g.*, current GC and ML approaches treat polymorphism when designed as SMILES → property tools. Therefore, as the third option, the most common approach is to use experimentally determined *T*_m_ and Δ*H*_fus_ (typically obtained *via* differential scanning calorimetry, DSC), and to apply a computational model only to describe the non-ideal behavior of the liquid phase (ln *γ*_a_). This approach is adopted in the present work.

Experimental Δ*H*_fus_ and *T*_m_ values used for solubility calculations (eqn (3)) in this work are shown in Table 3. In our calculations, we adopt Δ*C*_p,fus_ = 0 as the primary approach, while the alternative approximation Δ*C*_p,fus_ ≈ Δ*S*_fus_ (calculated as 
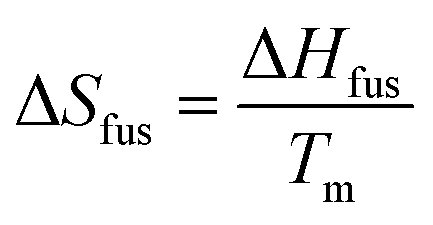
) is evaluated separately as a sensitivity test presented in Section 3.2.3.

**Table 3:** Thermodynamic fusion properties of the considered additives

Additive	Form^a^	*T*_m_/K	Δ*H*_fus_/(kJ mol^−1^)	Δ*S*_fus_/(J K^−1^ mol^−1^)^b^	Source
BHT	c	342.9	19.8	58	57
ODP	III^d^	320.65	75.1	234	58
TBP	c	458.46	43.6	95	59
NPH	*α*	385.75	66.0	171	60
PPB	c	369.5	27.9	76	61

a Specific crystalline/polymorph form considered.

b Evaluated as 
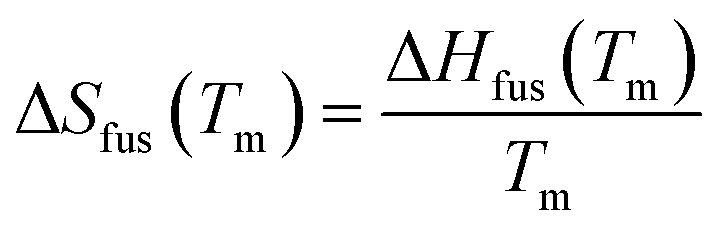
 and used instead of Δ*C*_p,fus_ in the solubility eqn (2) only within a special scenario examined in Section 3.2.3.

c Only a single crystalline form is likely known in the case of BHT, TBP, and PPB,^57,59,61–69^ characterized by the fusion properties reported, *e.g.*, in ref. 57, 59 and 61, respectively.

d Since the source of the experimental solubility data^70^ does not specify the solid form, the low-*T* stable form III was considered.^58^

In some cases, availability of the fusion properties is problematic—an issue that may be encountered with complex organic molecules among polymer additives that thermally decompose before melting, or in theoretical studies aiming to improve solubility *via* functional group modification, where the physical properties of the proposed derivatives are unknown. When dealing with additives whose melting points are not available, the SLE eqn (3) cannot be solved. In these cases, an alternative descriptor, that provides at least a qualitative estimate of solute–solvent affinity, can be introduced. A commonly used choice^38,71,72^ is the activity coefficient at infinite dilution (IDAC), ln *γ*^∞^_a_:4
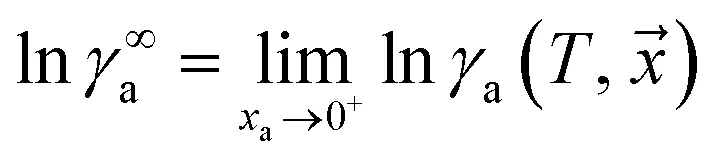


To clarify the choice of activity coefficient as a solubility descriptor, we note from eqn (1) that the solvent influences only the activity of the additive in the liquid phase, while the right-hand side remains constant at a given *T*. The challenge arises from the fact that ln *γ*_a_ is a function of molar fraction, requiring a choice of composition at which ln *γ*_a_ is evaluated. Infinite dilution is chosen because it (i) represents the state where a solute molecule is completely surrounded by solvent molecules, thus neglecting solute–solute interactions, and (ii) does not require the modeler to introduce an arbitrary finite value of *x*_a_. ln *γ*^∞^_a_ thus appears to be most valid for poorly soluble solutes, where the solubility is roughly inversely proportional to ln *γ*^∞^_a_:^38^
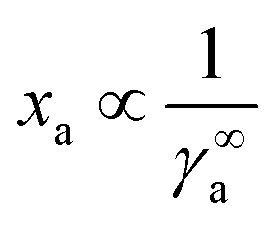
 (see eqn (1)). Therefore, lower ln *γ*^∞^_a_ implies higher solubility, and *vice versa*. Additionally, since activity coefficients are non-linear functions of composition, infinite dilution provides an extreme value that promotes the differences between different solvents, while at *x*_*i*_ → 1, ln *γ*_*i*_ → 0.

Another widely used descriptor is the excess enthalpy, *H*^E^ (also known as the heat of mixing), which quantifies the enthalpic favorability of mixing a solute and a solvent, while ignoring the entropic aspects (unlike *γ*_a_). Negative values of *H*^E^ indicate exothermic mixing, suggesting favorable interactions between solute and solvent molecules, while positive values indicate endothermic mixing, suggesting unfavorable interactions. The excess enthalpy can be computed from the temperature derivative of the excess Gibbs energy (eqn (5)), which in turn can be derived from the activity coefficients using eqn (6).5
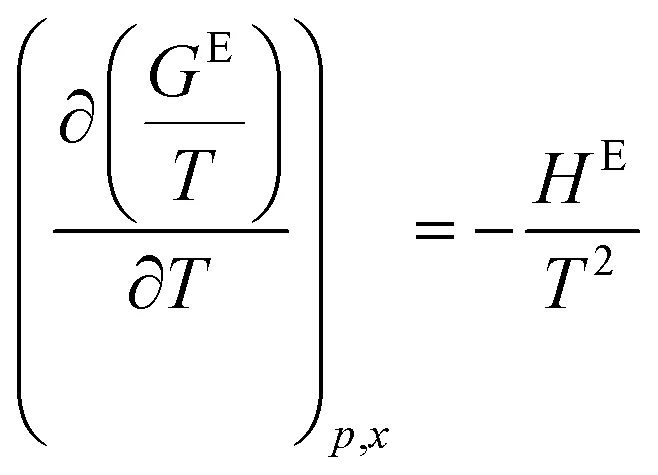
6
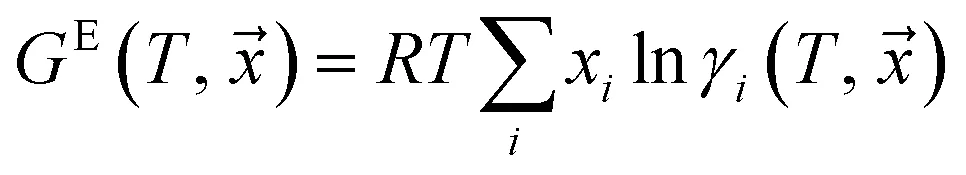
In this work, solubility, ln *γ*_a_ (both at infinite dilution and at finite concentration), and *H*^E^ are considered and examined as additive–solvent descriptors. All these quantities are predicted by the COSMO-SAC model, which is described in the following section.

### 2.2 COSMO-SAC

As mentioned earlier in this paper, COSMO-type models allow for predicting macroscopic solution properties, including *H*^E^, *G*^E^, and ln *γ*_*i*_ from QM molecular surface interactions through a statistical-thermodynamics procedure.^22,24,73^ The central concept interlinking the QM and statistical parts of these models is the *σ*-profile: a histogram of molecular surface charge density (*σ*) required for each species in the solution.

In this work, we employed the COSMO-SAC^23^ framework as implemented in the open-source package by Bell *et al.*^24^ (specifically, version 1.0.1 was used). This package provides a reference implementation of several COSMO-SAC variants. The most recent of these is the COSMO-SAC-dsp model by Hsieh *et al.*,^74^ which accounts for electrostatic misfit, hydrogen-bonding (HB), and dispersive interactions. Accordingly, the expression for the activity coefficient in this variant is expressed as the sum of three contributions and reads:

7 ln *γ*_*i*_ = ln *γ*^res^_*i*_ + ln *γ*^comb^_*i*_ + ln *γ*^dsp^_*i*_

In eqn (7), the superscript “res” refers to the residual contribution, which accounts for electrostatic interactions, “comb” denotes the combinatorial contribution, which captures molecular size and shape differences, and “dsp” is the dispersive term. The residual contribution is determined from the *σ*-profiles and molecular surface area, while the combinatorial contribution is calculated using the Staverman–Guggenheim (SG) relation.^75,76^ The SG relation requires only the molecular volume and surface area obtained from the QM calculations.

However, the dispersive interactions in COSMO-SAC-dsp are parametrized only for molecules containing C, H, N, O, F, and Cl atoms, making it somewhat limited in terms of applicability. Since our investigated molecules also contain S and P atoms, we decided to omit the dispersive correction in our reference modeling scenario (*i.e.*, ln *γ*^dsp^_*i*_ = 0). This means that we use the original COSMO-SAC-2010 variant:^77^

8 ln *γ*_*i*_ = ln *γ*^res^_*i*_ + ln *γ*^comb^_*i*_

Only in Section S3 in the SI, we demonstrate and discuss the (negligible) influence of the dispersive term (eqn (7)) on the solubility predictions for the additive–solvent systems.

Regarding HB abilities of molecules, COSMO-SAC-2010 explicitly distinguishes non-HB (NHB) part of molecular surface, HB part due to the hydroxyl group (OH), and HB due to other groups (OT). Each part is treated separately regarding model equations and parametrization. More detailed information about COSMO-SAC and its individual terms can be found in the comprehensive study by Bell *et al.*^24^

The *σ*-profiles for all solvents were taken from the UD database^78^ distributed along with the COSMO-SAC package.^24^ In contrast, none of the considered additives (except PPB) is included in UD. Therefore, the *σ*-profiles of all additives were determined in this work for consistency. First, for most additives, an initial 3D structure was generated from the SMILES code (see Table 2) using the established ETKDGv3 (ref. 79 and 80) method implemented in RDKit.^81^ This structure was subsequently optimized using RDKit at a molecular-mechanical level^82^ and finally refined quantum-mechanically at the BP86/TZVP/C-PCM level using Gaussian 16.^83^ The latter density functional theory (DFT) calculation employed the conductor-like polarizable continuum model (C-PCM)^84^ for solvation, and produced the necessary surface charge densities to calculate the *σ*-profile. Only for NPH, its structure was too complex for ETKDGv3: 193 atoms, steric crowding, *etc.* Therefore, its initial structure was not generated from SMILES but was instead adopted from a crystal structure of NPH present in the Cambridge Structural Database^63^ (CSD) (denoted “TARBOD” after the corresponding CSD refcode); all subsequent treatments remained unchanged. The obtained *σ*-profiles for all additives, together with the corresponding optimized geometries, are provided in the COSMOSol tool (see Section 2.3). The *σ*-profiles are also displayed in Fig. S1 in the SI. In principle, *σ*-profiles depend on the geometry and QM setup. Therefore, the sensitivity of the COSMO-SAC results to the treatment of *σ*-profiles in the context of additive-containing systems is discussed in the SI (Section S2).

Although Gaussian was used to obtain the *σ*-profiles for the additives considered in this work, the proposed workflow and COSMOSol do not rely exclusively on Gaussian. The modeler is free to use alternative QM programs, including freely available ones such as ORCA^85^ or GAMESS.^86^ The only requirement is consistency with respect to the QM code and level: all compounds within additive or solvent sets to be compared should be obtained using the same protocol,^24^ and this protocol should correspond to the QM level commonly employed within COSMO-SAC, *i.e.*, DFT/BP86/TZVP/(COSMO or C-PCM). Therefore, for illustration and comparison purposes, we also included an ORCA-derived *σ*-profile in the sensitivity analysis presented in Section S2. To enable this, the to_sigma.py tool integrated within open-source COSMO-SAC^24^ was extended to allow parsing of ORCA C-PCM output files with segment charges (this update is available at https://github.com/usnistgov/COSMOSAC).

### 2.3 COSMOSol: additive solubility tool

To ensure transparency and reproducibility of our solubility calculations using the open-source COSMO-SAC model,^24^ we provide a Python-based tool COSMOSol that automates the evaluation of various solubility descriptors and thermodynamic properties. The code is organized into three main modules:

• Activity_model – defines the ActivityModel interface and its backends: the open-source COSMO-SAC model^24^ and, for benchmarking, the machine-learning model HANNA.^17^ The interface allows for flexible integration of additional activity coefficient models in the future.

• Thermodynamics – implements general thermodynamic quantities and phase-equilibria solvers, independent of the underlying activity-coefficient model.

• Solubility_descriptors – facilitates convenient evaluation of multiple solubility descriptors across a set of solvents, leveraging the pandas library for data handling.

We also include all .sigma files and optimized geometries (in XYZ format) of the investigated additives, and a simple Python script for molecular geometry generation using RDKit. Template Gaussian input file for the QM calculations can be found in the COSMOPharm project distributed on GitHub.^9,87^ The entire toolset is openly available for academic and research use at https://github.com/AdamBouz/COSMOSol.

### 2.4 Reference solid solubility data

In order to evaluate both quantitative and qualitative predictive capabilities of a model, a comparison with reference experimental results is essential. However, reliable and well-reported experimental data on solubility of (solid) polymer additives in ordinary solvents are quite rare. Nevertheless, we found relevant sources in the form of standard research papers in case of BHT (ref. 57, 64 and 88), TBP (ref. 59), NPH (ref. 60 and 89) and PPB (ref. 61). These works were found reliable, since they represent primary experimental sources and explicitly report methodology, Δ*H*_fus_, *T*_m_, purity of the chemicals, and resulting solubility values in unambiguous units.

However, given the general scarcity of solubility data for common polymer additives, we additionally utilized data reported in technical datasheets of commercial suppliers in case of ODP (ref. 70), TBP (ref. 90) and NPH (ref. 91). This type of source typically lacks the important experimental details outlined above (method, Δ*H*_fus_, *T*_m_, *etc.*). Furthermore, the solubility values are presented with only two significant digits and appear to be considerably rounded. Despite not meeting established standards for reporting experimental solubility data, assuming that the reported values were obtained using the same solid material and the same (albeit unreported) solubility method, they can still be taken into account to provide a rough, qualitative ranking of solvent efficiency and to extend the considered dataset. The list of all solvents considered in this work, including their abbreviations and identifiers, is shown in Table S1 in the SI. Once the experimental sources were identified, careful curation followed due to challenges like polymorphism in the solid state (see Table 3), inconsistent solubility units and temperature ranges. As part of this procedure, for more convenient and flexible handling of experimental solubility data, we did not directly use primary values but rather their correlations *via* the modified Apelblat equation^92^ (eqn (9)), either provided in the original sources or developed independently within this work. This approach allowed interpolation or modest extrapolation of the experimental data with respect to temperature and thus enabled the use of a single, consistent temperature value for solubility evaluation, despite the experimental sources covering different temperature ranges. Therefore, the reference data used in this work can be termed “pseudoexperimental”, although this terminology is not used consistently further in the text.

9 ln *x*^SLE,exp^_a_ = *A* + *B*/*T* + *C* ln *T*

In eqn (9), *A*, *B*, and *C* are adjustable parameters fitted to experimental solubility data. Values of the Apelblat parameters *A*, *B*, and *C* used in this work are provided in the SI (Table S2).

To evaluate the quantitative performance of COSMO-SAC, we used the following three measures of deviations between pseudoexperimental solubilities (*x*^SLE,exp^_a_) and those calculated using COSMO-SAC (*x*^SLE^_a_):10
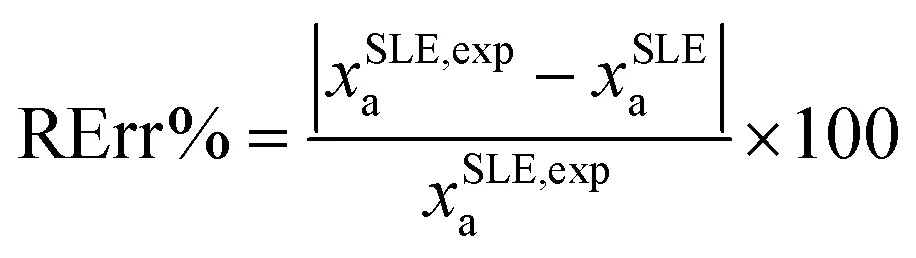

11 log_10_ Err = |log_10_ *x*^SLE,exp^_a_ − log_10_ *x*^SLE^_a_|

12 AErr = |*x*^SLE,exp^_a_ − *x*^SLE^_a_|

The average values of the quantities shown in eqn (10)–(12) over specific datasets were obtained as the corresponding sums divided by the number of considered data points.

To complement the point-wise error metrics with an explicit measure of solvent-ranking performance, we also report the Pearson (*r*_P_, eqn (13)) and Spearman (*r*_S_, eqn (14)) correlation coefficients between pseudoexperimental and calculated solubilities:13
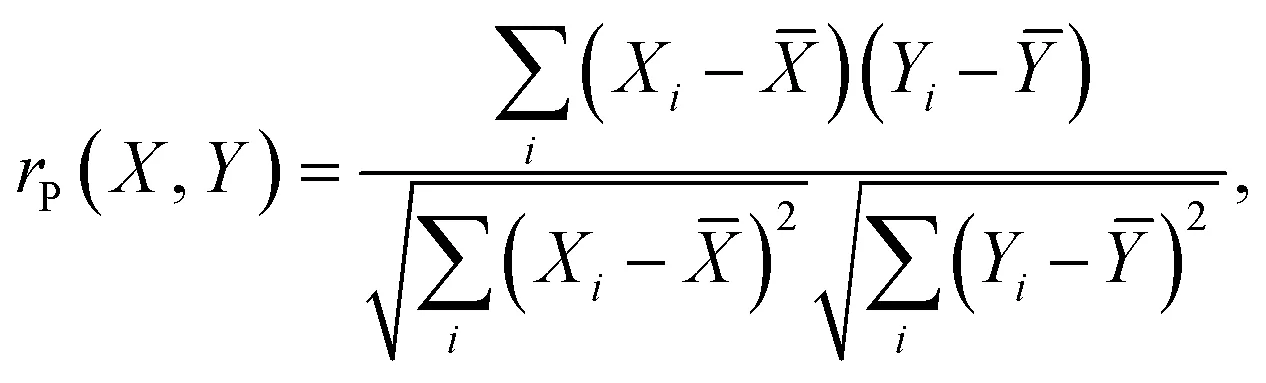

14 *r*_S_(*X*, *Y*) = *r*_P_(rank(*X*), rank(*Y*)).

The Pearson coefficient quantifies the strength of the linear relationship between two variables *X* and *Y*, while the Spearman coefficient is defined as the Pearson coefficient evaluated on the ranks of the data and therefore captures any monotonic, not necessarily linear, relationship. In this work, we used logarithmic transformation of solubilities: *X*_*i*_ = log_10_ *x*^SLE,exp^_a,*i*_ and *Y*_*i*_ = log_10_ *x*^SLE^_a,*i*_, because the values span several orders of magnitude. By definition, the value of *r*_S_ directly reflects how well the model ranks the solvents.

## 3 Results and discussion

In the following section, we evaluate the performance of the open-source COSMO-SAC model for solvent screening of polymer additives, both qualitatively and quantitatively, by comparing equilibrium solubilities (*x*^SLE^_a_; eqn (3)) calculated by COSMO-SAC with their pseudoexperimental counterparts. Next, we compare the performance of our COSMO-SAC approach with highly debated ML-derived activity coefficient model, HANNA. Finally, Section 3.2 presents an analysis of the sensitivity to different modeling details and discusses broader aspects, including the suitability of various thermodynamic descriptors for solvent screening. For clarity, the term “COSMO-SAC” used below refers to the COSMO-SAC-2010 model (eqn (8)), while a comparison between COSMO-SAC-2010 and COSMO-SAC-dsp (eqn (7)) is included in Section 3.2. Solvent abbreviations used in the figures are listed in Table S1 in the SI.

### 3.1 Solvent screening for the considered additives

#### 3.1.1 BHT

BHT is one of the simplest additives considered in this study. Yet, this does not necessarily mean that capturing its interactional behavior is straightforward. Its interactions are mainly governed by the benzene ring, the hydroxyl group, and their interplay. Compared with less substituted phenol derivatives, however, the effect of the OH group and the resulting HB in BHT is more attenuated by the large steric hindrance from the adjacent *tert*-butyl groups.^64,93^

**Fig. 2:**
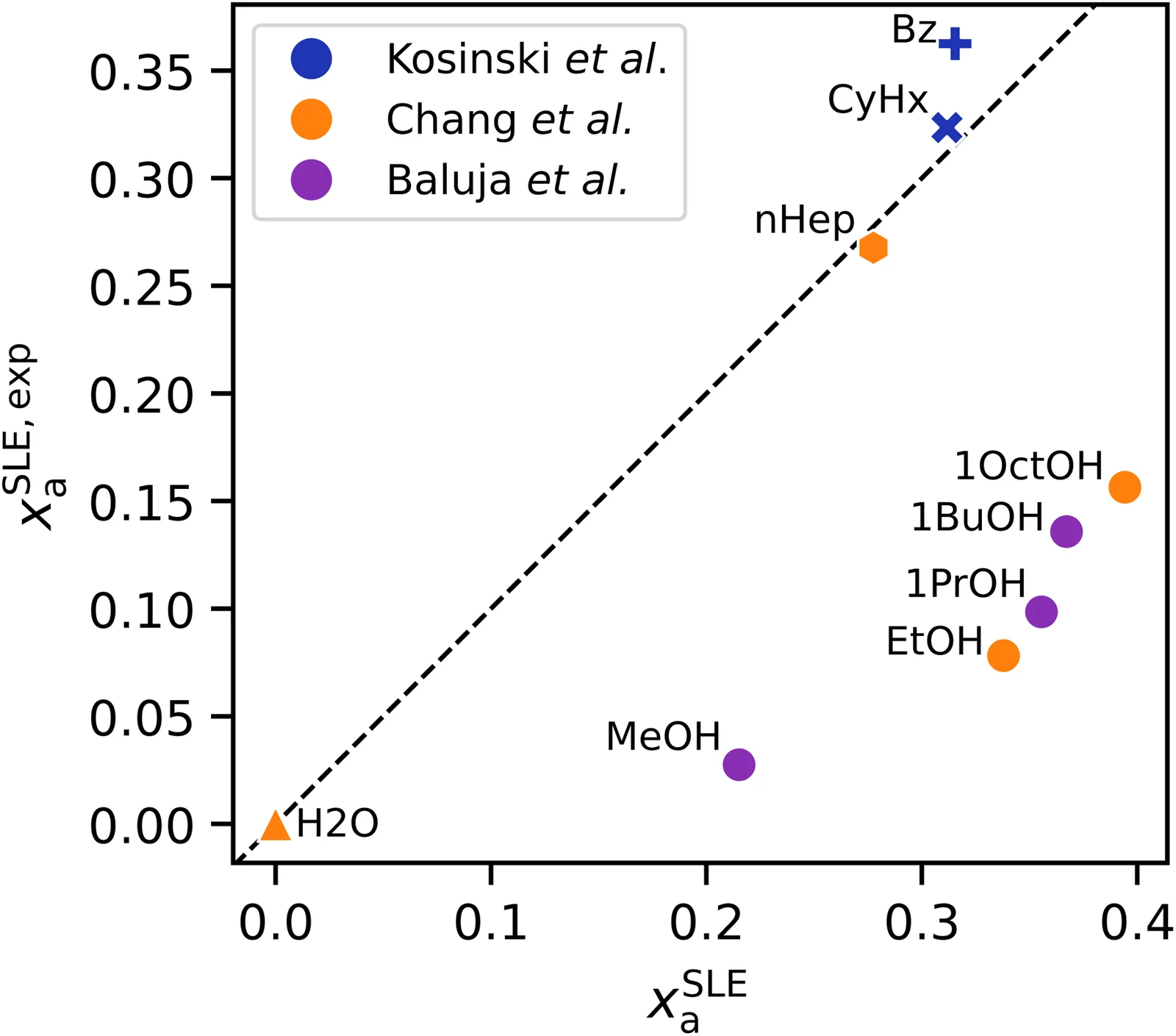
Parity plot comparing COSMO-SAC-predicted BHT solubilities with experimental data^57,64,88^ at 293 K.

Experimentally, BHT shows higher solubilities in nonpolar solvents, such as benzene and *n*-heptane, than in 1-alkanols, as illustrated in Fig. 2 and Table 4. This also correlates with the fact that, within the 1-alkanol series, the solubility of BHT increases with the length of the alkyl chain.^88^

**Table 4:** Solubility data for BHT at 293 K and the corresponding COSMO-SAC errors

Solvent	*x* ^SLE,exp^_a_	*x* ^SLE^_a_	RErr%	log_10_ Err	AErr	Source
Water	1.23 × 10^−7^	7.47 × 10^−9^	93.9	1.21	1.15 × 10^−7^	Chang *et al.*^57^
Methanol	0.0275	0.215	682	0.893	0.188	Baluja *et al.*^88^
Ethanol	0.0782	0.338	332	0.636	0.260	Chang *et al.*^57^
1-Propanol	0.0985	0.356	261	0.557	0.257	Baluja *et al.*^88^
1-Butanol	0.136	0.367	170	0.432	0.231	Baluja *et al.*^88^
1-Octanol	0.156	0.394	152	0.402	0.238	Chang *et al.*^57^
*n*-Heptane	0.268	0.278	3.68	0.0157	0.00986	Chang *et al.*^57^
Cyclohexane	0.324	0.312	3.66	0.0162	0.0118	Kosinski *et al.*^64^
Benzene	0.363	0.315	13.0	0.0604	0.0470	Kosinski *et al.*^64^

In contrast to the experimental observations, COSMO-SAC predicts BHT to be slightly more soluble in 1-alkanols than in nonpolar solvents. Though, the predictions capture the relative trends within each solvent group very well (*n*-heptane < cyclohexane < benzene; methanol < ethanol < … < 1-octanol). Furthermore, water is correctly identified as the poorest solvent for BHT.

Regarding quantitative aspects (see Table 4), the largest COSMO-SAC errors are observed for solubilities in 1-alkanols, gradually increasing with decreasing number of carbon atoms. This suggests that the description of BHT–1-alkanol interactions is responsible for the incorrect relative ranking between the nonpolar and 1-alkanol solvent groups. Since COSMO-SAC predicts significantly higher solubilities in 1-alkanols than observed experimentally, it appears to overestimate the strength of HB between BHT and 1-alkanol molecules. Finally, COSMO-SAC correctly captures the drastic drop in solubility when switching from alcohols to water. The corresponding error for the aqueous solubility of BHT, around one log unit, may appear large; however, the reference value is only a qualified estimate (because a reliable solubility determination is challenging in this case^57^), whose uncertainty may itself be considerable. Furthermore, the values from other sources lack mutual consistency.^57,88,94^ Consequently, no firm conclusion can be drawn regarding the COSMO-SAC error for the BHT–water system.

#### 3.1.2 ODP

Like BHT, ODP is a phenol derivative with the *t*Bu–OH–*t*Bu sequence, but with a much longer and more complex 3-(octadecylpropionate) substituent at the *para* position, introducing additional nonpolar and, at the same time, HB-accepting (OT) surface regions for interaction with solvents.

**Fig. 3:**
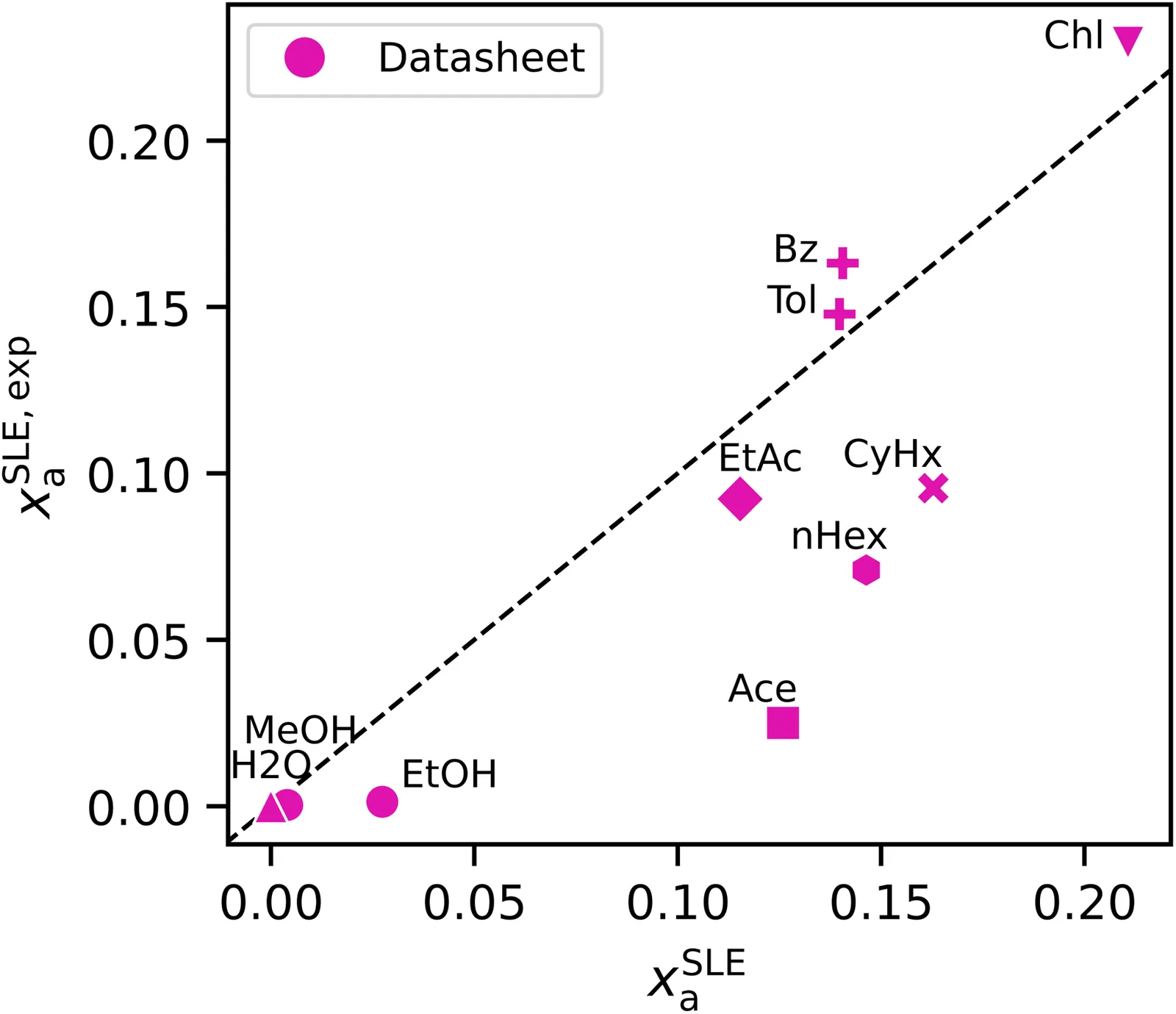
Parity plot comparing COSMO-SAC-predicted ODP solubilities with experimental data^70^ at 293 K.

As described in Section 2.4, experimental solubilities of ODP were taken from the respective product datasheet.^70^ Unlike for the other additives considered, this is the only available source, as no quantitatively reliable alternatives are known to us. As a result, the reference experimental solubilities and the corresponding solvent ranking should be interpreted with somewhat greater caution in the case of ODP. The experimental and calculated solubilities for ODP are summarized in Table 5 and visualized in Fig. 3.

**Table 5:** Solubility data for ODP at 293 K and the corresponding COSMO-SAC errors

Solvent	*x* ^SLE,exp^_a_	*x* ^SLE^_a_	RErr%	log_10_ Err	AErr	Source
Water	<3 × 10^−7^	2.54 × 10^−19^	n.d.^a^	n.d.^a^	n.d.^a^	Datasheet^70^
Methanol	0.000364	0.00401	1001	1.04	0.00365	Datasheet^70^
Ethanol	0.00132	0.0274	1975	1.32	0.0261	Datasheet^70^
Acetone	0.0250	0.126	403	0.702	0.101	Datasheet^70^
*n*-Hexane	0.0710	0.146	106	0.314	0.0754	Datasheet^70^
Ethyl acetate	0.0923	0.115	25.0	0.0967	0.0230	Datasheet^70^
Cyclohexane	0.0956	0.163	70.4	0.231	0.0673	Datasheet^70^
Toluene	0.148	0.140	5.46	0.0244	0.00808	Datasheet^70^
Benzene	0.163	0.141	13.9	0.0647	0.0226	Datasheet^70^
Chloroform	0.229	0.211	8.15	0.0369	0.0187	Datasheet^70^

a Not determined as an explicit experimental value is not available.

Similarly to BHT, ODP experimentally shows higher solubilities in hydrocarbons, while those in alcohols and water are considerably lower. The highest solubility among the considered solvents is observed in chloroform, which could be attributed to the HB-accepting ester group in ODP interacting with HB-donating chloroform, along with additional favorable NHB contributions. All these qualitative observations are successfully captured by COSMO-SAC. However, some detailed inconsistencies appear in the predicted ranking for solvents with moderate affinity to ODP (*e.g.*, COSMO-SAC predicts alkanes to be better solvents for ODP than aromatics, whereas the opposite is observed experimentally).

Quantitatively, the predicted solubility values in chloroform and benzene are in excellent agreement with experiment, whereas those in polar solvents show larger deviations—up to one or more orders of magnitude. The error for the aqueous ODP solubility could not be evaluated because an explicit experimental solubility value^70^ is not available. In this case, our assessment is limited to noting that COSMO-SAC qualitatively ranks water as the poorest solvent for ODP.

#### 3.1.3 TBP

TBP is a structurally complex molecule consisting of a central phosphite ester group bonded to three *tert*-butylated benzene rings. It is the only antioxidant in our validation set that does not contain any HB-donor groups. The presence of the phosphite group introduces a strong HB-acceptor site, while the bulky *tert*-butyl groups contribute to steric hindrance, potentially affecting the accessibility of the HB-acceptor site.

In the experimental data set for TBP, we have a combination of well-reported data from Yang *et al.*^59^ and data from a technical datasheet^90^ with a certain level of uncertainty, as described in Section 2.4. Fortunately, both sources report solubilities in ethyl acetate, acetone, and *n*-hexane, all of which are in good agreement.

Overall, TBP shows moderate to low solubilities in most of the considered solvents, with the highest solubility observed in chloroform, followed by dichloromethane, and toluene. The solubility in esters generally increases with increasing alkyl chain length. In the *n*-alkane series, the solubility of TBP decreases with increasing chain length. Low solubilities are observed in acetone, followed by alcohols and water. The experimental solubilities in water and methanol are only reported with the “less than” symbol, indicating very low values.

**Fig. 4:**
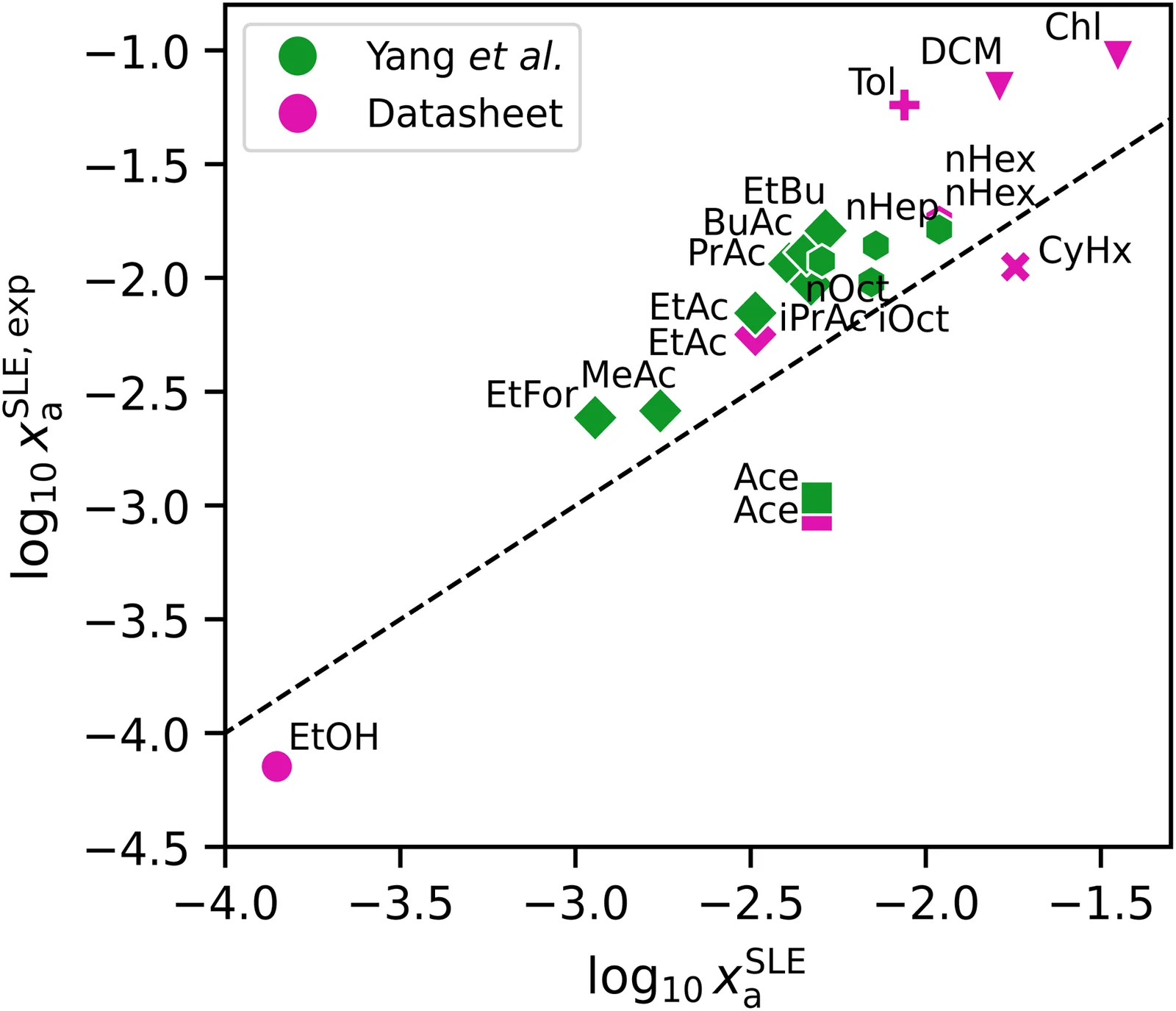
Parity plot comparing COSMO-SAC-predicted TBP solubilities with experimental data^59,90^ at 293 K. Full range except water and methanol.

The COSMO-SAC solubility predictions for TBP represent a typical outcome of COSMO-based solvent screenings (as in ref. 9, 33, 35 and 95 for example). Qualitatively, the model captures the overall trends and rankings of solvent effectiveness reasonably well (see Fig. 4). For instance, water is correctly identified as the poorest and chloroform as the best solvent for TBP. The ranking within homologous series, such as esters and *n*-alkanes, is also satisfactorily reproduced. However, some discrepancies in the detailed ranking of solvents with moderate affinity to TBP are observed.

Quantitatively, COSMO-SAC underpredicts the solubility of TBP in most solvents, with acetone being the only notable exception, where a significant overprediction is observed. Overall, the errors (see Table 6) are surprisingly low, given the structural complexity of TBP. As in the ODP–water system discussed in the previous section, the errors for TBP solubilities in water and methanol could not be quantified due to the absence of explicit experimental values.^90^ Consequently, we can only conclude that COSMO-SAC correctly identifies both as the poorest solvents for TBP.

**Table 6:** Solubility data for TBP at 293 K and the corresponding COSMO-SAC errors

Solvent	*x* ^SLE,exp^_a_	*x* ^SLE^_a_	RErr%	log_10_ Err	AErr	Source
Water	<3 × 10^−7^	3.99 × 10^−24^	n.d.^a^	n.d.^a^	n.d.^a^	Datasheet^90^
Methanol	<5 × 10^−6^	1.56 × 10^−5^	n.d.^a^	n.d.^a^	n.d.^a^	Datasheet^90^
Ethanol	7.13 × 10^−5^	1.40 × 10^−4^	96.9	0.294	6.91 × 10^−5^	Datasheet^90^
Acetone	0.000906	0.00488	439	0.731	0.00398	Datasheet^90^
Acetone	0.00108	0.00488	352	0.655	0.00380	Yang *et al.*^59^
Ethyl formate	0.00243	0.00114	53.2	0.330	0.00129	Yang *et al.*^59^
Methyl acetate	0.00261	0.00175	33.0	0.174	0.000860	Yang *et al.*^59^
Ethyl acetate	0.00564	0.00326	42.2	0.238	0.00238	Datasheet^90^
Ethyl acetate	0.00701	0.00326	53.4	0.332	0.00374	Yang *et al.*^59^
Isopropyl acetate	0.00939	0.00470	50.0	0.301	0.00469	Yang *et al.*^59^
Isooctane	0.00957	0.00699	26.9	0.136	0.00258	Yang *et al.*^59^
Cyclohexane	0.0112	0.0180	61.0	0.207	0.00683	Datasheet^90^
Propyl acetate	0.0115	0.00407	64.6	0.451	0.00744	Yang *et al.*^59^
*n*-Octane	0.0119	0.00505	57.4	0.370	0.00680	Yang *et al.*^59^
Butyl acetate	0.0129	0.00457	64.7	0.452	0.00838	Yang *et al.*^59^
*n*-Heptane	0.0139	0.00720	48.1	0.285	0.00668	Yang *et al.*^59^
Ethyl butyrate	0.0161	0.00517	67.9	0.493	0.0109	Yang *et al.*^59^
*n*-Hexane	0.0163	0.0109	33.1	0.175	0.00540	Yang *et al.*^59^
*n*-Hexane	0.0178	0.0109	38.8	0.213	0.00692	Datasheet^90^
Toluene	0.0575	0.00869	84.9	0.821	0.0488	Datasheet^90^
Dichloromethane	0.0688	0.0162	76.4	0.627	0.0525	Datasheet^90^
Chloroform	0.0940	0.0353	62.4	0.425	0.0587	Datasheet^90^

a Not determined as an explicit experimental value is not available.

#### 3.1.4 NPH

NPH is the most structurally complex antioxidant in our validation set. It is composed of a pentaerythritol core with four 3-(3,5-di-*tert*-butyl-4-hydroxyphenyl)propionate arms attached *via* ester bonds. This structure introduces four *tert*-butylated phenol sequences contributing to the HB sites (OH), as well as four ester groups providing HB acceptor surface regions (OT).

As in the case of TBP, we have a combination of data from Wei *et al.*^60,89^ and data from a technical datasheet^91^ for which we assume certain level of uncertainty. The datasets report similar solubility in methanol, but they differ significantly in the reported solubility values for ethanol and *n*-hexane, with the datasheet reporting almost double the solubility in both cases. However, rankings from both data sets qualitatively align: *n*-hexane < methanol < ethanol. Interestingly, the technical datasheet values are closer to the COSMO-SAC predictions.

Experimentally, NPH shows the highest solubilities in chloroform and dichloromethane, followed by aprotic solvents—ethyl acetate and acetone, whereas that for alcohols and *n*-hexane is the smallest within the dataset. Overall, the mole fraction solubilities are the lowest among the considered additives.

**Fig. 5:**
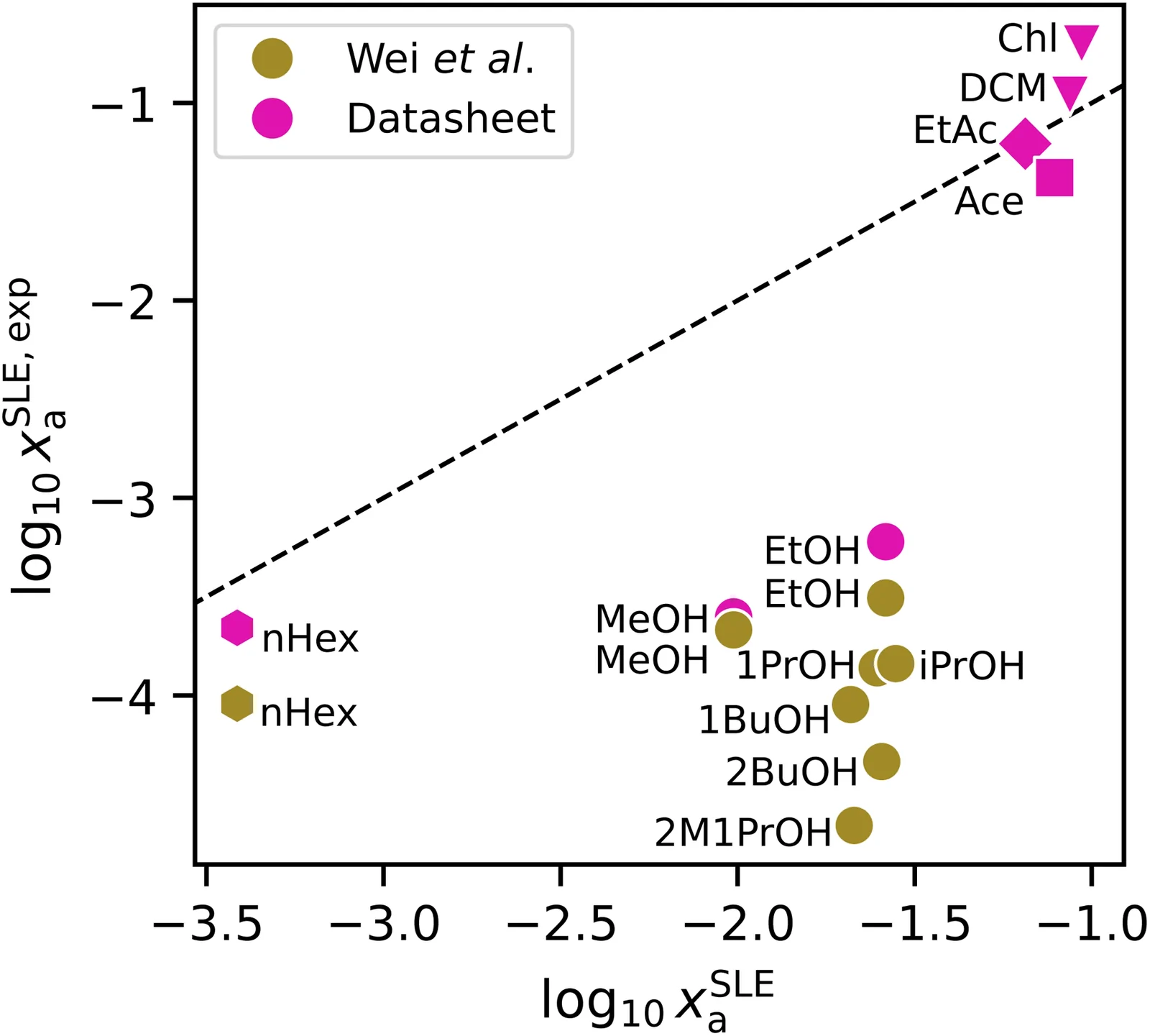
Parity plot comparing COSMO-SAC-predicted NPH solubilities with experimental data^89,91^ at 293 K.

COSMO-SAC generally tends to overestimate the solubility of NPH. The numerical error for NPH is much higher than for other additives, but this is typical for low solubility compounds, where errors of 1–2 log units are common.^33,35^ Despite NPH's complexity, the ranking of solvents from the technical datasheet is impressively reproduced, while the ranking from ref. 60 and 89 is somewhat less accurate—likely because only similar solvents (alcohols) are considered, making subtle structural and *σ*-profile differences more significant and harder to capture, possibly due to the reduction of 3D to 2D information in the *σ*-profile generation, as described in Section 2.2. However, combining both datasets together, COSMO-SAC correctly captures that NPH is globally less soluble in alcohols than in chlorinated hydrocarbons, ethyl acetate, and acetone. The experimental and calculated solubilities for NPH are summarized in Table 7 and visualized in Fig. 5. It is worth noting that, similar to the BHT case shown in Fig. 2, COSMO-SAC significantly overestimates solubility in alcohols.

**Table 7:** Solubility data for NPH at 293 K and the corresponding COSMO-SAC errors

Solvent	*x* ^SLE,exp^_a_	*x* ^SLE^_a_	RErr%	log_10_ Err	AErr	Source
2-Methyl-1-propanol	2.19 × 10^−5^	0.0213	97 100	2.99	0.0213	Wei *et al.*^89^
2-Butanol	4.62 × 10^−5^	0.0255	55 100	2.74	0.0255	Wei *et al.*^89^
1-Butanol	8.98 × 10^−5^	0.0208	23 100	2.37	0.0208	Wei *et al.*^89^
*n*-Hexane	9.08 × 10^−5^	3.86 × 10^−4^	325	0.629	2.95 × 10^−4^	Wei *et al.*^60^
1-Propanol	0.000138	0.0248	17 900	2.25	0.0246	Wei *et al.*^89^
Isopropyl alcohol	0.000145	0.028	19 200	2.29	0.0278	Wei *et al.*^89^
Methanol	0.000215	0.00974	4440	1.66	0.00953	Wei *et al.*^89^
*n*-Hexane	0.00022	0.000386	75.5	0.244	0.000166	Datasheet^91^
Methanol	0.00025	0.00974	3800	1.59	0.00949	Datasheet^91^
Ethanol	0.000311	0.0262	8310	1.92	0.0259	Wei *et al.*^89^
Ethanol	0.0006	0.0262	4260	1.64	0.0256	Datasheet^91^
Acetone	0.0419	0.0786	87.6	0.273	0.0367	Datasheet^91^
Ethyl acetate	0.0622	0.0649	4.36	0.0185	0.00271	Datasheet^91^
Dichloromethane	0.109	0.0868	20.7	0.100	0.0226	Datasheet^91^
Chloroform	0.199	0.0937	52.9	0.327	0.105	Datasheet^91^

#### 3.1.5 PPB

Finally, we discuss the results obtained for PPB. PPB is, together with BHT, the simplest additive in our set in terms of chemical structure (see Fig. 1). Like BHT, ODP, and NPH, it contains a phenolic OH group, but unlike the other additives, it is not sterically hindered by adjacent alkyl substituents. Therefore, the effect of the OH surface and the related HB character can be expected to be more pronounced.

This hypothesis is justified by the relatively high experimental solubilities in esters and alcohols listed in Table 8. The PPB solubility in acetonitrile is approximately three times lower than those in esters and alcohols, while its aqueous solubility is few orders of magnitude lower.

**Table 8:** Solubility data for PPB at 293 K and the corresponding COSMO-SAC errors

Solvent	*x* ^SLE,exp^_a_	*x* ^SLE^_a_	RErr%	log_10_ Err	AErr	Source
Water	2.38 × 10^−5^	4.58 × 10^−5^	92.8	0.285	2.20 × 10^−5^	Ouyang *et al.*^61^
Acetonitrile	0.0602	0.0618	2.75	0.0118	0.00165	Ouyang *et al.*^61^
1-Propanol	0.139	0.195	40.3	0.147	0.0560	Ouyang *et al.*^61^
2-Methyl-1-propanol	0.139	0.128	8.04	0.0364	0.0112	Ouyang *et al.*^61^
Ethanol	0.140	0.205	46.4	0.166	0.0649	Ouyang *et al.*^61^
Methanol	0.142	0.172	21.0	0.0826	0.0298	Ouyang *et al.*^61^
Isopropyl alcohol	0.151	0.207	37.6	0.138	0.0566	Ouyang *et al.*^61^
Methyl acetate	0.162	0.146	9.44	0.0430	0.0153	Ouyang *et al.*^61^
Butyl acetate	0.181	0.101	44.2	0.253	0.0798	Ouyang *et al.*^61^
1-Butanol	0.181	0.186	2.50	0.0107	0.00454	Ouyang *et al.*^61^
Ethyl acetate	0.200	0.142	29.2	0.150	0.0585	Ouyang *et al.*^61^
Acetone	0.264	0.205	22.5	0.111	0.0596	Ouyang *et al.*^61^

**Fig. 6:**
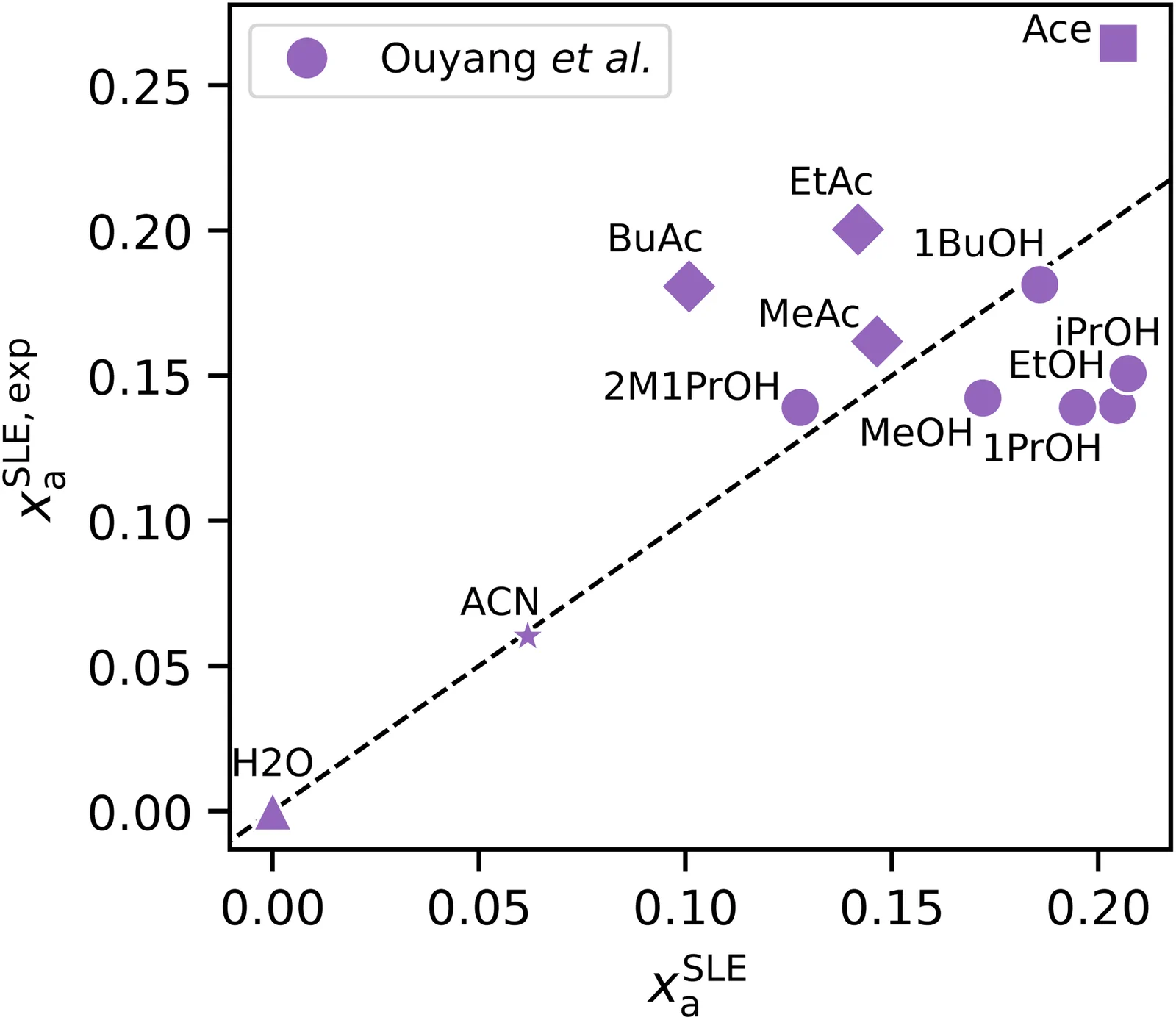
Parity plot comparing COSMO-SAC-predicted PPB solubilities with experimental data^61^ at 293 K.

The COSMO-SAC solubilities are compared against experimental ones in Fig. 6. COSMO-SAC again excellently ranks the global scale: acetone > alcohols ∼ esters > acetonitrile > water. Similarly to NPH, the solvent selection investigated in Ouyang *et al.*^61^ contains many structurally similar compounds with only minor differences between them (*e.g.*, structurally similar short-chain monohydric alcohols). The failure of COSMO-SAC ranking between similar alcohols and esters highlights that, for PPB and NPH, unlike other additives where different solvent classes were compared, subtle structural and *σ*-profile nuances play a much greater role in these cases and are challenging for even sophisticated models like COSMO-SAC to capture. (We emphasize that this is a matter of less diverse solvent sets and is not related to PPB and NPH themselves.) Thus, the predicted rankings should be interpreted globally (*e.g.*, identifying the best/worst solvent classes) rather than focusing on detailed local differences between similar compounds.

Regarding the unsatisfactory results in the case of solvents with similar affinity to PPB, it is important to note that (as also mentioned in Ouyang *et al.*^61^) the detailed order of solvents (from both experiment and prediction) can change with temperature, and if COSMO-SAC predicts a general temperature shift relative to experiment, it may be difficult to achieve fully satisfactory results at a single chosen temperature. This aspect is further elaborated in Section 3.2.2.

#### 3.1.6 Overall observations

Summarizing the COSMO-SAC predictions for all five antioxidant additives, a general trend of overestimation is observed for BHT, ODP, and NPH, while TBP solubility is underestimated (PPB shows mixed results). This pattern may be attributed to the presence of the bifunctional OH group, as TBP is the only additive lacking it and the associated HB donor character. Notably, this observation is consistent with previous reports that COSMO-type models tend to overpredict solubilities for, *e.g.*, pharmaceuticals and other structurally similar compounds.^35^

**Fig. 7:**
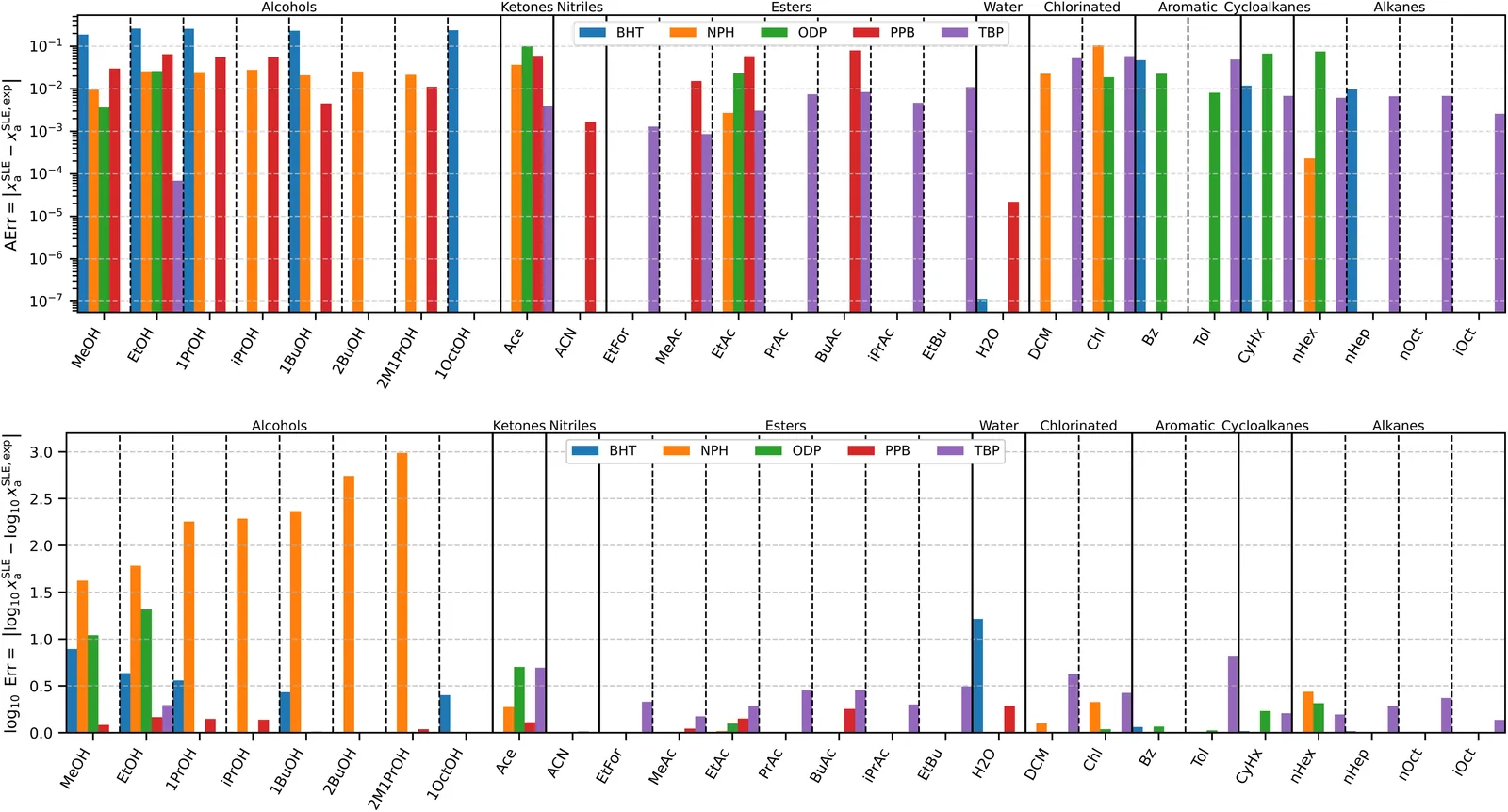
Absolute (top) and log_10_ (bottom) errors of the solubility predictions obtained from COSMO-SAC.

Fig. 7 presents the relative, absolute, and logarithmic errors of COSMO-SAC solubility predictions for all additives across different solvent classes. The numerical values of the corresponding overall errors are summarized in Tables 9–11, respectively. Water is consistently and correctly identified as the poorest solvent for all additives. However, quantitative error assessment for aqueous systems is limited due to the absence of exact experimental values for ODP and TBP, preventing meaningful error calculations. Nevertheless, for BHT and PPB, where the experimental values carry less uncertainty, the errors are comparable to those observed for other solvents.

**Table 9:** Overall absolute error (eqn (12)) for all investigated additives

	BHT	NPH	ODP	PPB	TBP
Alcohol	0.235	0.0222	0.0149	0.0372	6.91 × 10^−5^
Alkane	0.00986	0.000231	0.0754		0.00556
Aromatic hydrocarbon	0.0470		0.0153		0.0488
Cycloalkane	0.0118		0.0673		0.00683
Ester		0.00271	0.0230	0.0512	0.00524
Ketone		0.0367	0.101	0.0596	0.00389
Nitrile				0.00165	
Organochlorine		0.0639	0.0187		0.0556
Water	1.15 × 10^−7^			2.20 × 10^−5^	
Overall average	0.138	0.0239	0.0384	0.0365	0.0121

**Table 10:** Overall log_10_ error (eqn (11)) for all investigated additives

	BHT	NPH	ODP	PPB	TBP
Alcohol	0.584	2.29	1.18	0.0968	0.294
Alkane	0.0157	0.436	0.314		0.246
Aromatic hydrocarbon	0.0604		0.0446		0.821
Cycloalkane	0.0162		0.231		0.207
Ester		0.0185	0.0967	0.149	0.355
Ketone		0.273	0.702	0.111	0.693
Nitrile				0.0118	
Organochlorine		0.214	0.0369		0.526
Water	1.21			0.285	
Overall average	0.470	1.40	0.426	0.120	0.386

**Table 11:** Overall relative error (RErr%; eqn (10)) for all investigated additives

	BHT	NPH	ODP	PPB	TBP
Alcohol	3.20	318	14.9	0.260	0.969
Alkane	0.0368	2.00	1.06		0.421
Aromatic hydrocarbon	0.130		0.0966		0.849
Cycloalkane	0.0366		0.704		0.610
Ester		0.0436	0.250	0.276	0.545
Ketone		0.876	4.03	0.225	3.96
Nitrile				0.0275	
Organochlorine		0.368	0.0815		0.694
Water	0.939			0.928	
Overall average	1.90	156	4.01	0.297	0.903

Alcohols generally display higher prediction errors compared to other solvent classes, which may be related to the simplified treatment of HB in the COSMO-SAC model; however, recent developments have begun to address this limitation.^96^ It should be noted that the large errors observed for alcohols are primarily associated with the generally higher errors found in systems where NPH is the solute. Consequently, it is unclear whether these deviations should be attributed to the alcohols or to NPH itself. In contrast, most other solvent classes exhibit moderate errors (typically 0.5 log units or less).

Regarding individual additives, the highest COSMO-SAC errors are observed for NPH and the lowest for PPB. ODP, TBP and BHT exhibit comparable errors.

Finally, Table 12 reports the Pearson and Spearman correlation coefficients, the overall *r*_S_ value of 0.82 is a direct measure of the solvent-ranking performance.

**Table 12:** Correlation coefficients and log_10_ errors between pseudoexperimental and calculated solubilities for COSMO-SAC and HANNA

Additive	*n* a	COSMO-SAC				HANNA			
		*r*_P_ b	*r*_S_ c	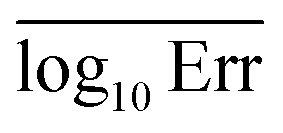 d	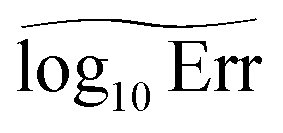 e	*r*_P_ b	*r*_S_ c	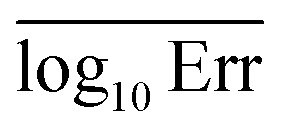 d	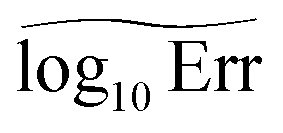 e
BHT	9	0.987	0.300	0.470	0.432	0.964	0.550	0.452	0.225
NPH	15	0.543	0.661	1.403	1.640	0.123	−0.217	1.509	0.993
ODP	10	0.903	0.842	1.590	0.273	0.950	0.927	0.683	0.705
PPB	12	0.993	0.413	0.120	0.125	0.981	0.720	0.449	0.555
TBP	22	0.785	0.834	1.140	0.351	0.725	0.229	1.407	1.352
Overall	68	0.691	0.818	0.995	0.321	0.780	0.780	1.028	0.807

a Number of additive–solvent–temperature points.

b Pearson correlation coefficient (eqn (13)), evaluated on log_10_ *x*^SLE^_a_.

c Spearman rank correlation coefficient (eqn (14)).

d Mean of the per-point log_10_ error (eqn (11)).

e Median of the per-point log_10_ error.

#### 3.1.7 COSMO-SAC *vs.* ML model HANNA

**Fig. 8:**
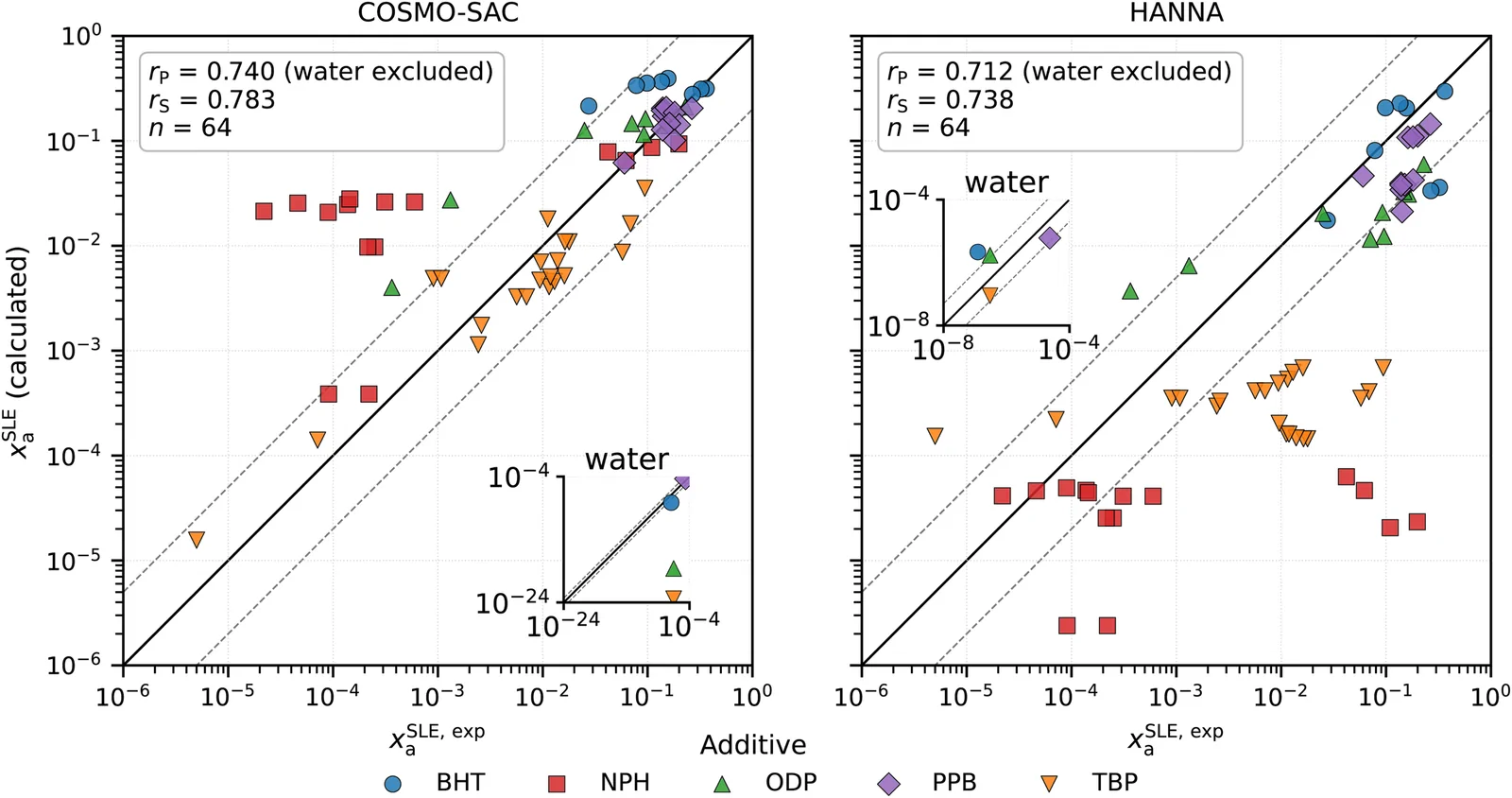
Parity plot of experimental *vs.* calculated solubilities for COSMO-SAC (left) and HANNA (right). Each marker represents one additive–solvent solubility at 293 K (solvent names omitted). The dashed lines indicate ±0.5 log unit deviation. Aqueous systems are shown separately. Annotations report Pearson (*r*_P_, on log_10_ *x*^SLE^_a_) and Spearman (*r*_S_) correlation coefficients for the non-aqueous subset, with *n* the number of data points, the corresponding coefficients including water are listed in Table 12.

The HANNA model^17^ is a machine learning model for the molar excess Gibbs energy that predicts thermodynamically consistent activity coefficients solely from molecular structures (provided as SMILES strings) and the state point (temperature and composition). The model was trained on a large dataset of (VLE, LLE, *H*^E^, and ln *γ*^∞^ data) from the DDB database and strictly addresses thermodynamic consistency. When the activity coefficients from HANNA are combined with eqn (3), it allows us to use the same fusion properties as in our COSMO-SAC predictions and to directly compare the two approaches. The results of this comparison are summarized in Table 12 and visualized in Fig. 8. Overall, both models show comparable performance in terms of solvent ranking (overall *r*_S_: 0.82 (COSMO-SAC) *vs.* 0.78 (HANNA)), but COSMO-SAC shows significantly better prediction accuracy in the absolute values (log_10_ errors), interestingly, HANNA shows a systematic underestimation of solubilities, that are pronounced in bigger solutes (NPH and TBP) and also, the predicted activities are somewhat too high (*i.e.*, HANNA systematically underestimates solubility, as opposed to COSMO-based models).

### 3.2 General considerations and sensitivity analyses

In this section, selected “degrees of freedom” (*i.e.*, choices in modeling scenario or computational setup that a modeler has to make when developing COSMO-based solvent screening approach) are examined. Analyses of some of these degrees—namely, the dispersion contribution of COSMO-SAC and *σ*-profiles—turned out to be relatively less significant for the selection of additives considered in this work, which aligns with previous observations.^9,35,97^ Therefore, they are included only in the SI.

#### 3.2.1 Efficiency of different solubility descriptors

**Fig. 9:**
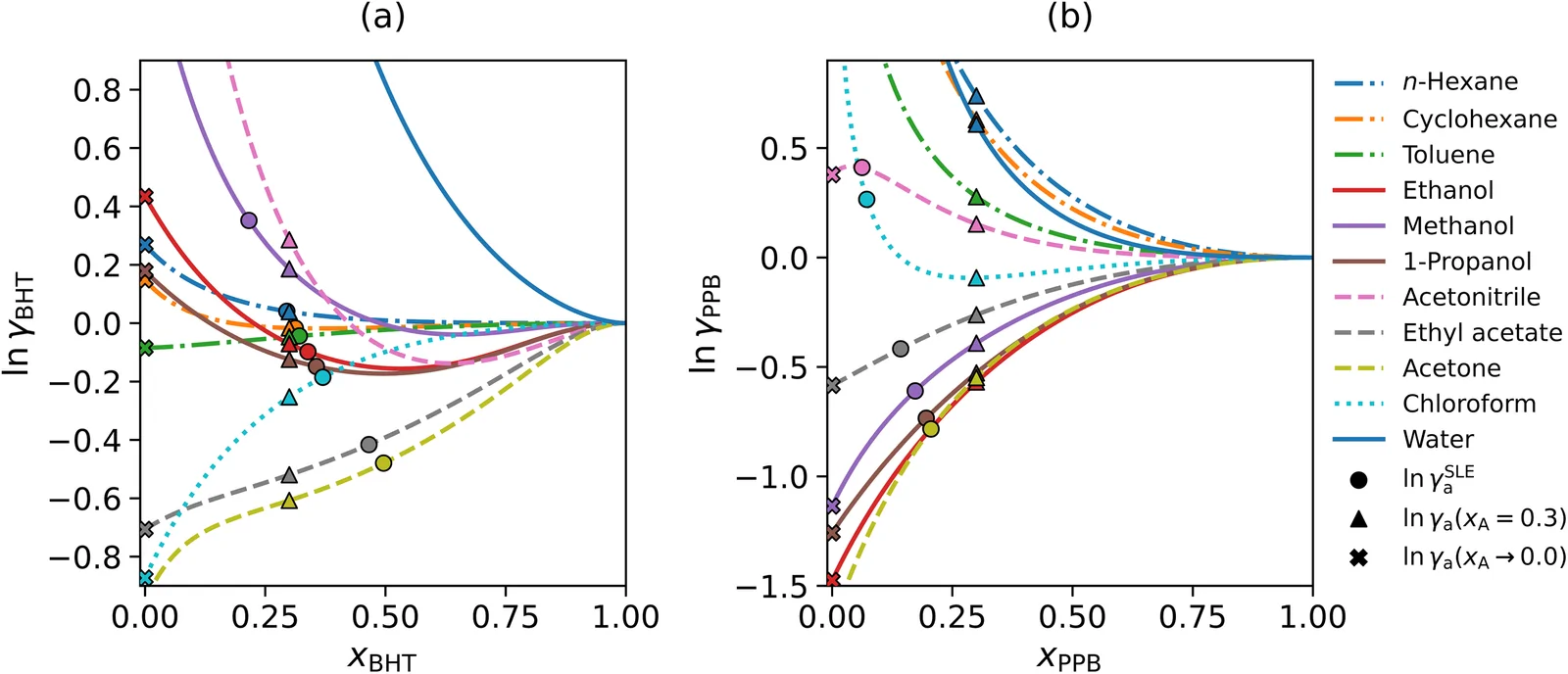
The composition dependence of ln *γ*_a_ at 293 K for (a) BHT and (b) PPB in various solvents. The points represent ln *γ*_a_ values at infinite dilution (*x*_a_ → 0), *x*_a_ = 0.3, and saturation (*i.e.*, equilibrium solubility).

In the previous sections, we have primarily focused on evaluating COSMO-SAC solubility predictions through direct comparison of calculated and experimental mole fraction solubilities (*x*^SLE^_a_*vs. x*^SLE,exp^_a_). However, as outlined in Section 2.1, a question arises whether alternative descriptors, such as ln *γ*^∞^_a_, can reproduce the qualitative solvent ranking. To investigate this, we calculated ln *γ*_a_ for each additive–solvent pair at three different compositions: infinite dilution (*x*_a_ → 0), *x*_a_ = 0.3, and saturation (equilibrium solubility). To further illustrate the composition dependence of ln *γ*_a_, Fig. 9 shows ln *γ*_a_ as a function of composition for BHT and PPB in various solvents. The behavior of ln *γ*_a_ with composition is generally complex, the curves are non-monotonic, and even switch from negative to positive deviations from Raoult's law within the composition range (*i.e.*, *γ*_*i*_ < 1 and *γ*_*i*_ > 1, respectively). This results in crossing of the ln *γ*_a_ curves for different solvents and highlights the challenges in using a single composition point to represent solubility behavior.

Bearing this complexity in mind, we evaluated the solvent rankings based on ln *γ*_a_ at the infinite dilution (principally corresponding to low solubility) and *x*_a_ = 0.3 (high solubility), and compared them to the rankings obtained earlier in this work from *x*^SLE^_a_*vs. x*^SLE,exp^_a_. The ranking plots are provided in Fig. S7–S11 in the SI. For low solubility additives (TBP, NPH), the ranking based on ln *γ*^∞^_a_ closely resembles that from *x*^SLE^_a_*vs. x*^SLE,exp^_a_, while for the others (BHT, ODP and PPB), the ranking based on ln *γ*_a_ at *x*_a_ = 0.3 is more appropriate. This can serve as a rule of thumb in situations where solvent screening is to be performed for a newly designed additive whose fusion properties are not available. For example, if low solubility is expected (based on, *e.g.*, structural considerations), ln *γ*^∞^_a_ would likely be the most appropriate descriptor.

The second widely used descriptor is the excess enthalpy *H*^E^. While *H*^E^ has been employed in solvent screening studies,^34,44^ its interpretation as a solubility descriptor requires caution. Unlike the activity coefficient, which captures both enthalpic and entropic contributions to the Gibbs energy, *H*^E^ reflects only the enthalpic favorability of mixing, as explained in Section 2.1. Consequently, *H*^E^-based rankings may deviate from solubility-based rankings in systems where entropic effects play a significant role.

**Fig. 10:**
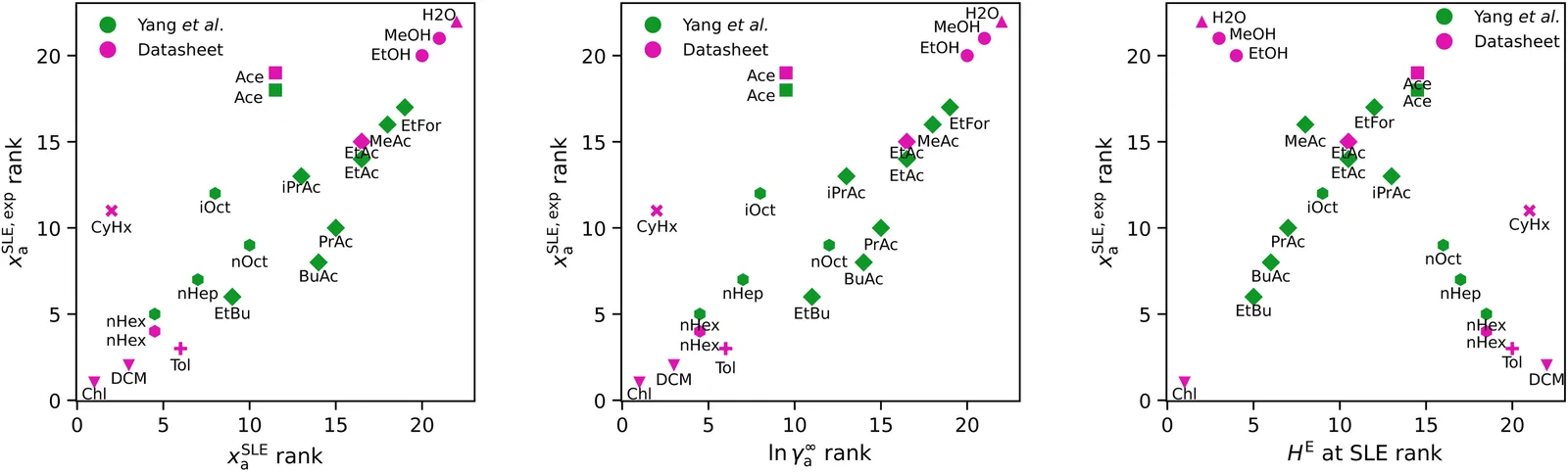
Comparison of solvent rankings at 293 K for TBP based on different COSMO-SAC descriptors *versus* experimental ranking: (left) equilibrium solubility *x*^SLE^_a_, (middle) infinite dilution activity coefficient ln *γ*^∞^_a_, (right) excess enthalpy at saturation *H*^E^(*x*^SLE^_a_).

In our evaluation, we found that *H*^E^ calculated at the SLE composition produced noticeably poorer solvent rankings compared to those based on ln *γ*_a_ or *x*^SLE^_a_, with systematic misclassification of certain solvent groups (see Fig. 10). While *H*^E^ may provide useful insights in limiting cases—such as systems close to the regular solution (with minimal entropic non-ideality)—it does not prove to be a reliable universal descriptor for solvent screening in the present context.

#### 3.2.2 Temperature dependence of solubility

The analysis of activity coefficients and solubility behavior becomes considerably more complex when not only composition but also temperature dependence is considered. As shown in Fig. 9a, the ln *γ*_a_ curves for different solvents can cross at various compositions; analogous crossings occur as a function of temperature.

**Fig. 11:**
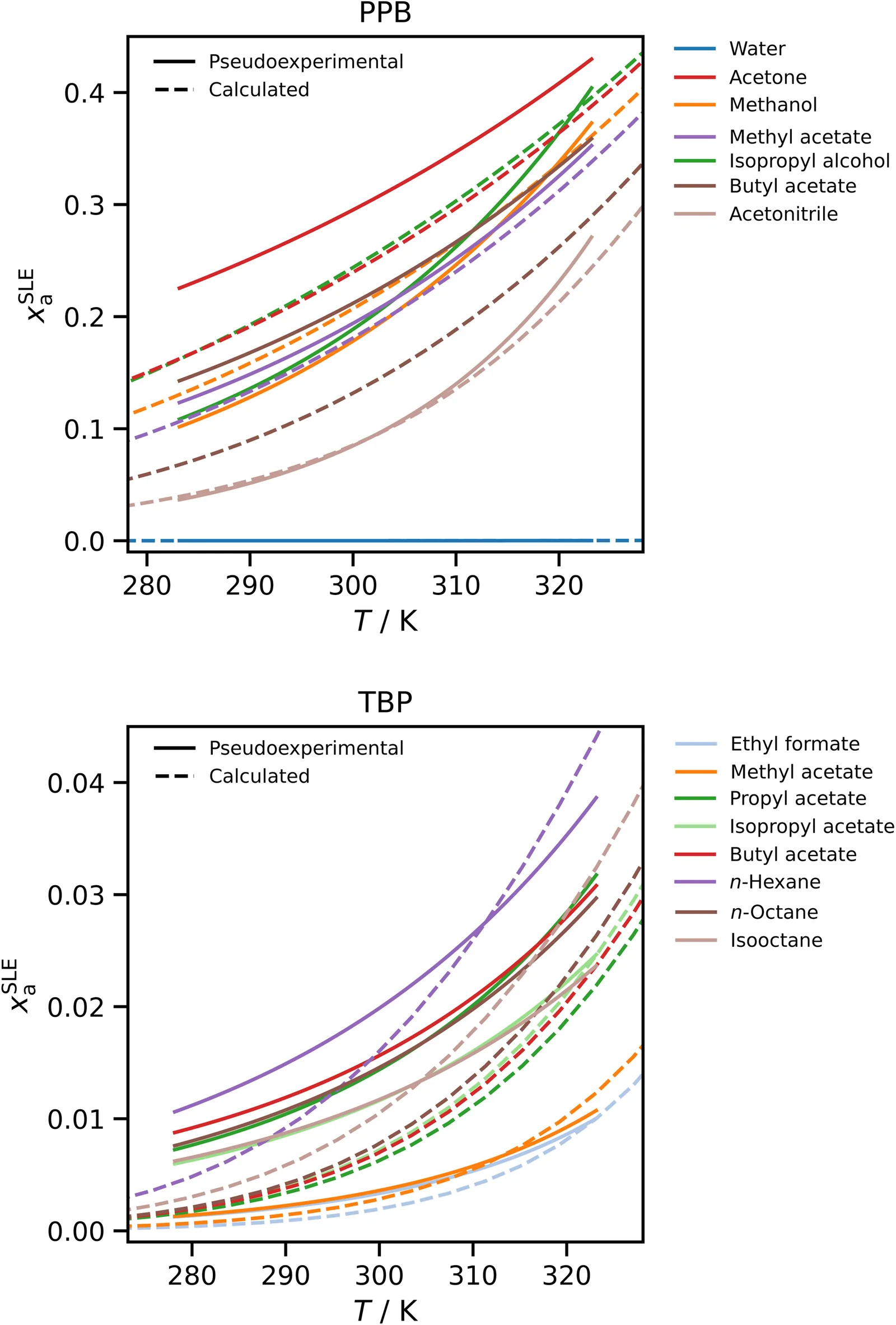
The temperature dependence of pseudoexperimental solubilities (solid lines) of PPB and TBP in various solvents compared to COSMO-SAC-predicted solubilities (dashed lines).

Fig. 11 shows the temperature course of pseudoexperimental solubilities (see Section 2.4) of PPB and TBP as examples in various solvents. Solubility curve crossings with temperature are particularly pronounced for PPB, with notable experimental crossings observed, for example, between isopropyl alcohol and butyl acetate near 310 K. In contrast, such crossings are less significant for TBP, where the solvent ranking remains more stable across the temperature range. The corresponding temperature dependencies predicted by COSMO-SAC are also shown in Fig. 11. While the model generally captures the qualitative trends, noticeable differences in the curvature of predicted solubility curves are observed for some systems.

It is important to note that the temperature dependence of activity coefficients in COSMO-SAC is relatively simple, relying on semi-empirical but universal parameters. Additionally, the temperature dependence of the HB contribution is not explicitly resolved. Consequently, COSMO-SAC predictions exhibit deviations from experiment, which can be more pronounced for certain solvent classes. As a result, predicted solubility curves and—more generally—any phase equilibria and mixing curves may be shifted relative to experiment. This can lead to curve crossings being predicted at different temperatures than observed experimentally—sometimes by several tens of Kelvins.

However, since all solubility curves must converge at the melting temperature, the range of possible deviations in solvent ranking is inherently constrained. This convergence also implies that curve crossings typically occur only among solvents with similar affinities to the solute. Thus, when modeling with COSMO-SAC, ranking results for compounds with similar solubility behavior—as discussed in Section 3.1.5—should be interpreted with caution, as subtle differences may fall below the resolution of a thermodynamic model, as observed for COSMO-SAC in this work.

#### 3.2.3 Sensitivity to thermodynamic fusion data

Not only does the accuracy of solubility predictions *via*eqn (2) depend on the quality of activity coefficient estimates, but also on the reliability of the thermodynamic fusion properties (*T*_m_, Δ*H*_fus_, and, Δ*C*_p,fus_) used as input. Assuming that the specific solid form of the solute is correctly identified, experimental uncertainties in the fusion quantities, as well as the choice of approximations for the heat capacity term in eqn (2), can introduce systematic errors into the predicted solubilities. In this section, we demonstrate and discuss the sensitivity to two strategies regarding Δ*C*_p,fus_ both quantitatively and qualitatively. A more comprehensive evaluation of different strategies regarding *T*_m_, Δ*H*_fus_ and Δ*C*_p,fus_ will be the subject of separate upcoming research.

**Fig. 12:**
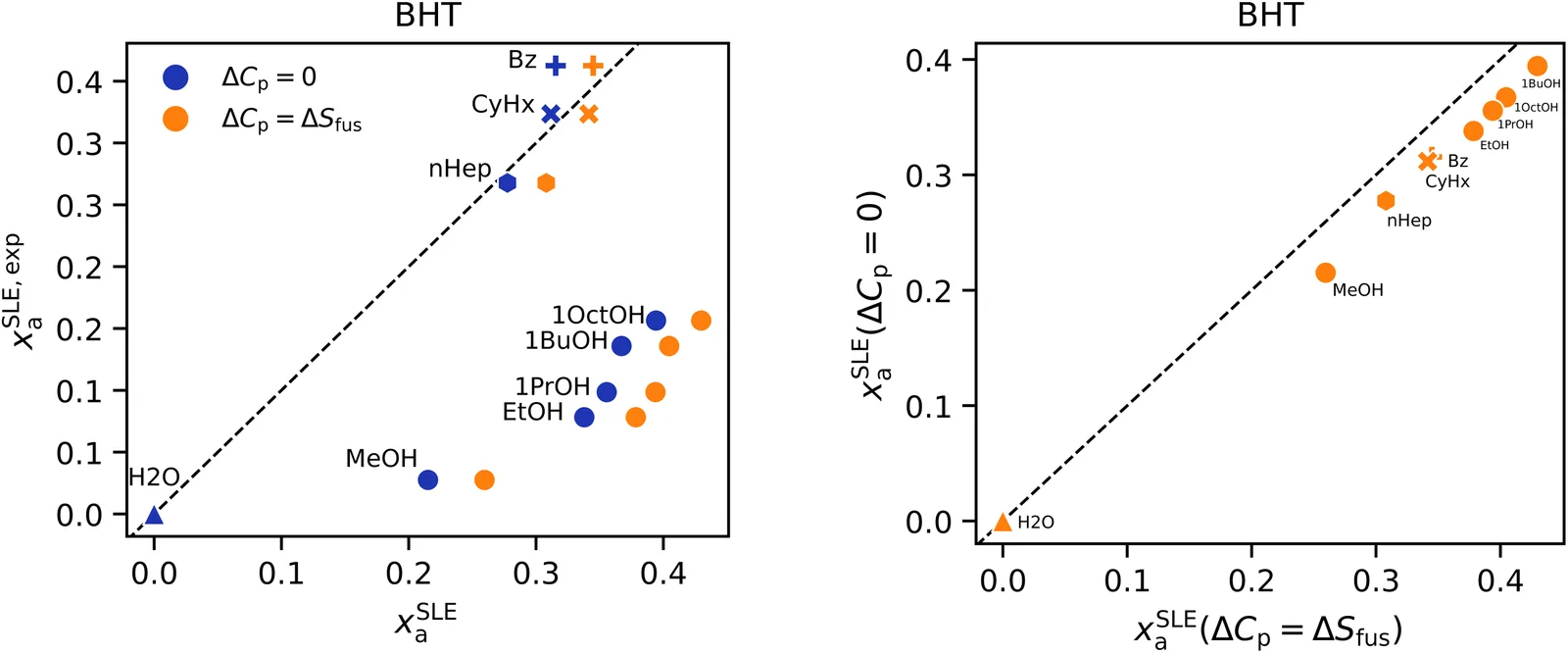
Comparison of COSMO-SAC solubility predictions for BHT at 293 K using two different approaches for Δ*C*_p,fus_: neglecting the heat capacity term (Δ*C*_p,fus_ = 0) *vs.* estimating it as Δ*C*_p,fus_ = Δ*S*_fus_. (Left) Parity plot comparing the COSMO-SAC *x*^SLE^_a_ predictions with experimental values *x*^SLE,exp^_a_; (right) parity plot comparing the two Δ*C*_p,fus_ approaches.

Since experimental values of Δ*C*_p,fus_ are unavailable for any of the considered additives, we evaluated the sensitivity of solubility predictions to two commonly employed approximations: (i) neglecting the heat capacity term entirely (*i.e.*, Δ*C*_p,fus_ = 0) and (ii) estimating it from the entropy of fusion (*i.e.*, Δ*C*_p,fus_ = Δ*S*_fus_), where Δ*S*_fus_ = Δ*H*_fus_/*T*_m_ (values provided in Table 3). While approach (i) has been applied throughout this study, approach (ii) is implemented here for solubility calculations of all five additives. Subsequently, the results obtained from both approaches are compared. Representative results for BHT are shown in Fig. 12; corresponding plots for the other additives, which exhibit qualitatively similar behavior, are provided in the SI (Fig. S21). The relative differences between the solubilities predicted by the two approximations (RDiff%) and coefficients of variation (CV) are summarized in Table 13.

**Table 13:** Average relative difference (RDiff%) and coefficient of variation (CV) between COSMO-SAC solubility predictions using two different approximations for Δ*C*_p,fus_: neglecting the heat capacity term (Δ*C*_p,fus_ = 0) *vs.* estimating it as Δ*C*_p,fus_ = Δ*S*_fus_

	RDiff%	CV
BHT	11.3	0.32
ODP	10.2	0.33
TBP	232	0.21
NPH	108	0.86
PPB	24.0	0.22

Incorporating the heat capacity term leads to a systematic shift towards higher predicted solubilities across all studied additives. This can be rationalized by noting that Δ*S*_fus_ is positive in all cases, which causes the heat capacity integrals in eqn (2) to reduce the value of Δ*G*_fus_, thereby increasing the predicted activity. Qualitatively, the two approximations yield similar qualitative solvent rankings, although minor reordering occurs for solvents with comparable affinities (for TBP, see SI, Fig. S21). From a quantitative perspective, however, the choice of Δ*C*_p,fus_ approximation can introduce substantial differences—average relative errors differ by more than a factor of two for TBP, with the effect more pronounced for high-melting compounds. Nevertheless, since the primary focus of this study is qualitative solvent ranking rather than quantitative accuracy, we conclude the choice of Δ*C*_p,fus_ approximation appears to be of secondary importance for the intended screening application.

### 3.3 Solvent screening beyond available experimental data

So far, we have only considered additive–solvent systems for which experimental solubility data are available. For some additives, however, the list of solvents dictated by the experimental studies involves only one or a few solvent classes, as in the case for PPB.^61^ Although such solvent selections can be assumed to be relevant from a practical perspective, they may be considered somewhat narrow. In this section, we therefore provide COSMO-SAC solvent screening for systems beyond experimental data availability, *i.e.*, beyond the limits of the additive–solvent systems considered in this work so far.

This exercise is divided into two parts. In the first part, we extend the list of solvents for the five additives considered so far, thereby providing broader chemical diversity with respect to the solvent space. Our extended solvent dataset consists of 36 solvents selected to maximize chemical diversity while encompassing solvents commonly studied in solvent-based recycling processes.^2,7^ The set spans the full polarity range and enables comparison between conventional industrial standards and “green” alternatives.

**Fig. 13:**
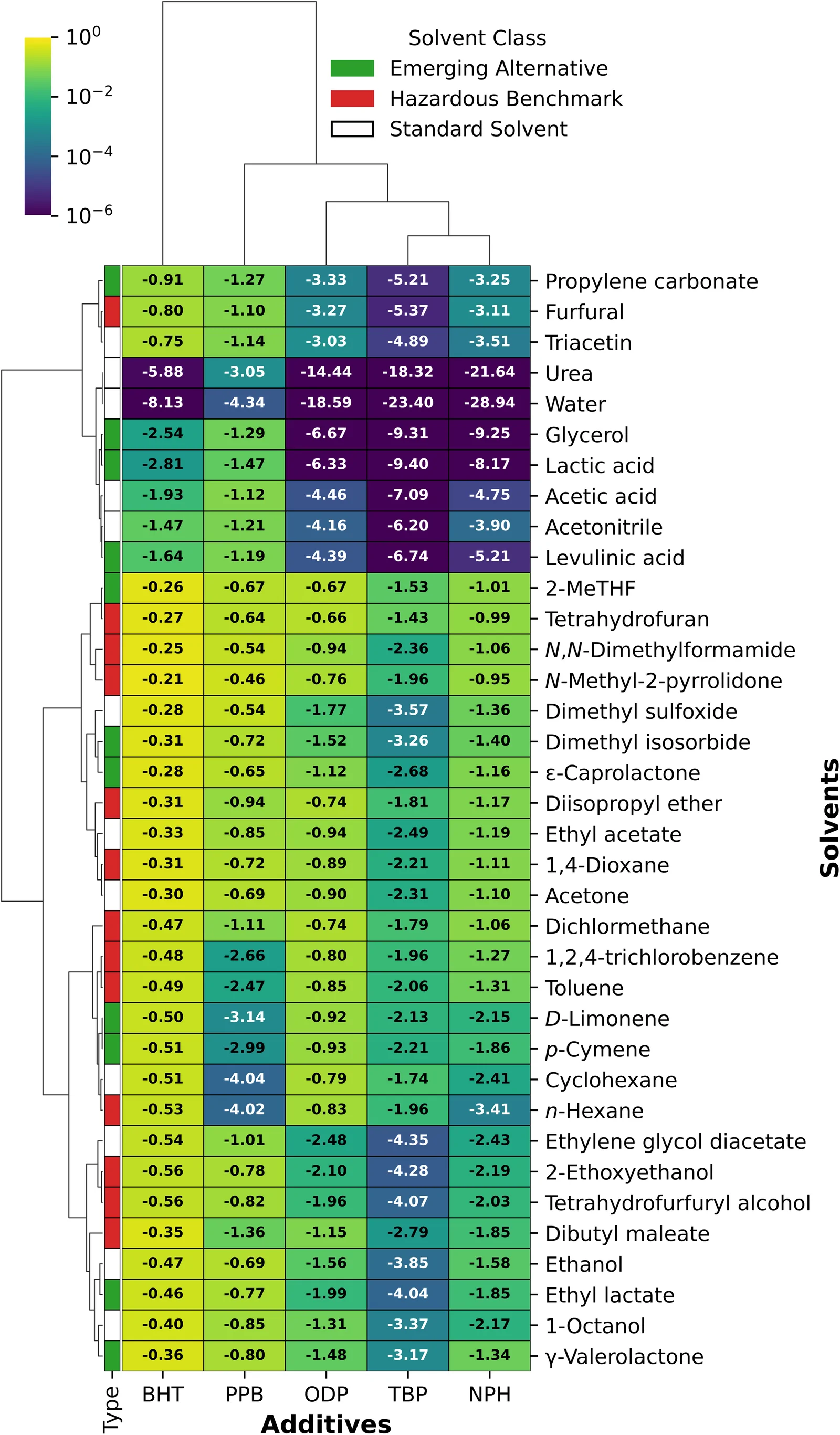
COSMO-SAC solvent screening of an extended set of solvents for the five investigated additives based on equilibrium solubilities *x*^SLE^_a_ at 293 K (values in cells represent log_10_ *x*^SLE^_a_).

The database includes selective solvents for key polymer classes: non-polar candidates for polyolefins (*e.g.*, cyclohexane, toluene), alongside terpene-based alternatives like *p*-cymene and d-limonene. For polyethylene terephthalate (PET) and polystyrene (PS) dissolution, polar solvents such as *N*-methyl-2-pyrrolidone (NMP) and tetrahydrofuran (THF) are included together with their green alternatives (*e.g.*, γ-valerolactone, propylene carbonate, and ε-caprolactone). The selection also incorporates common antisolvents (*e.g.*, water, ethanol, acetone) used for polymer precipitation. This design allows us to compare hazardous benchmarks with emerging bio-based alternatives considered in our parallel research.^38^ For the purpose of illustrating the screening capabilities across a diverse chemical space, the solvents in Fig. 13 and 15 were grouped into three categories: “Hazardous Benchmark” (representing common industrial solvents facing strict regulatory pressures, such as NMP, DMF, or toluene), “Standard Solvent” (widely used, conventional solvents like ethanol or acetone considered generally safe), and “Emerging Alternative” (usually bio-based and environmentally safe options such as γ-valerolactone or dimethyl isosorbide). In the specific context of solvent-based polymer recycling, the absolute environmental profile of a solvent cannot be evaluated in isolation. Ultimately, the true sustainability of a chosen solvent must be determined through rigorous life-cycle and techno-economic analyses of the entire process, including solvent recovery efficiency, energy demands of the separation steps, and specific product safety constraints.^3,5^

Calculated solubilities for the additives (BHT, ODP, TBP, NPH, and PPB) are presented as clustermap in Fig. 13. Qualitatively, the polar aprotic solvents are identified as the most effective group for the investigated additives, additionally, their solubility profile is quite similar with only PPB exhibiting notably different behavior—low affinity to non-polar and chlorinated solvents.

**Fig. 14:**
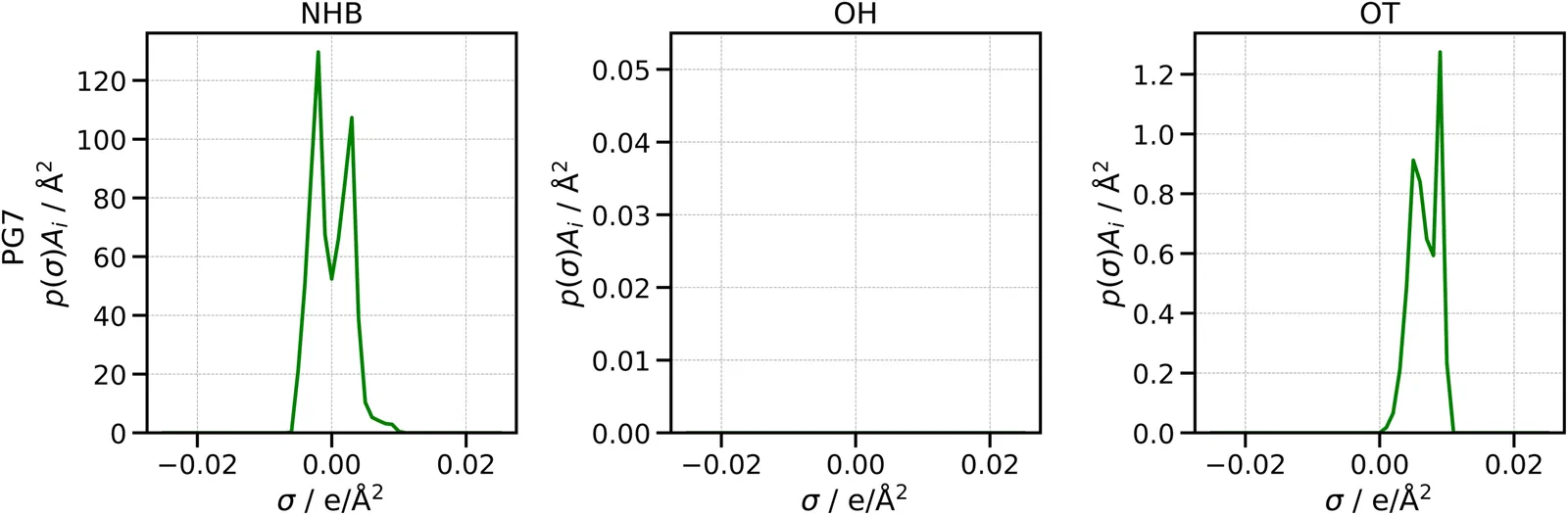
The *σ*-profile of Pigment Green 7 (PG7). The initial 3D structure of PG7 was, due to its complexity, taken from the crystal structure in CSD (refcode “UZEMIY01”).

**Fig. 15:**
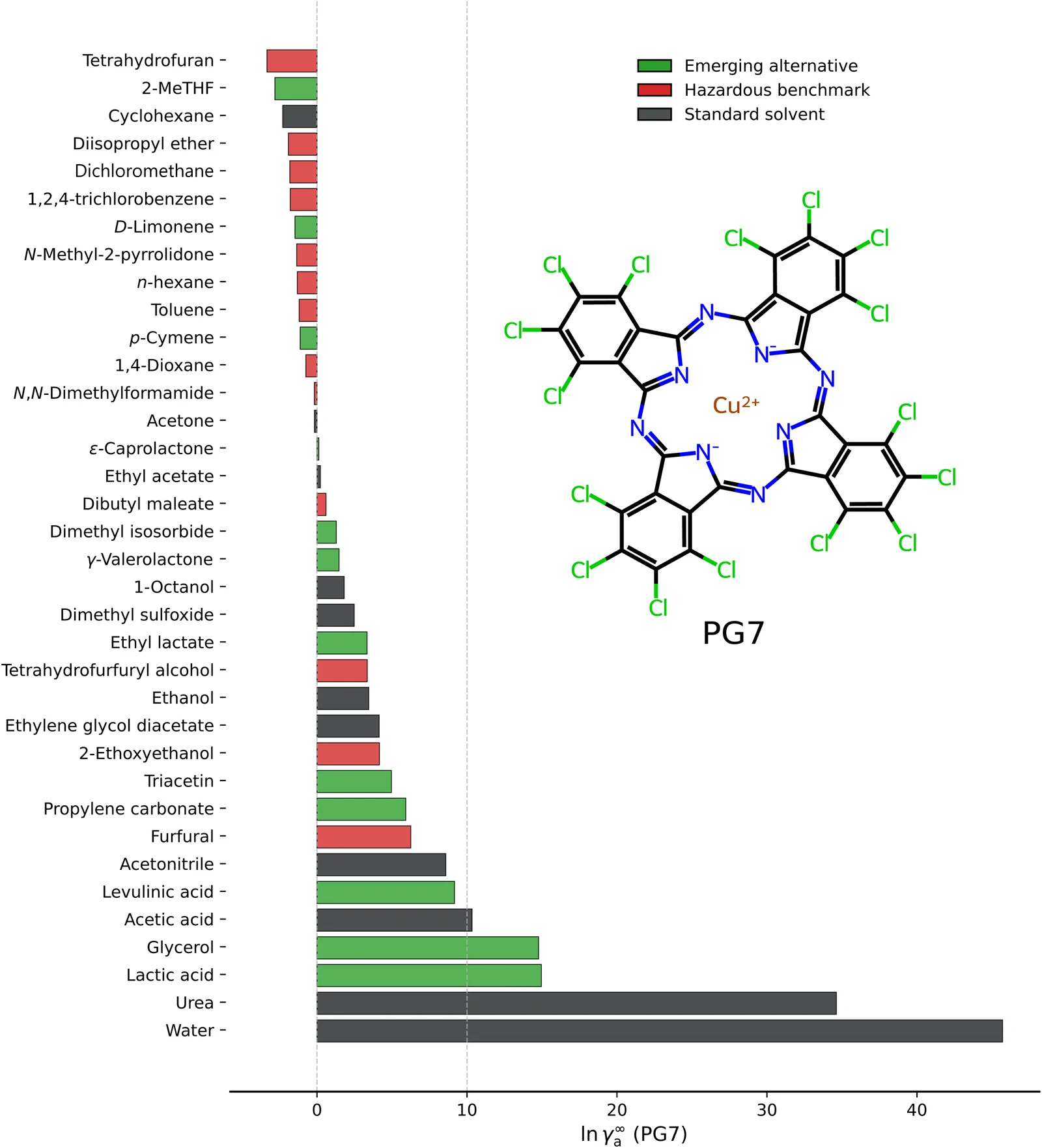
COSMO-SAC screening of solvents for Pigment Green 7 (PG7) based on infinite dilution activity coefficients ln *γ*^∞^_a_ at 293 K (solvent efficiency increases from bottom to top).

In the second part of this exercise, we examine a completely new additive—the pigment PG7—a coordination complex, structure of which is shown in Fig. 15. Due to the unavailability of its fusion data, the solvent screening is performed using ln *γ*^∞^_a_. The *σ*-profile of PG7 (Fig. 14) was calculated using the “supermolecule” ion-pair approach, in which both counterions are present as a pair in a specified optimized configuration, rather than being treated as two separate ions.^98,99^ In this approach, charge compensation between counterions, together with possible steric shielding and charge-transfer effects, typically leads to *σ*-profiles with less pronounced opposite-charge hot-spot peaks and, consequently, slightly reduced overall polarity compared to separate ions, as seen, *e.g.*, for ionic liquids.^99^

The resulting solvent ranking is presented in Fig. 15. Although this descriptor does not provide any information about absolute solubility values (as discussed in ref. 100), it effectively quantifies solvent affinity, identifying potentially the best and worst solvent candidates for PG7. The screening identifies water and glycerol as poor solvents, whereas polar aprotic solvents, such as THF and DCM, are predicted to be among the most effective. Notably, the model also suggests that aliphatic solvents should perform relatively well. This prediction suggests that interactions between PG7 and solvents are strongly influenced by bulky chlorinated aromatic moieties, among other effects.

This exercise (with the conclusions on model sensitivities presented in Section 3.2) could be extended to systematically evaluate the solubility profile for many other additives. It may also guide experts from practice toward new efficient/green solvents that have not yet been explored in the context of solvent-based recycling.

## 4 Conclusions

The structural and behavioral complexity of polymer additives competes with that of, for example, active pharmaceutical ingredients, and necessitates the use of sophisticated computational approaches. In this work, we examined and demonstrated the efficacy, robustness, and limitations of the open-source COSMO-SAC framework for quick and qualified ranking of solvents for model polymer additives.

Among hundreds of polymer additives, we primarily focused on additives for which sufficient experimental solubility data were available to enable systematic validation. The COSMO-SAC model showed typical prediction errors of approximately 0.5 log units (in mole fraction solubility) for most solvent classes. Larger deviations were observed for alcohols and water. Most importantly, the model consistently identifies water as the poorest solvent for the considered additives.

This work also serves as a methodological guide. The same computational framework can be readily applied to evaluate the solubility of other polymer additives, or extended to handle even more complex and specific chemicals among, *e.g.*, pigments. A key advantage of the COSMO-SAC approach over group contribution methods lies in its universality: by relying on universal QM calculations rather than on pre-parametrized functional groups, the method can handle structurally “diverse” molecules including macrocycles, heterocycles, ionic species, and compounds containing “inorganic” atoms, which may often not be covered by parameter matrices of even established semiempirical methods.^26^ For completeness, it must be noted that the current SLE workflow is fundamentally inapplicable to macroscopic particulate materials or purely inorganic networks (such as carbon black or TiO_2_), as they are either poorly chemically defined or would violate the assumption that the substance maintains its chemical form upon dissolution. However, we can speculate that ln *γ*^∞^_a_ may be applicable as a solvent affinity descriptor for such materials, quantifying the “like seeks like” principle proposed by Hansen.^101^

When experimental fusion data are unavailable, the infinite-dilution activity coefficient was demonstrated to provide a viable alternative descriptor for solvent screening. For low-solubility additives, ln *γ*^∞^_a_ yields rankings closely matching those obtained from full SLE calculations, whereas for highly soluble compounds, ln *γ*_a_ evaluated at higher compositions may be more appropriate. This approach was successfully applied to extend the solvent screening beyond experimentally validated systems, encompassing a broader chemical space including both conventional and emerging “green” solvents. The extended screening identified polar aprotic solvents as the most effective group for most antioxidants, while also highlighting the potential of bio-based alternatives such as γ-valerolactone and propylene carbonate.

Regarding the temperature dependence of solubility, while COSMO-SAC exhibits systematic errors at individual temperatures (often more pronounced for certain solvent classes), predicted solubility curves may be shifted, leading to crossings at different temperatures than observed experimentally.

The sensitivity analysis of fusion properties revealed that, while the qualitative solvent rankings proved robust to different Δ*C*_p,fus_ approximations, the quantitative sensitivity suggests that Δ*C*_p,fus_ treatment deserves further systematic investigation.

For consistent predictions, we recommend using a uniform methodology for generating *σ*-profiles, ideally from a single source, or at minimum, maintaining consistency by using database profiles for solvents and in-house calculations for additives.

Finally, we emphasize that predicted rankings should be interpreted globally (*e.g.*, identifying solvent classes with the best, moderate, or worst efficiency for a given additive) rather than focusing on subtle local differences within a single solvent class. This global perspective aligns with the initial stages of process design, where rapidly narrowing down the vast chemical space to identify promising candidates serves as a strategic first step.^7,43^ Once a shortlist is established based on such qualitative or semi-quantitative screening, the final solvent selection must inevitably incorporate a rigorous evaluation of process engineering metrics. Even within a promising solvent class, factors such as boiling point, viscosity, cost, industrial availability, and comprehensive environmental, health, and safety (EHS) or life-cycle analyses (LCA) ultimately dictate the optimal choice for scalable solvent-based recycling technologies.^3^ Interested researchers from both academia and industry are invited to explore the potential of COSMO-SAC in solvent screening of polymer additives *via* the COSMOSol tool developed as part of this study.

## Author contributions

A. B. conceptualization, investigation, methodology, data curation, formal analysis, software (lead), visualization, writing – original draft. J. K. writing – review & editing, resources, supervision. M. K. conceptualization, investigation, methodology, data curation, formal analysis, software (supporting), validation, writing – original draft.

## Conflicts of interest

There are no conflicts to declare.

## Supplementary Material

RA-016-D6RA05129D-s001

RA-016-D6RA05129D-s002

## Data Availability

The COSMOSol Python toolset, along with the calculated sigma-profiles and optimized molecular geometries, is open access. The specific code version and data replicating the findings of this study are archived on Zenodo (https://doi.org/10.5281/zenodo.18789358). The latest development version is available on GitHub at https://github.com/AdamBouz/COSMOSol. Supplementary information (SI): *σ*-profiles of the additives, sensitivity analyses (molecular conformations, *σ*-profile sources, dispersion contribution), additional figures on solvent rankings and composition/temperature dependence, and tables of solvents and Apelblat parameters. See DOI: https://doi.org/10.1039/d6ra05129d.
