# Supplementary material for: COSMOSol: efficient solvent screening for polymer additives with open-source COSMO-SAC
Source: RSC Adv. 2026 Jul 21;16(38):42112–34. doi: 10.1039/d6ra05129d (PMC13386427; doi:10.1039/d6ra05129d)
Supplement: Supplementary file 1 [file RA-016-D6RA05129D-s002.pdf]

Supplementary Information (SI) for RSC Advances.
This journal is © The Royal Society of Chemistry 2026

Supporting Information

COSMOSol: Efficient Solvent Screening for Polymer Additives with Open-Source COSMO-SAC

Adam Bouz, Juraj Kosek, and Martin Klajmon

Faculty of Chemical Engineering, University of Chemistry and Technology, Prague, Technická 5, 166 28 Prague 6, Czechia

Email: adam.bouz@vscht.cz, martin.klajmon@vscht.cz

Contents

Section S1. Sigma Profiles of Investigated Additives S-2
Section S2. Sensitivity to σ-Profiles and Molecular Structures S-3
Section S3. Influence of Dispersion Contribution S-7
Section S4. Additional Figures S-10
Section S4.1. Rankings S-10
Section S4.2. Composition Dependence S-12
Section S4.3. Temperature Dependence S-14
Section S4.4. Fusion Sensitivity S-16
Additional Tables S-17
Table S1: List of solvents and their physical properties S-17
Table S2: Apelblat equation parameters for antioxidant solubilities S-18
Table S3: Comparison of solubility predictions with and without dispersion term S-19
Table S4: Contributions to infinite dilution activity coefficient S-20

S1 Sigma profiles of the investigated additives

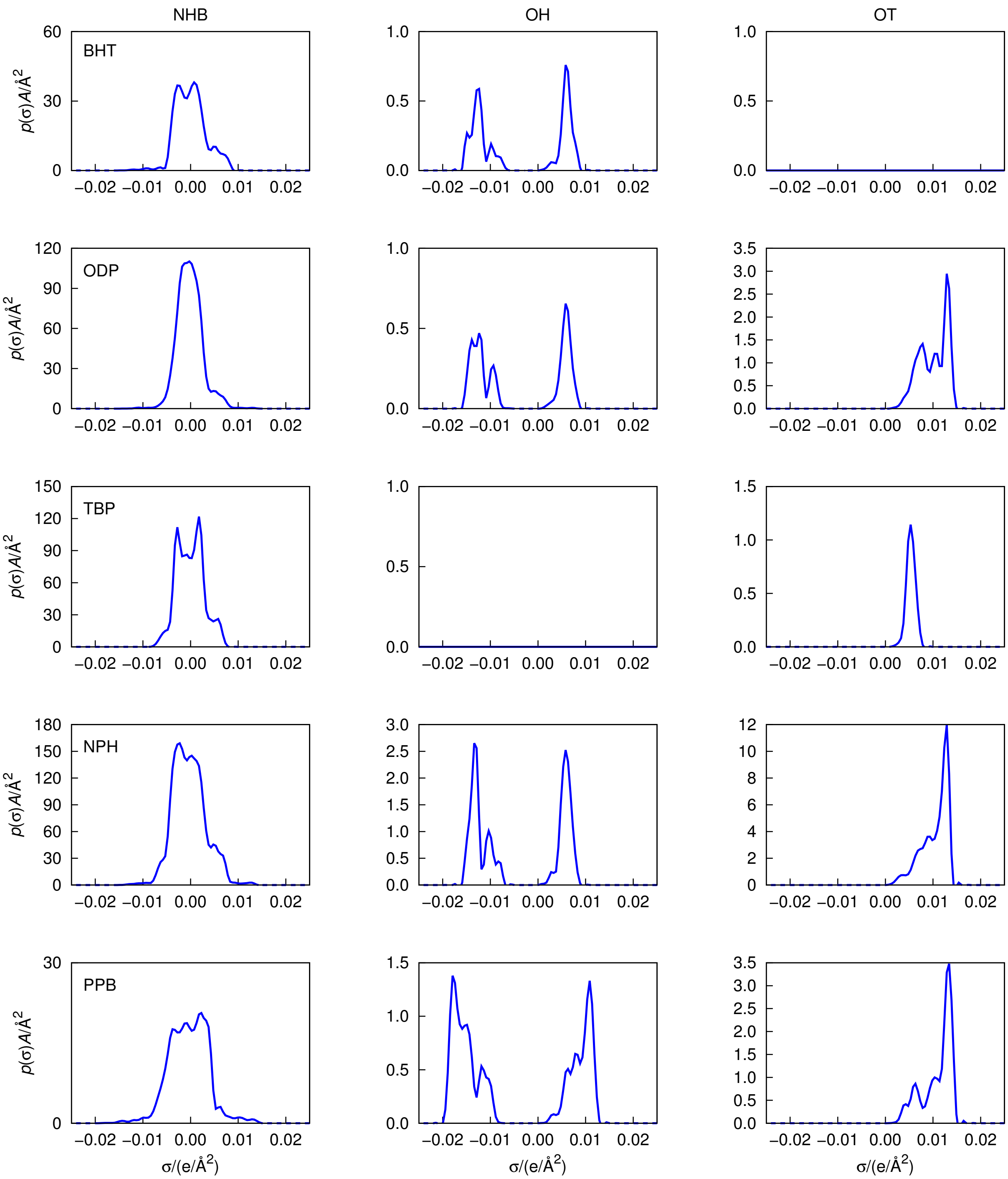

Figure S1: Sigma profiles of the investigated additives.

S2 Sensitivity to Different Molecular Structures and σ-Profiles

In this section, the sensitivity of the COSMO-SAC results to variations in σ-profiles of solutes, either due to different molecular conformations or σ-profile sources is examined, using PPB as an example.

Previous studies[S1–S4] have shown that the most significant effects can be expected for substances exhibiting intramolecular HB. However, none of the additives considered in this work possesses this feature, making a dramatic effect of conformation on COSMO-SAC results less likely.

To assess the sensitivity to σ-profile variations, six different σ-profiles for PPB were compared:

- the reference σ-profile determined in this work and used in Section 3.1.5 of the main manuscript (denoted "ref");
- a profile derived from the crystal structure of PPB present in the CSD[S5] (CSD refcode "DUPKAB");
- profiles of two other conformers adopted from the PubChem database[S6,S7] (denoted "PubChem_conf4" and "PubChem_conf8");
- the σ-profile from the UD database[S8,S9] (denoted "UD");
- a profile of the reference geometry recalculated with ORCA[S10] at the DFT/BP86/TZVP/C-PCM level instead of Gaussian (denoted "ref_ORCA").

The σ-profiles of all variants but "UD" and "ref_ORCA" were calculated using the same protocol as "ref", *i.e.*, at the DFT/BP86/TZVP/C-PCM level of theory in Gaussian. Figure S2 shows structures of the considered conformers and additional information, while Figure S3 illustrates the differences among the corresponding σ-profiles. They arise from variations

in molecular geometry (this applies mainly to "ref", "DUPKAB", and the two PubChem conformers) and different QM computational protocols (variant "ref_ORCA" and "UD", because the UD database was developed using a different QM software and data processing tools). The "ref_ORCA" variant uses identical geometry and comparable level of theory (DFT/BP86/TZVP/C-PCM) as "ref", but was computed with ORCA[S10] rather than Gaussian, isolating the effect of the QM code itself. To enable this comparison, the `to_sigma.py` tool of the open-source COSMO-SAC implementation[S9] was extended to parse ORCA C-PCM output files with segment charges.

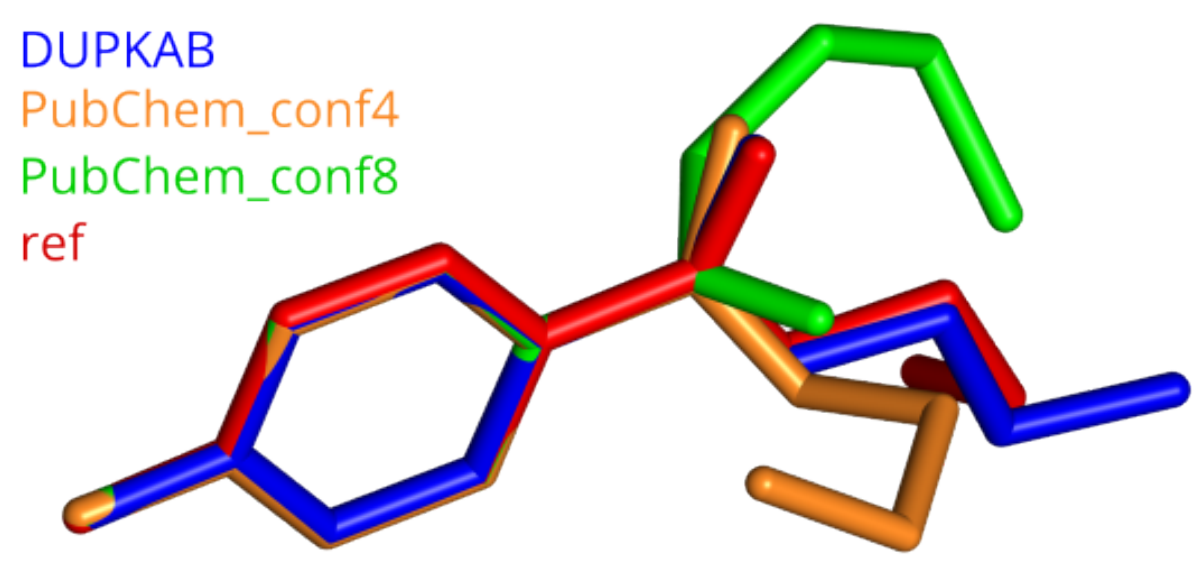

Figure S2: The different conformers of PPB. Note the minor difference between the "ref" and the planar "DUPKAB" structures regarding the terminal methyl unit. The PubChem conformers exhibit greater structural variation; for instance, in "PubChem_conf8", the planes of the phenolic and ester groups adopt an angle of ca. 90°.

Figure S3 shows that, although the compared σ-profiles are not identical, they are quite similar both quantitatively and qualitatively. As a result, they translate into relatively minor changes in the predicted solubilities (see Figure S4). Specifically, while the mutual deviations between solubilities obtained using the four σ-profiles determined in this work are relatively small and do not exceed a mole fraction of 0.05, those between "UD" and the bundle of our four σ-profiles are, in most cases, the largest, though still not exceeding an x_a value of 0.1. This suggests that the origin of σ-profile may play a more significant role than the specific conformer within a consistent computational protocol. Interestingly, when compared to experimental data, the "UD" and "ref_ORCA" versions achieve the largest error. More importantly, the qualitative solvent rankings remain largely preserved across all five σ-profile variants. This observation suggests that for screening purposes, COSMO-SAC

predictions are reasonably robust with respect to moderate variations in input σ-profiles, provided that (i) a consistent methodology is applied across all compounds in the study and (ii) the compounds do not possess significant intra-HB features.

An a priori unexpected finding emerges from the "ref_ORCA" comparison: although this variant shares both the geometry and the nominal level of theory (DFT/BP86/TZVP/C-PCM) with "ref", switching only the QM program from Gaussian to ORCA produces deviations in the predicted solubilities of a magnitude comparable to those caused by adopting an entirely different conformer. In other words, the choice of QM code is not a neutral implementation detail—differences in the underlying numerical algorithms (basis set handling, C-PCM cavity construction, segment discretization, *etc.*) propagate into the σ-profile and, ultimately, into the predicted phase behavior. While the qualitative rankings remain robust (as discussed above), this observation has practical consequences—for the most consistent qualitative predictions, we strongly recommend generating all σ-profiles within a single, unified protocol.

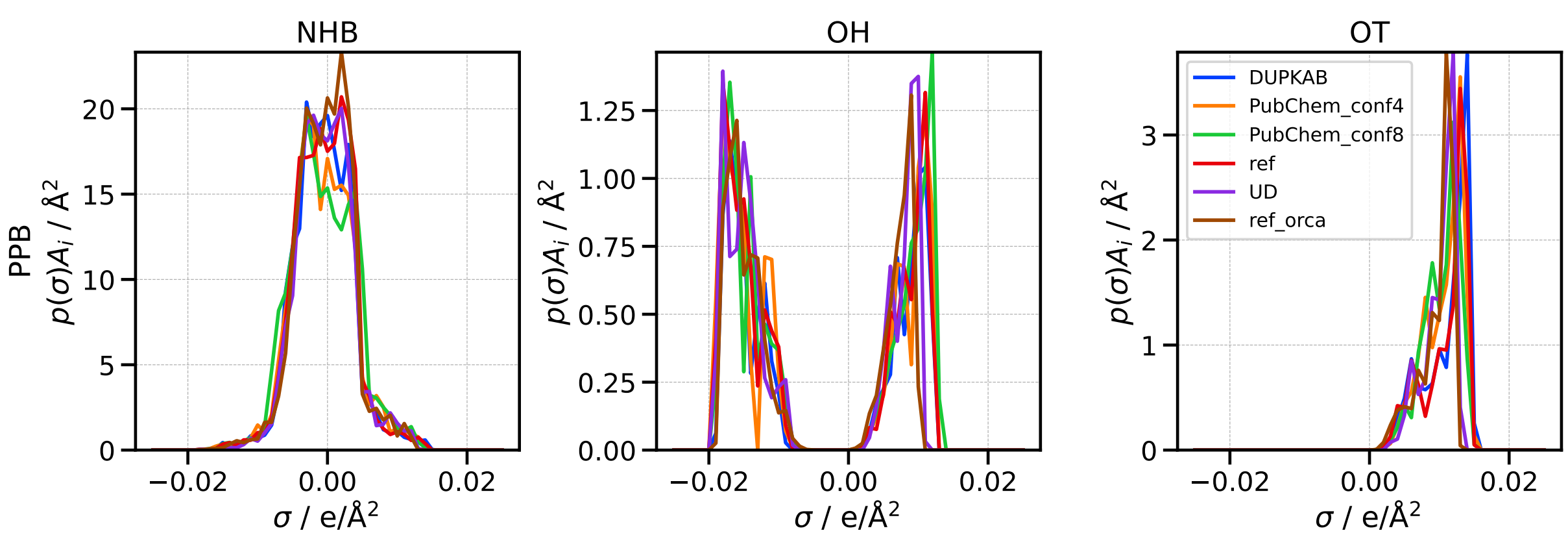

Figure S3: Comparison of the different σ-profiles for PPB.

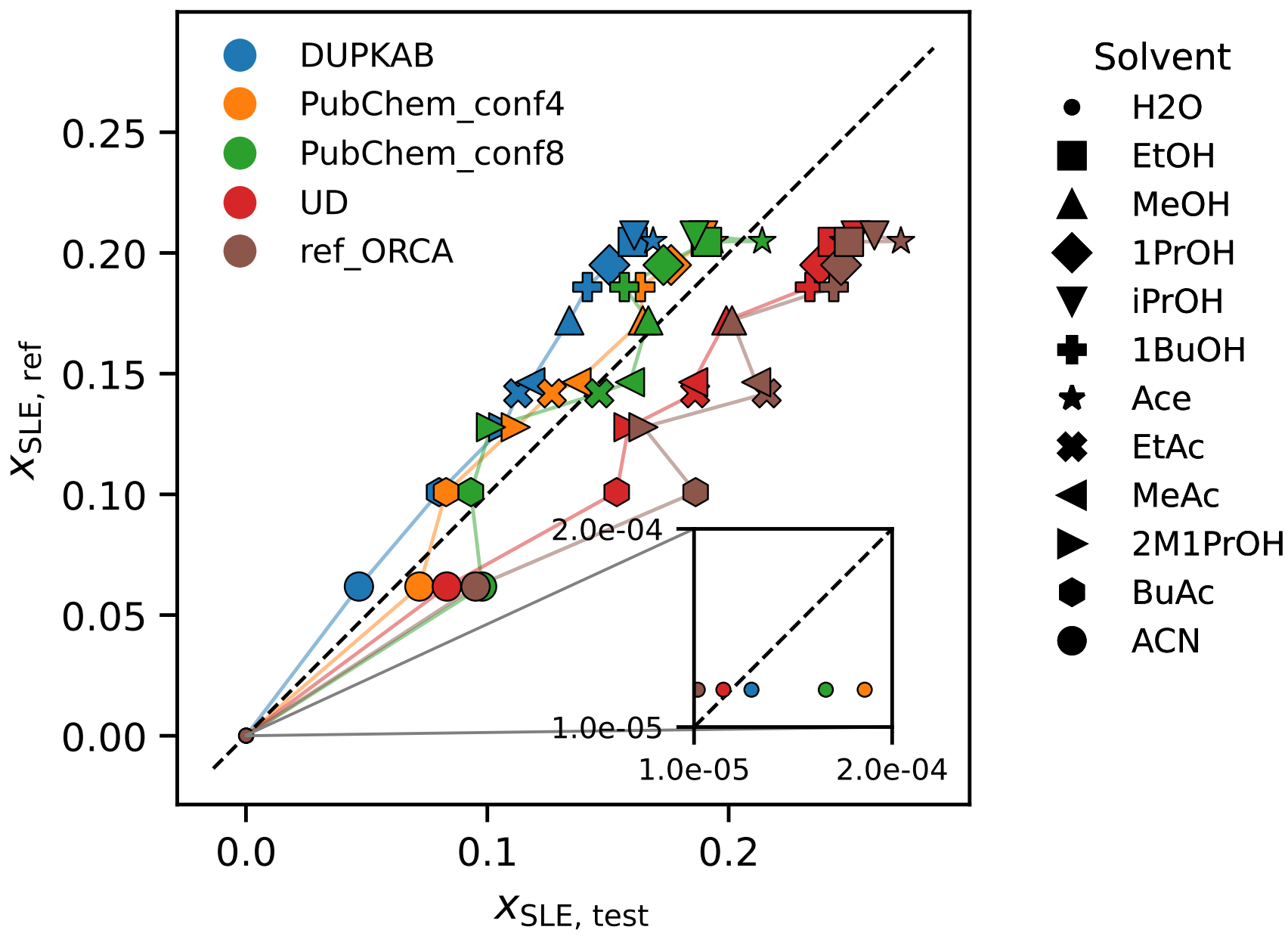

Figure S4: Parity plot comparing the solubility predictions for PPB obtained using different σ-profiles shown in Figure S3.

S3 Influence of the Dispersion Contribution: COSMO-SAC-2010 vs COSMO-SAC-dsp

In this section, we explore whether the inclusion of the dispersive term in COSMO-SAC improves the qualitative or quantitative results for additive–solvent systems in any way. This is mainly motivated by the fact that some polymer additives (not necessarily those considered in this study) and solvents (*e.g.*, chloroform) are highly halogenated, for which COSMO-SAC-dsp has been reported to yield improved results compared to COSMO-SAC-2010,[S11] albeit with a more pronounced effect reported for fluorinated than chlorinated compounds.

The dispersion term introduced by Hsieh *et al.*[S11] is a one-parameter Margules equation, where the Margules constant is derived from molecular dispersion parameters calculated using element-specific contributions. A similar approach based on the introduction of element-specific constants was proposed in the original COSMO-RS model by A. Klamt[S12–S14] (implemented in the COSMO*therm* software) and it is generally acknowledged that the nonideality arising from dispersion interactions is not *fully* captured by the residual contribution. Although Hsieh reports that the model significantly improves the accuracy of $\ln \gamma_i^\infty$ (by 33% on the testing dataset, and even more for fluorine compounds), the element-specific constants were obtained through fitting to experimental vapor–liquid equilibrium (VLE) and $\ln \gamma_i^\infty$ data. While the resulting Lennard-Jones energy parameters are within reasonable physical limits, this improvement comes at the cost of (i) heavier parametrization and (ii) reduced generality of the model, compared to COSMO-SAC-2010. We considered it important or at least interesting (for the parametrized additives and solvents) to compare the performance of SLE predictions with and without the dispersion term.

The results for all validated additives are summarized in Table S3. Even though the overall predictions for the entire dataset show a slight improvement, the enhancement with respect to experimental solubilities is not particularly significant. Specifically, solubility predictions in chlorinated solvents (chloroform, dichloromethane) are virtually unaffected

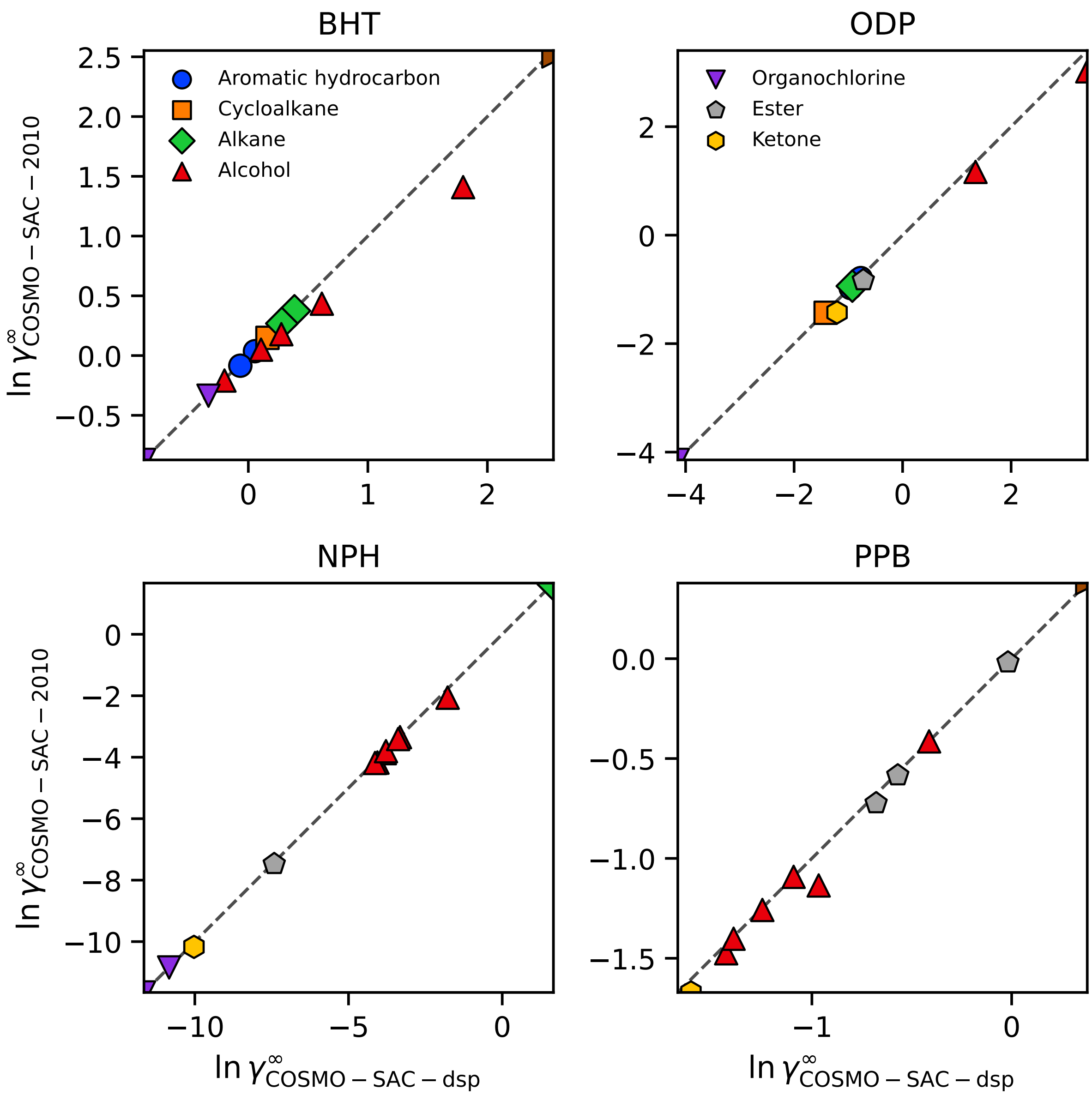

Figure S5: Comparison of $\ln \gamma^{\infty}$ calculated by two COSMO-SAC configurations: (COSMO-SAC-2010): SG combinatorial + residual contribution and (COSMO-SAC-dsp[S11]): SG combinatorial + residual + dispersive contribution

by the dispersion term. Notably, *there are no changes in the ranking.* This is generally consistent with previous observations, albeit for different systems.[S3]

Since $\ln \gamma_i^{\infty}$ values typically represent extreme values of the activity coefficient, we decided to investigate the influence of individual contributions on them. As shown in Table S4 and

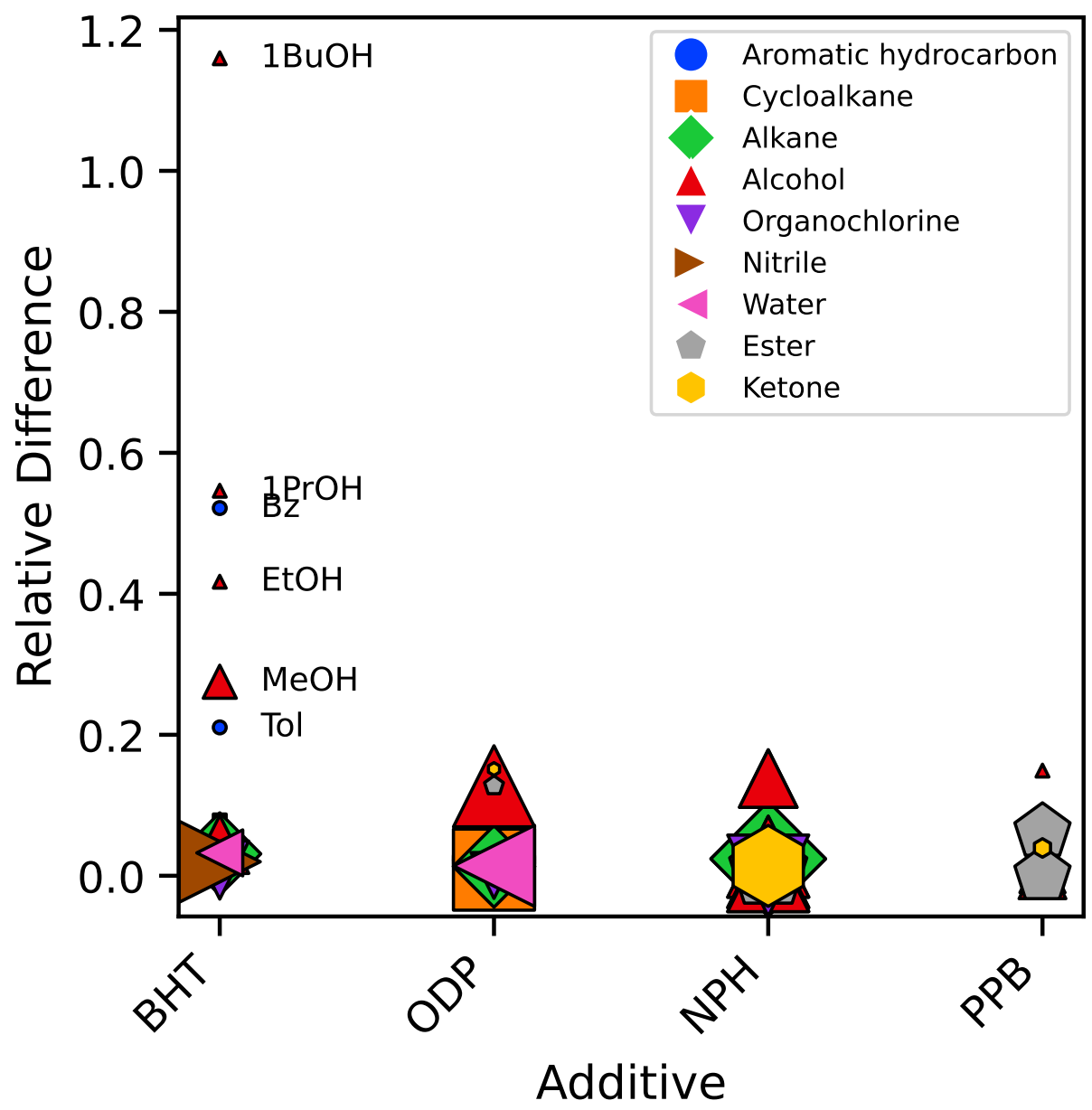

Figure S6: Relative influence ($\ln \gamma^{\infty}_{\text{disp}} / \ln \gamma^{\infty}_{\text{COSMO-SAC-2010}}$) of the dispersive contribution on the overall $\ln \gamma^{\infty}$. Markers are scaled by the value of $\ln \gamma^{\infty}_{\text{COSMO-SAC-2010}}$

visualized via a parity plot in Figure S5, the residual contribution dominates in most binary systems, while the dispersion term is only significant in systems where $\ln \gamma^{\infty}$ is close to zero, *i.e.*, for (nearly) ideal solutions, where the dispersion term is the only contributor to nonideality (with relative contributions shown by marker size in Figure S6). For our systems, the dispersion contribution ranges from 0 to (only) 0.6 ln units.

S4 Additional Figures

S4.1 Rankings

Comparison of solvent rankings for (BHT, ODP, TBP, NPH, and PPB) based on different COSMO-SAC descriptors versus experimental ranking: (left) equilibrium solubility x_a^{SLE}, (middle) infinite dilution activity coefficient $\ln \gamma_a^\infty$, (right) activity coefficient at $x_a = 0.3$.

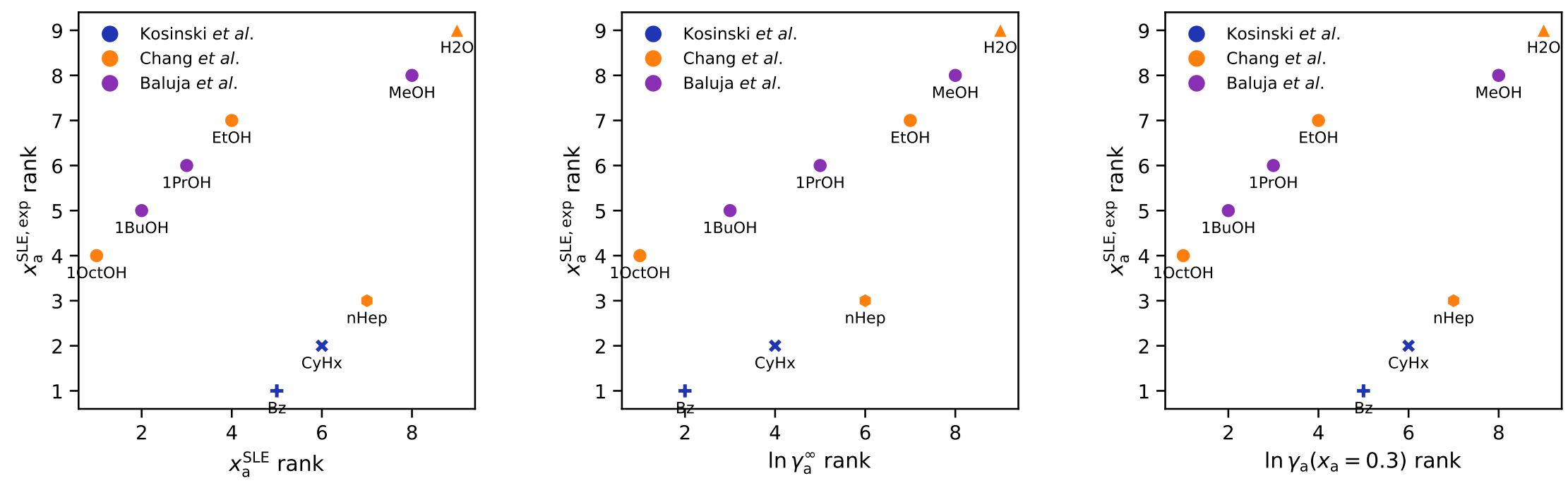

Figure S7: BHT

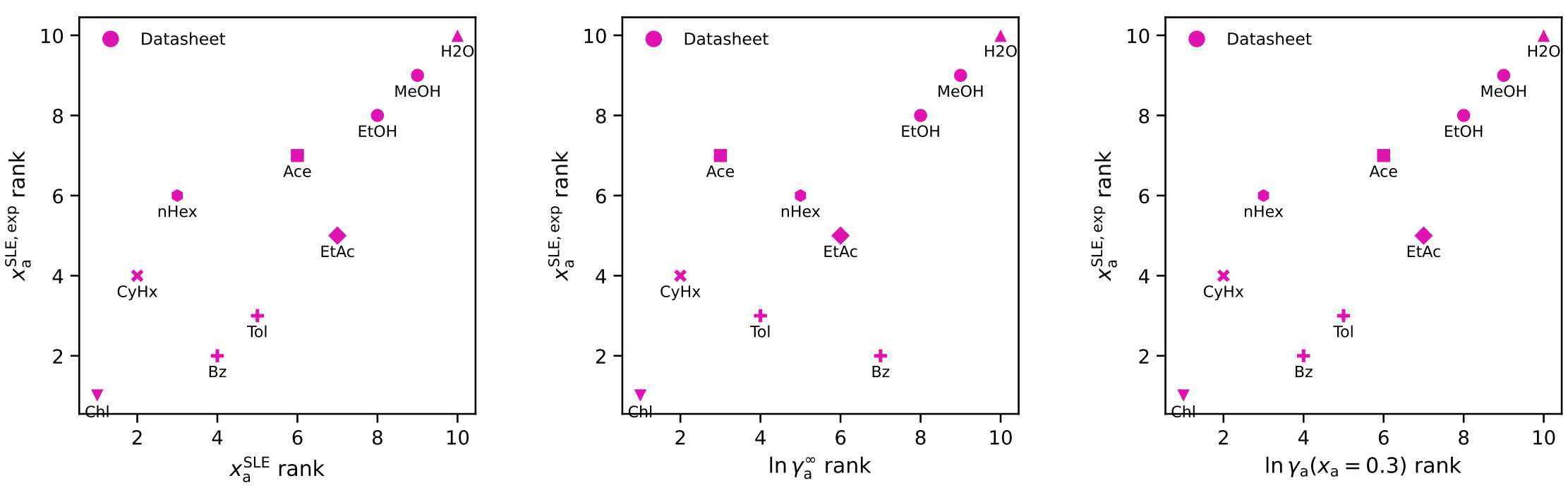

Figure S8: ODP

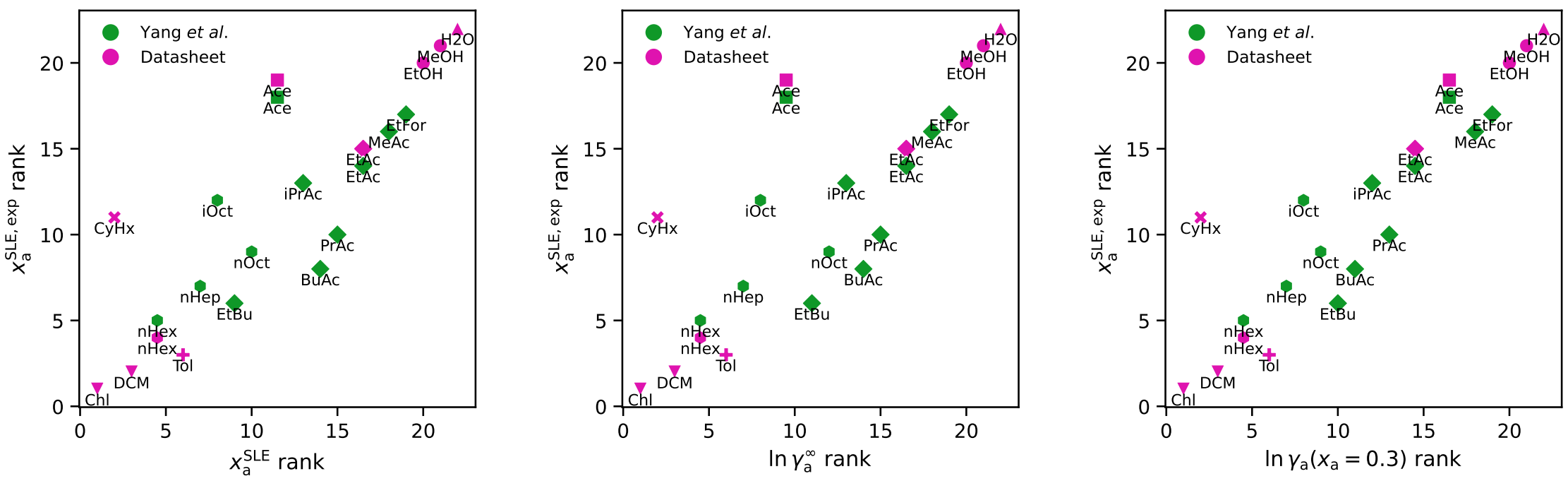

Figure S9: TBP

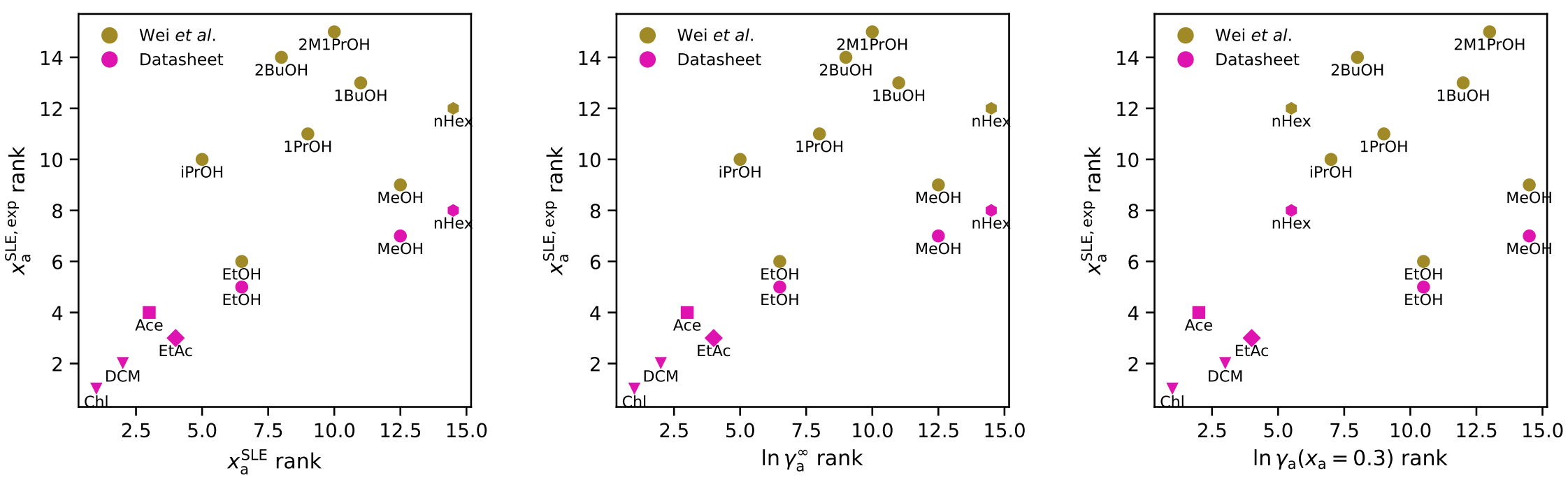

Figure S10: NPH

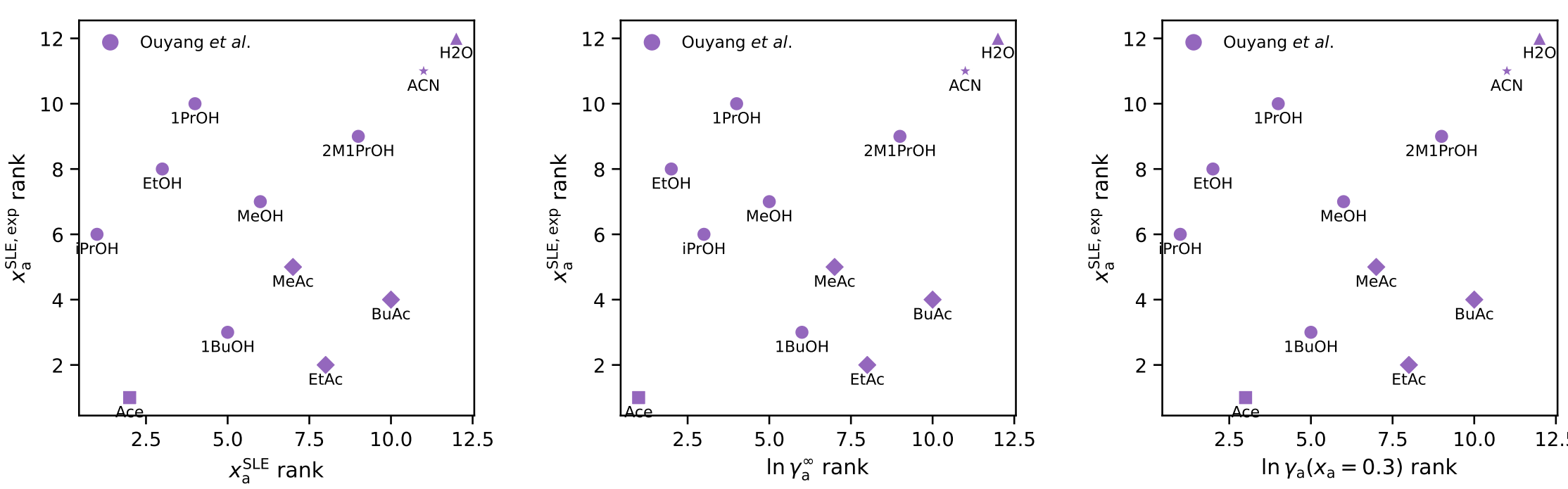

Figure S11: PPB

S4.2 Composition Dependence

The composition dependence of $\ln \gamma_a$ for (BHT, ODP, TBP, NPH, and PPB) in various solvents. The points represent $\ln \gamma_a$ values at infinite dilution ($x_a \rightarrow 0$), $x_a = 0.3$, and saturation (*i.e.*, equilibrium solubility).

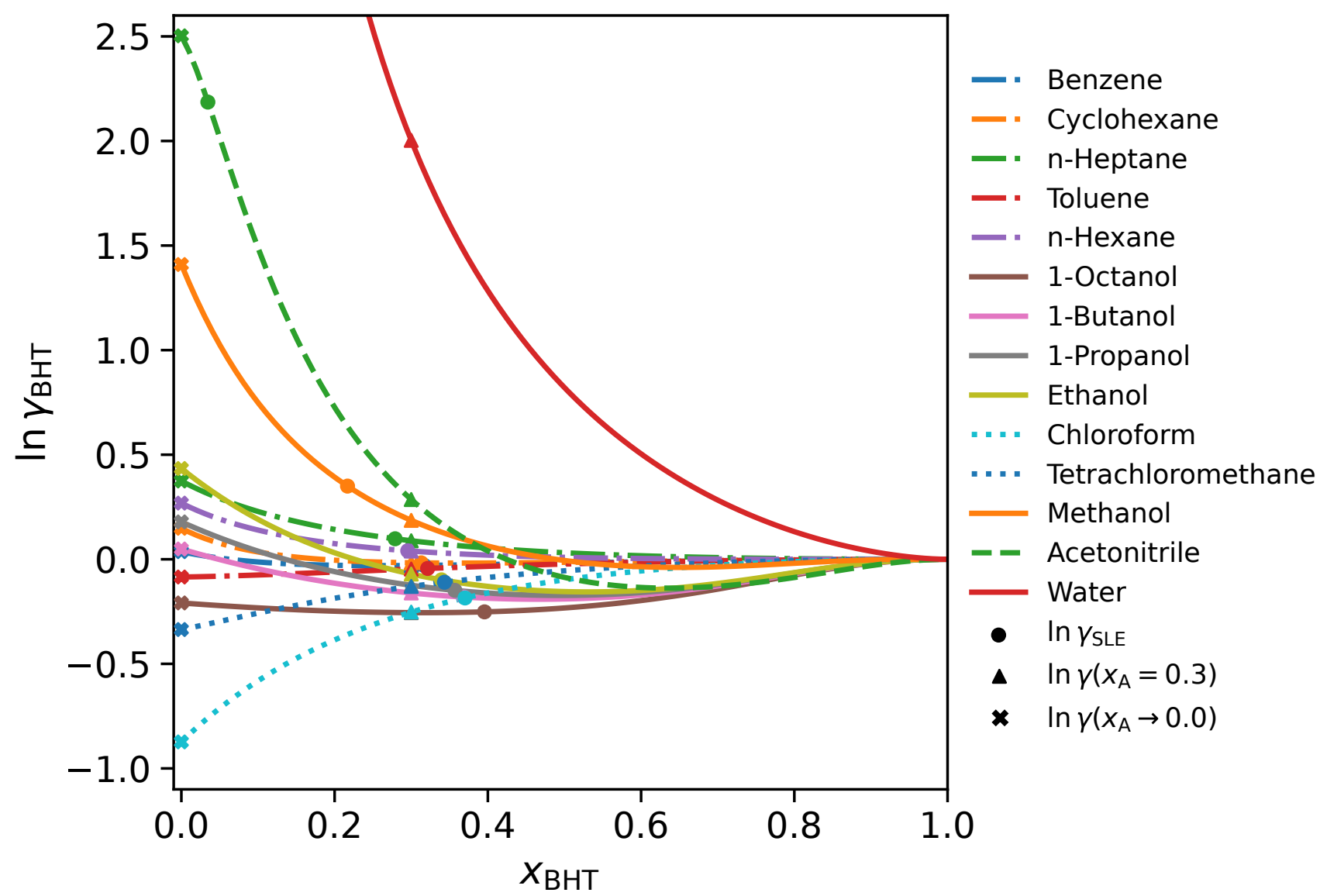

Figure S12: BHT

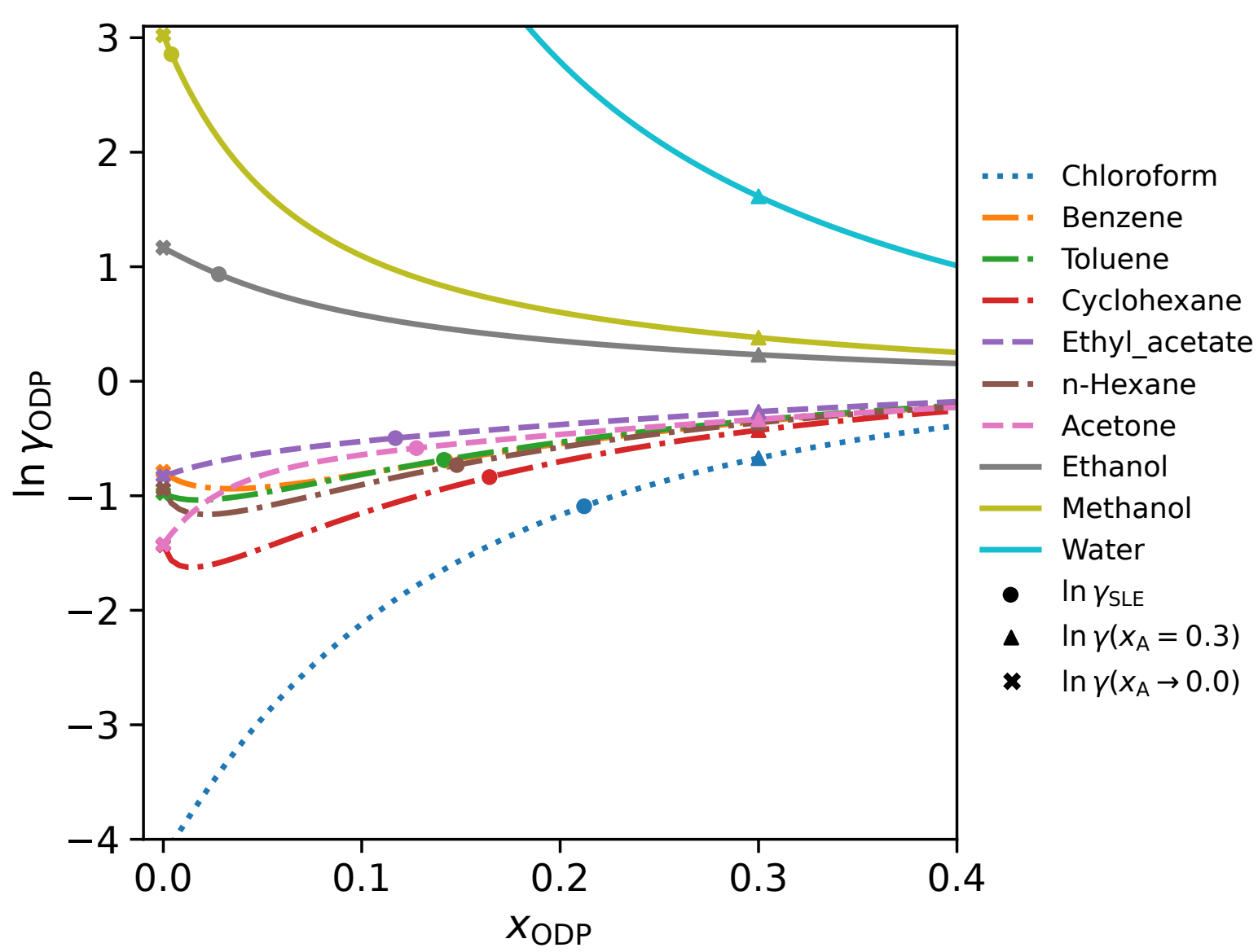

Figure S13: ODP

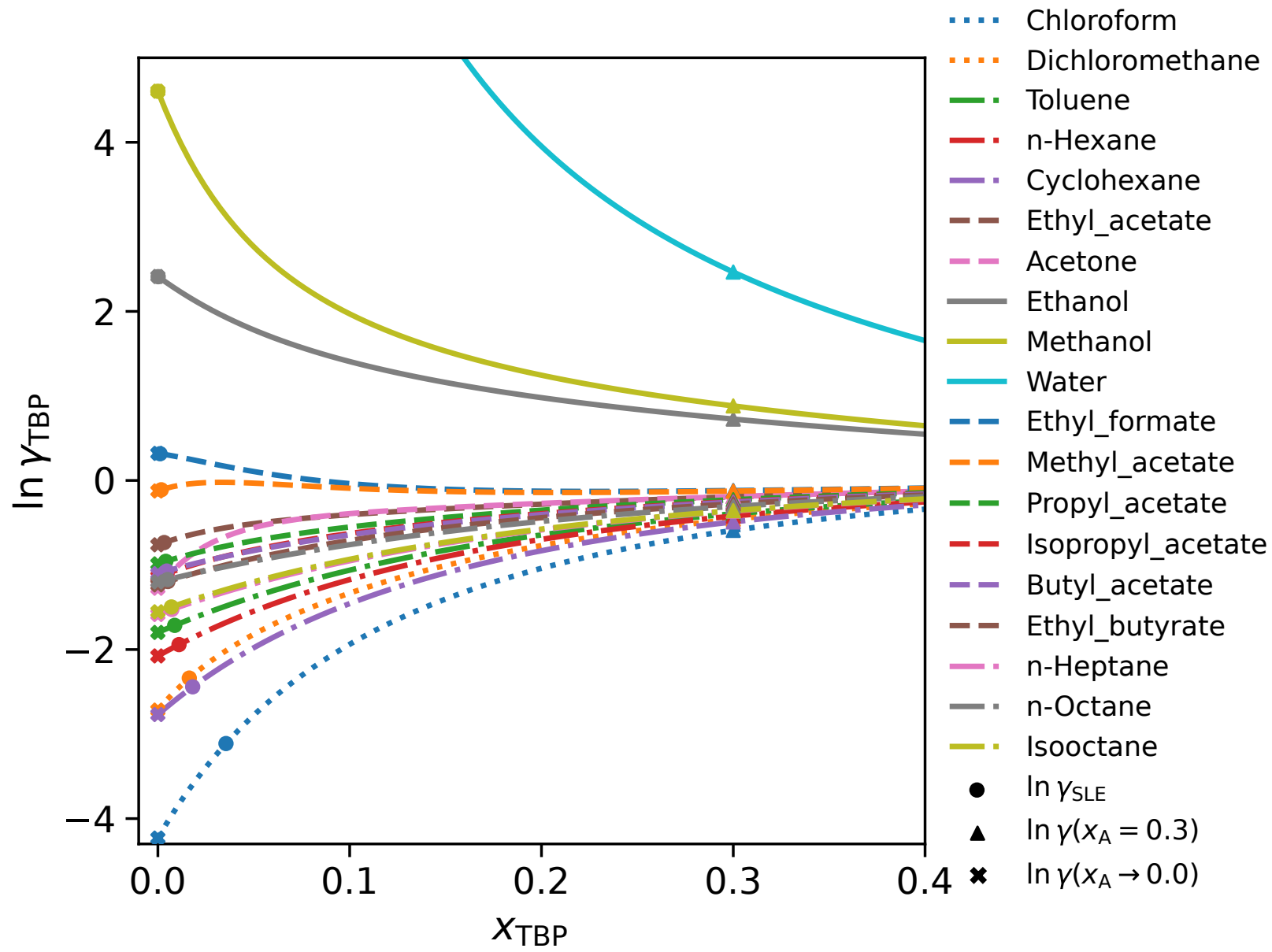

Figure S14: TBP

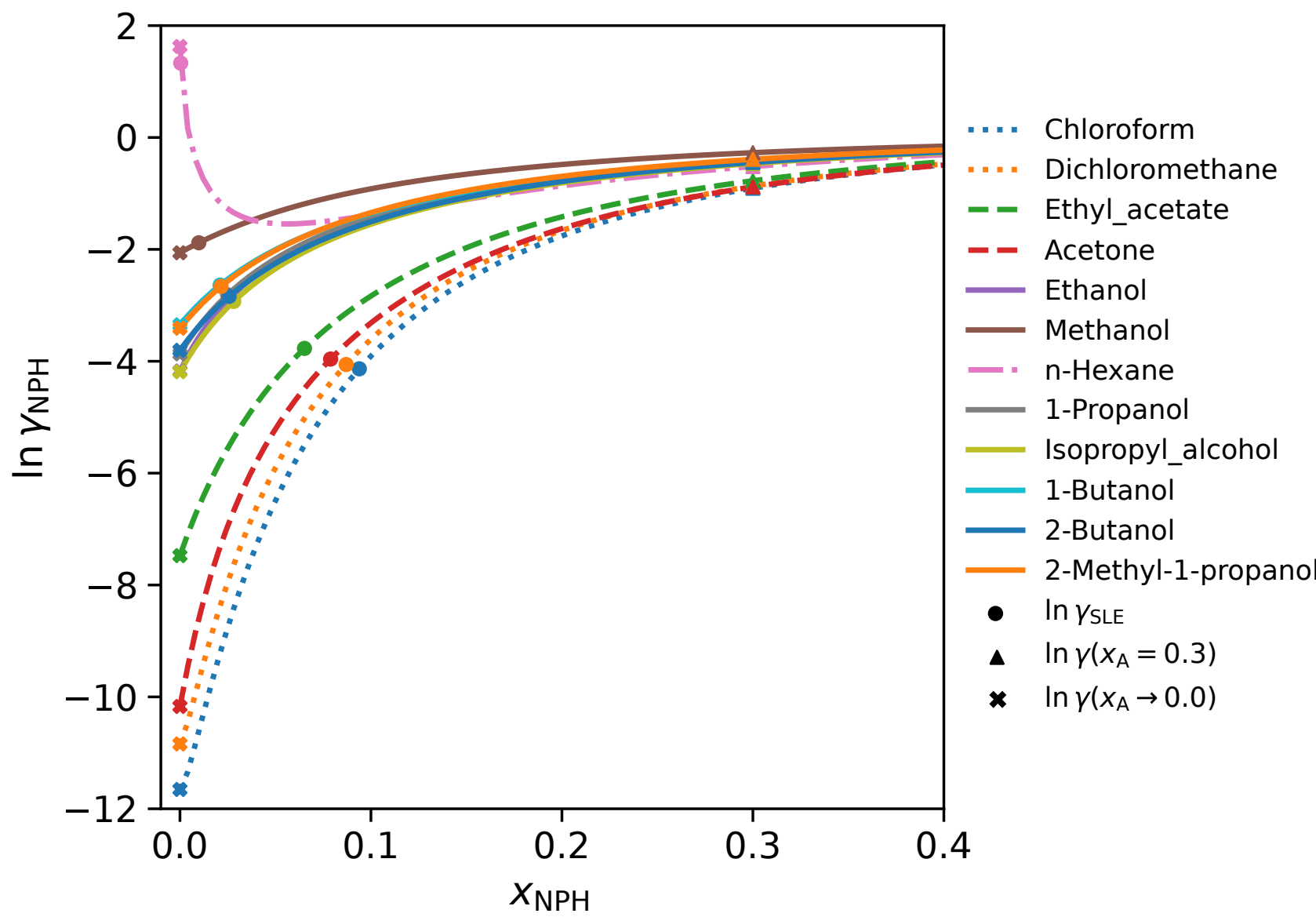

Figure S15: NPH

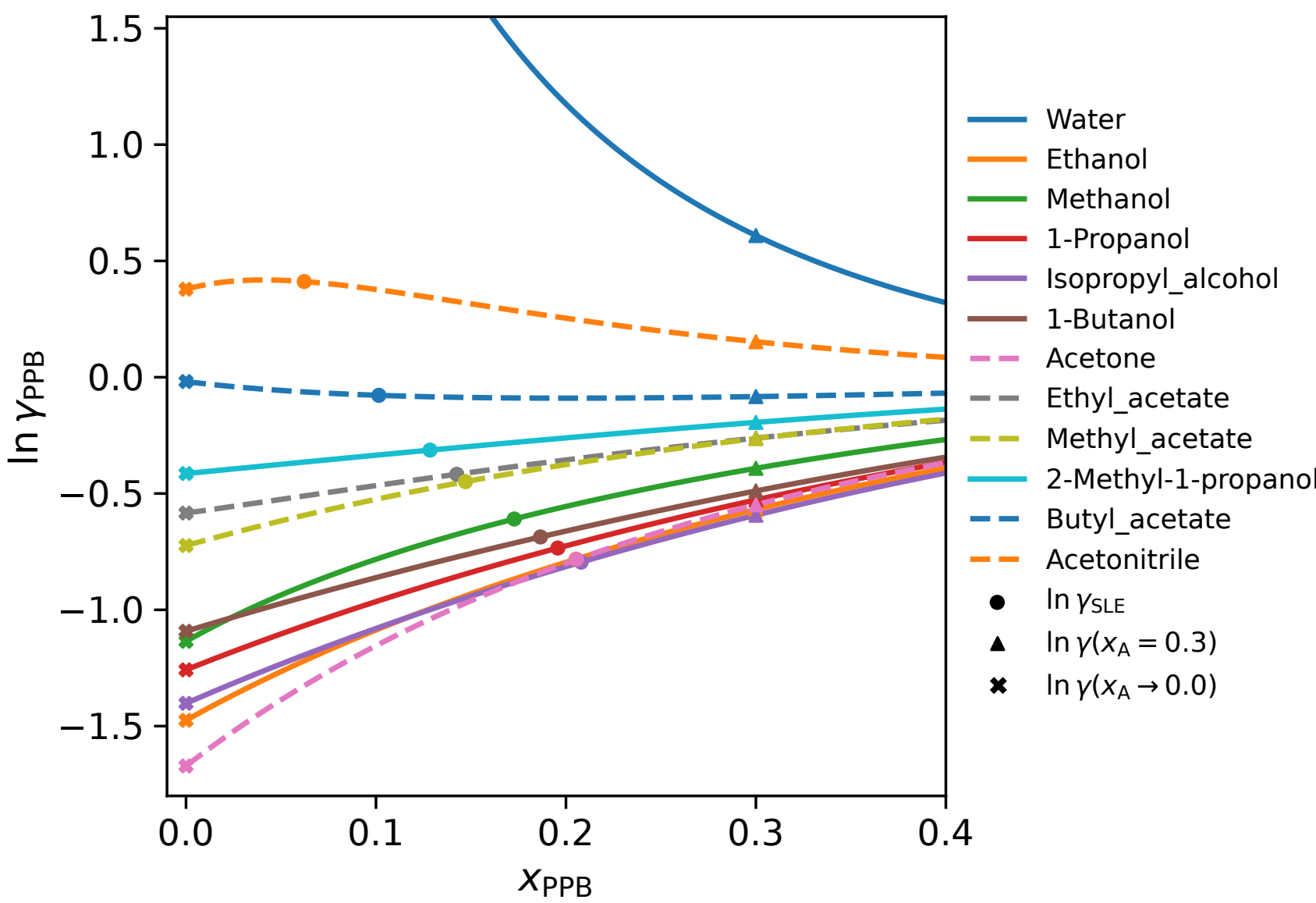

Figure S16: PPB

S4.3 Temperature Dependence

The temperature dependence of pseudo-experimental (dashed) and calculated (solid) solubilities of PPB, TBP and BHT in various solvents. "Pseudo" indicates that correlations based on the modified Apelblat equation were used to plot the T dependence.

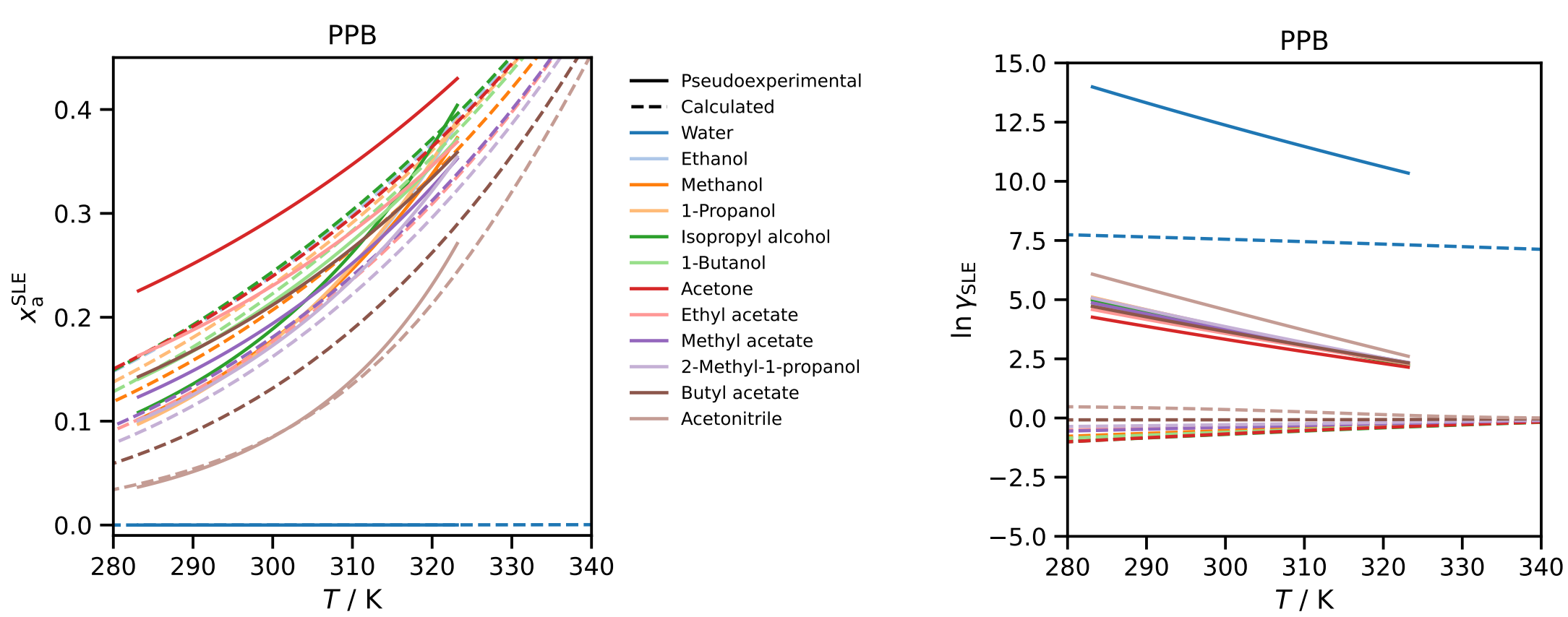

Figure S17: PPB

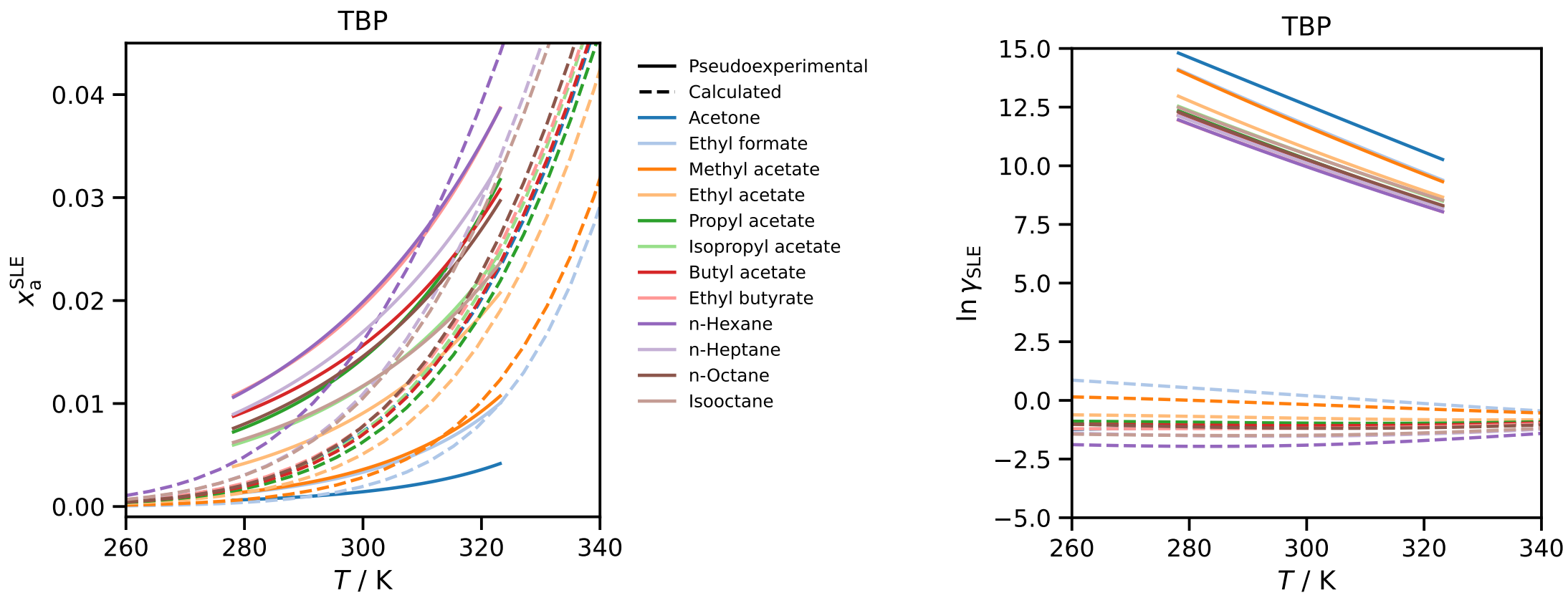

Figure S18: TBP

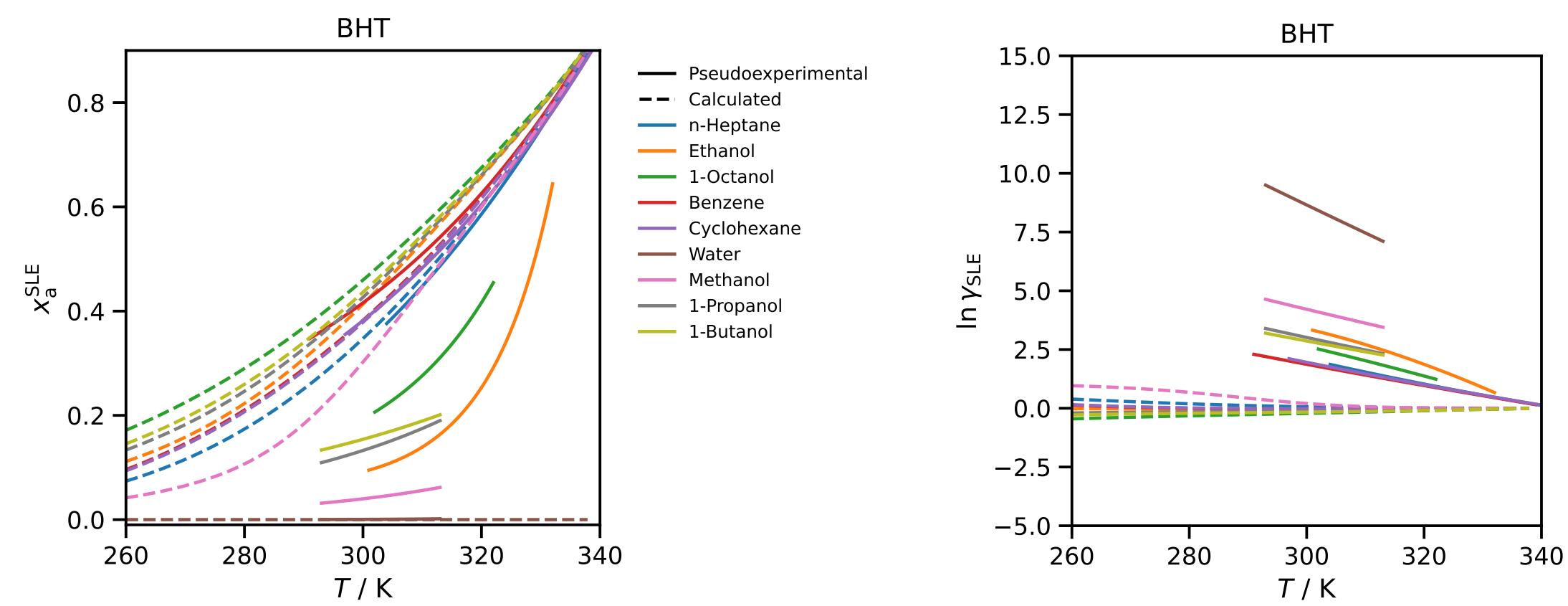

Figure S19: BHT

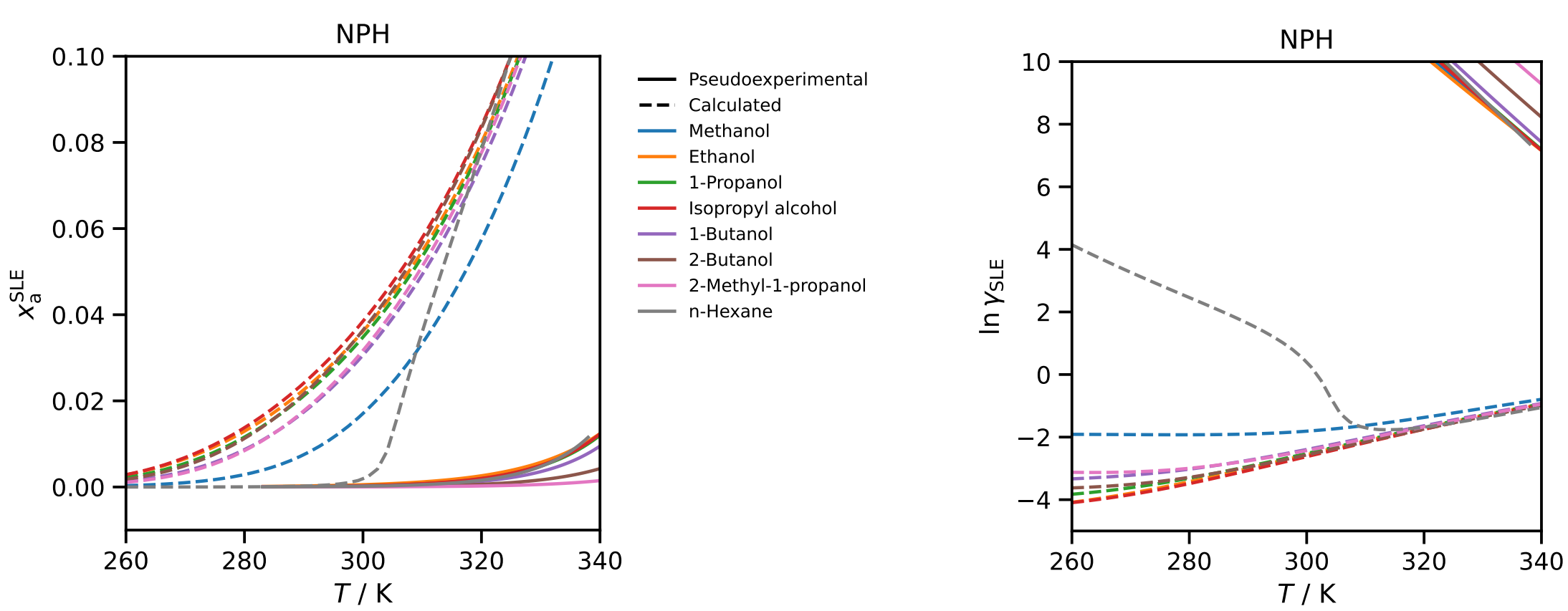

Figure S20: NPH

S4.4 Fusion Sensitivity

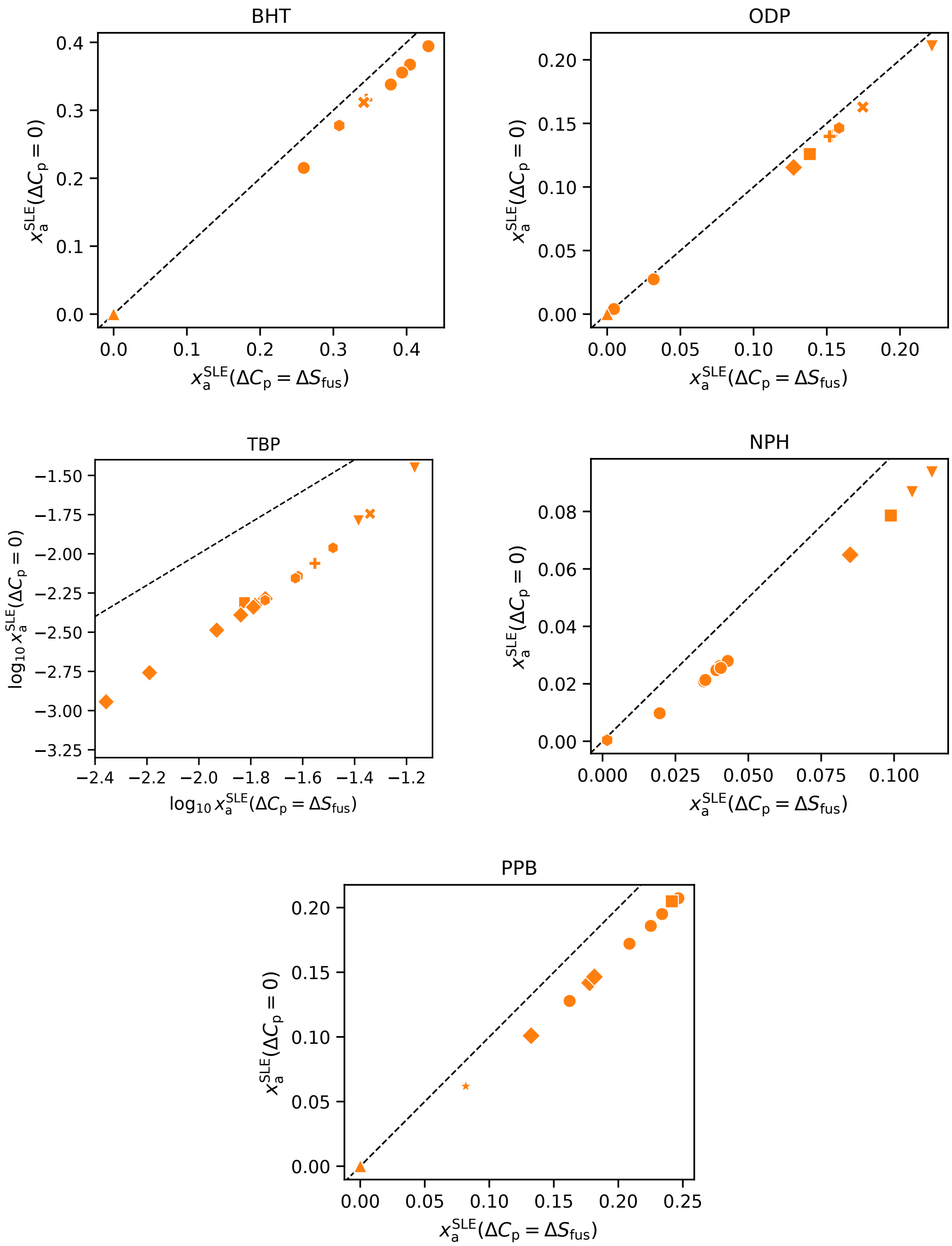

Figure S21: Sensitivity of solubility predictions to different approximations of $\Delta C_{p,\mathrm{fus}}$ for all validated antioxidants: parity plots comparing COSMO-SAC predictions using $\Delta C_{p,\mathrm{fus}} = 0$ vs. $\Delta C_{p,\mathrm{fus}} = \Delta S_{\mathrm{fus}}$.

Table S1: The List of Solvents Considered in This Study

Alias	Solvent	Classification[b]	CAS RN	SMILES
1BuOH	1-Butanol	(PP) Alcohol	71-36-3	OCCCC
1OctOH	1-Octanol	(PP) Alcohol	111-87-5	OCCCCCCCC
1PrOH	1-Propanol	(PP) Alcohol	71-23-8	OCCC
2BuOH	2-Butanol	(PP) Alcohol	78-92-2	CCC(C)O
2M1PrOH	2-Methyl-1-propanol	(PP) Alcohol	78-83-1	CC(C)CO
EtOH	Ethanol	(PP) Alcohol	64-17-5	CCO
GLY†	Glycerol	(PP) Alcohol	56-81-5	OCC(O)CO
iPrOH	Isopropyl Alcohol	(PP) Alcohol	67-63-0	CC(C)O
MeOH	Methanol	(PP) Alcohol	67-56-1	CO
THFA†	Tetrahydrofurfuryl alcohol	(PP) Alcohol	97-99-4	OCC1CCCO1
Furfural†	Furfural	(PA) Aldehyde	98-01-1	O=Cc1occc1
iOct	Isooctane	(NH) Alkane	540-84-1	CC(C)CC(C)(C)C
nHep	*n*-Heptane	(NH) Alkane	142-82-5	CCCCCCC
nHex	*n*-Hexane	(NH) Alkane	110-54-3	CCCCCC
nOct	*n*-Octane	(NH) Alkane	111-65-9	CCCCCCCC
DMF†	*N*,*N*-dimethylformamide	(PA) Amide	68-12-2	CN(C)C=O
NMP†	*N*-methyl-2-pyrrolidone	(PA) Amide	872-50-4	CN1CCCC1=O
UREA†	Urea	(PP) Amide	57-13-6	NC(=O)N
TCBz†	1,2,4-trichlorobenzene	(CH) Aromatic	120-82-1	c1cc(cc(c1Cl)Cl)Cl
Bz	Benzene	(NH) Aromatic	71-43-2	c1ccccc1
pCym†	*p*-Cymene	(NH) Aromatic	99-87-6	Cc1ccc(C(C)C)cc1
Tol	Toluene	(NH) Aromatic	108-88-3	Cc1ccccc1
AcOH†	Acetic acid	(PP) Carboxylic Acid	64-19-7	CC(=O)O
LacOH†	Lactic acid	(PP) Carboxylic Acid	50-21-5	CC(O)C(=O)O
LevOH†	Levulinic acid	(PP) Carboxylic Acid	123-76-2	CC(=O)CCC(=O)O
Chl	Chloroform	(CH) Chloroalkane	67-66-3	ClC(Cl)Cl
DCM	Dichloromethane	(CH) Chloroalkane	75-09-2	ClCCl
CyHx	Cyclohexane	(NH) Cycloalkane	110-82-7	C1CCCCC1
BuAc	Butyl acetate	(PA) Ester	123-86-4	CCCCOC(=O)C
DBM†	Dibutyl maleate	(PA) Ester	105-76-0	CCCCOC(=O)/C=C\C(=O)OCCCC
e-CL†	ϵ-Caprolactone	(PA) Ester	502-44-3	O=C1CCCCCO1
EtAc	Ethyl acetate	(PA) Ester	141-78-6	O=C(OCC)C
EtBu	Ethyl butyrate	(PA) Ester	105-54-4	CCCC(=O)OCC
EtFor	Ethyl formate	(PA) Ester	109-94-4	O=COCC
EtLac†	Ethyl lactate	(PP) Ester	97-64-3	CCOC(=O)C(C)O
EGDA†	Ethylene glycol diacetate	(PA) Ester	111-55-7	CC(=O)OCCOC(C)=O
GVL†	γ-Valerolactone	(PA) Ester	108-29-2	CC1CCC(=O)O1
iPrAc	Isopropyl acetate	(PA) Ester	108-21-4	CC(OC(C)C)=O
MeAc	Methyl acetate	(PA) Ester	79-20-9	O=C(OC)C
PrAc	Propyl acetate	(PA) Ester	109-60-4	O=C(OCCC)C
PC†	Propylene carbonate	(PA) Ester	108-32-7	CC1COC(=O)O1
GTA†	Triacetin	(PA) Ester	102-76-1	CC(=O)OCC(COC(C)=O)OC(C)=O
DIOX†	1,4-Dioxane	(PA) Ether	123-91-1	C1COCCO1
2-EE†	2-Ethoxyethanol	(PP) Ether	110-80-5	CCOCCO
2MeTHF†	2-Methyltetrahydrofuran	(PA) Ether	96-47-9	CC1CCCO1
DIPE†	Diisopropyl ether	(PA) Ether	108-20-3	CC(C)OC(C)C
DMI†	Dimethyl isosorbide	(PA) Ether	5306-85-4	CO[C@@H]1CO[C@H]2[C@@H]1OC[C@@H]2OC
THF†	Tetrahydrofuran	(PA) Ether	109-99-9	C1CCOC1
Ace	Acetone	(PA) Ketone	67-64-1	CC(C)=O
ACN	Acetonitrile	(PA) Nitrile	75-05-8	CC#N
DMSO†	Dimethyl sulfoxide	(PA) Sulfoxide	67-68-5	CS(=O)C
D-Lim†	D-Limonene	(NH) Terpene	5989-27-5	CC1=CC[C@@H](CC1)C(=C)C
H2O	Water	(PP) Water	7732-18-5	O

[a] The σ-profiles for all solvents taken from the UD database[S8] included in the GitHub repository of the benchmark open-source implementation of COSMO-SAC.[S9]
[b] (PP) polar protic; (PA) polar aprotic; (NH) nonpolar hydrocarbon; (CH) chlorinated hydrocarbon.
† Solvents included in the screening set in Section 3.3 of the main manuscript.

Table S2: Apelblat Equation (Eq 9 in the main manuscript) Constants (A, B, C) and Temperature Ranges for Selected Antioxidants.[d]

Antioxidant	Solvent	A	B	C	T range / K	Source
BHT	n-Heptane	56.750 85	−4955.779 69	−7.246 19	304–340	Our work, Chang *et al.*[S15]
	Ethanol	−1402.944 35	60 287.891 65	210.316 29	301–332	Our work, Chang *et al.*[S15]
	1-Octanol	−492.796 46	19 461.738 80	74.736 16	302–322	Our work, Chang *et al.*[S15]
	Benzene	−94.171 09	2629.665 25	14.819 69	291–339	Our work, Kosinski *et al.*[S16]
	Cyclohexane	−31.431 08	−465.011 23	5.613 56	297–340	Our work, Kosinski *et al.*[S16]
	Water	−35.71 [b]	9.35e−2[b]	–	293–313	Baluja *et al.*[S17]
	Methanol	−13.27 [b]	3.35e−2[b]	–	293–313	Baluja *et al.*[S17]
	1-Propanol	−10.30 [b]	2.76e−2[b]	–	293–313	Our work, Baluja *et al.*[S17]
	1-Butanol	−8.02 [b]	2.05e−2[b]	–	293–313	Baluja *et al.*[S17]
TPB	Acetone	−366.3861	12 834.95	55.586 95	278–323	Yang *et al.*[S18]
	Ethyl formate	−242.0296	7011.088	37.335 92	278–323	Yang *et al.*[S18]
	Methyl acetate	−190.9011	4682.594	29.746 01	278–323	Yang *et al.*[S18]
	Ethyl acetate	−59.979 14	−378.0810	9.912 154	278–323	Yang *et al.*[S18]
	Propyl acetate.	−200.9697	6279.508	30.821 05	278–323	Yang *et al.*[S18]
	Isopropyl acetate	−173.3980	5141.732	26.614 83	278–323	Yang *et al.*[S18]
	Butyl acetate	−182.6614	5842.117	27.881 48	278–323	Yang *et al.*[S18]
	Ethyl butyrate.	−173.2582	5394.623	26.533 34	278–323	Yang *et al.*[S18]
	n-Hexane	−112.1837	2642.538	17.436 98	278–323	Yang *et al.*[S18]
	n-Heptane.	−115.5091	2742.176	17.934 07	278–323	Yang *et al.*[S18]
	n-Octane	−140.8505	3788.642	21.738 80	278–323	Yang *et al.*[S18]
	Isooctane	−152.5721	4350.353	23.427 29	278–323	Yang *et al.*[S18]
NPH	Methanol	−267.26	4846.39	42.65	283–333	Wei *et al.*[S19]
	Ethanol	−279.30	6070.37	44.10	283–338	Wei *et al.*[S19]
	1-Propanol	−202.96	1071.19	33.52	283–343	Wei *et al.*[S19]
	2-Propanol	−193.95	692.60	32.17	283–343	Wei *et al.*[S19]
	1-Butanol	−350.35	7583.28	55.48	283–343	Wei *et al.*[S19]
	2-Butanol	−331.38	6906.48	52.43	283–343	Wei *et al.*[S19]
	2-Methyl-1-propanol	−439.28	12 473.70	67.95	283–343	Wei *et al.*[S19]
	n-Hexane	−528.310 98	15 137.556 41	82.270 68	283–338	Our work, Wei *et al.*[S20]
PPB	Water	−105.056 77	39.866 20	16.595 35	283–323	Ouyang *et al.*[S21]
	Ethanol	−97.206 15	1639.068 27	15.780 98	283–323	Ouyang *et al.*[S21]
	Methanol	−79.946 82	997.744 12	13.131 01	283–323	Ouyang *et al.*[S21]
	1-Propanol	−91.570 40	1345.814 83	14.964 07	283–323	Ouyang *et al.*[S21]
	Isopropanol	−124.763 99	2987.546 37	19.835 65	283–323	Ouyang *et al.*[S21]
	1-Butanol	−44.398 22	38.951 70	7.491 69	283–323	Ouyang *et al.*[S21]
	Acetone	−67.582 58	1734.285 08	10.621 34	283–323	Ouyang *et al.*[S21]
	Ethyl acetate	−70.331 00	1515.272 30	11.187 78	283–323	Ouyang *et al.*[S21]
	Methyl acetate	−67.112 94	907.037 43	10.948 83	283–323	Ouyang *et al.*[S21]
	Isobutanol	−87.362 23	1409.855 28	14.184 83	283–323	Ouyang *et al.*[S21]
	Butyl acetate	−66.158 00	1118.428 19	10.673 35	283–323	Ouyang *et al.*[S21]
	Acetonitrile	−200.746 24	5037.855 48	31.818 41	283–323	Ouyang *et al.*[S21]

[a] Data from industrial datasheets[S22–S24] are reported at a single temperature (293 K). For ODP, the datasheet was the only experimental solubility source; thus, no Apelblat parameters are reported.
[b] Linear Apelblat equation $\ln x_{\mathrm{a}}^{\mathrm{SLE,exp}} = A + BT$ as reported in Baluja *et al.*[S17]
[c] When *Our work* is indicated as a source, the Apelblat constants were regressed (by solving an overdetermined system of linear equations in the least squares sense) based on experimental data from the indicated reference.
[d] The number of significant figures for the Apelblat constants varies across sources and reflects the precision as reported in the original references (3-4 significant figures in Baluja *et al.*,[S17] 7 significant figures in Yang *et al.*,[S18] 2 decimal places in Wei *et al.*,[S19] and 5 significant figures in Ouyang *et al.*[S21]). Parameters regressed in this work are reported to 5 decimal places.

Table S3: Solubilities of the Selected Additives[a] Predicted by Two COSMO-SAC Configurations[b] at 293 K.

ID	Solvent	$x_a^{SLE,exp}$	Ref	$x_a^{SLE,classic}$	$x_a^{SLE,disp}$	$RErr_{\%}^{classic}$	$RErr_{\%}^{disp}$	$\ln \gamma_{classic}^{SLE}$	$\ln \gamma_{disp}^{SLE}$
BHT	nHep	0.268	S15	0.278	0.276	3.68	2.96	0.0989	0.106
	1OctOH	0.156	S15	0.394	0.393	152	151	−0.252	−0.249
	1BuOH	0.136	S17	0.367	0.358	170	164	−0.181	−0.155
	1PrOH	0.0985	S17	0.356	0.339	261	244	−0.149	−0.0996
	EtOH	0.0782	S15	0.338	0.302	332	286	−0.0979	0.0160
	MeOH	0.0275	S17	0.215	0.114	682	313	0.354	0.991
	H2O	1.22e−7	S15	7.47e−9	4.23e−9	93.9	96.5	17.5	18.1
ODP	Chl	0.229	S23	0.211	0.211	8.15	8.19	−1.10	−1.10
	Bz	0.163	S23	0.141	0.139	13.9	14.8	−0.696	−0.686
	Tol	0.148	S23	0.140	0.138	5.46	6.40	−0.691	−0.681
	CyHx	0.0956	S23	0.163	0.162	70.4	69.5	−0.843	−0.838
	EtAc	0.0923	S23	0.115	0.107	25.0	16.3	−0.499	−0.427
	nHex	0.0710	S23	0.146	0.145	106	105	−0.737	−0.731
	Ace	0.0250	S23	0.126	0.110	403	338	−0.586	−0.448
	EtOH	0.001 32	S23	0.0274	0.0222	1980	1590	0.939	1.15
	MeOH	0.000 364	S23	0.004 01	0.002 59	1000	611	2.86	3.30
	H2O	<3e−7	S23	2.54e−19	1.45e−19	n.d.	n.d.	40.2	40.7
NPH	Chl	0.199	S22	0.0937	0.0936	52.9	52.9	−4.15	−4.15
	DCM	0.109	S22	0.0868	0.0867	20.7	20.7	−4.07	−4.07
	EtAc	0.0622	S22	0.0649	0.0639	4.36	2.70	−3.78	−3.76
	Ace	0.0419	S22	0.0786	0.0761	87.6	81.6	−3.97	−3.94
	EtOH	0.0006	S22	0.0262	0.0248	4260	4030	−2.87	−2.82
	MeOH	0.000 250	S22	0.009 74	0.007 63	3800	2950	−1.88	−1.64
	nHex	0.000 220	S22	0.000 386	0.000 367	75.5	66.7	1.35	1.40
	nHex	9.08e−5	S20	0.000 386	0.000 367	325	304	1.35	1.40
	MeOH	0.000 215	S19	0.009 74	0.007 63	4440	3460	−1.88	−1.64
	EtOH	0.000 311	S19	0.0262	0.0248	8310	7850	−2.87	−2.82
	1PrOH	0.000 138	S19	0.0248	0.0241	17 900	17 400	−2.82	−2.79
	iPrOH	0.000 145	S19	0.0280	0.0273	19 200	18 700	−2.94	−2.91
	1BuOH	8.98e−5	S19	0.0208	0.0206	23 100	22 800	−2.64	−2.63
	2BuOH	4.62e−5	S19	0.0255	0.0252	55 100	54 500	−2.85	−2.83
	2M1PrOH	2.19e−5	S19	0.0213	0.0211	97 100	95 800	−2.67	−2.65
PPB	H2O	2.38e−5	S21	4.58e−5	3.41e−5	92.8	43.4	7.62	7.92
	EtOH	0.140	S21	0.205	0.201	46.4	43.6	−0.784	−0.765
	MeOH	0.142	S21	0.172	0.157	21.0	10.7	−0.611	−0.522
	1PrOH	0.139	S21	0.195	0.194	40.3	39.6	−0.736	−0.732
	iPrOH	0.151	S21	0.207	0.206	37.6	37.0	−0.797	−0.793
	1BuOH	0.181	S21	0.186	0.186	2.50	2.46	−0.689	−0.688
	Ace	0.264	S21	0.205	0.200	22.5	24.6	−0.786	−0.759
	EtAc	0.200	S21	0.142	0.141	29.2	29.8	−0.418	−0.409
	MeAc	0.162	S21	0.146	0.143	9.44	11.9	−0.450	−0.423
	2M1PrOH	0.139	S21	0.128	0.128	8.04	8.09	−0.314	−0.314
	BuAc	0.181	S21	0.101	0.101	44.2	44.2	−0.0778	−0.0778
	ACN	0.0602	S21	0.0618	0.0618	2.75	2.73	0.412	0.412

[a] Except TBP, which is incompatible with COSMO-SAC-dsp due to the presence of phosphorus atom.
[b] "Classic" refers to calculations using the COSMO-SAC-2010 model, while "disp" refers to calculations using the dispersion-corrected model.

Table S4: $\ln \gamma_i^\infty$ of the Selected Additives[a] in Various Solvents and Their Individual Terms at 293 K.

Additive	Solvent	$\ln \gamma_{\mathrm{comb}}^\infty$	$\ln \gamma_{\mathrm{resid}}^\infty$	$\ln \gamma_{\mathrm{disp}}^\infty$	$\ln \gamma_{\mathrm{COSMO-SAC-2010}}^\infty$	$\ln \gamma_{\mathrm{COSMO-SAC-dsp}}^\infty$
BHT	nHep	−0.16	0.54	0.01	0.37	0.39
	1OctOH	−0.07	−0.14	0.01	−0.21	−0.20
	1BuOH	−0.49	0.54	0.06	0.05	0.11
	1PrOH	−0.76	0.94	0.10	0.18	0.28
	EtOH	−1.21	1.64	0.18	0.43	0.62
	MeOH	−2.08	3.49	0.39	1.41	1.80
	H2O	−4.97	22.50	0.57	17.53	18.09
ODP	Chl	−3.59	−0.55	0.00	−4.14	−4.14
	Bz	−3.38	2.59	0.02	−0.79	−0.77
	Tol	−2.63	1.65	0.02	−0.98	−0.96
	CyHx	−2.78	1.35	0.01	−1.43	−1.42
	EtAc	−2.89	2.06	0.11	−0.83	−0.72
	nHex	−2.18	1.24	0.01	−0.94	−0.93
	Ace	−4.45	3.02	0.22	−1.42	−1.21
	EtOH	−5.56	6.73	0.18	1.17	1.34
	MeOH	−8.20	11.22	0.38	3.02	3.40
	H2O	−16.16	56.31	0.56	40.15	40.71
NPH	Chl	−9.07	−2.58	0.00	−11.65	−11.65
	DCM	−11.45	0.61	0.00	−10.84	−10.84
	EtAc	−7.48	0.01	0.06	−7.47	−7.42
	Ace	−10.77	0.60	0.14	−10.17	−10.03
	EtOH	−13.02	8.84	0.11	−4.17	−4.06
	MeOH	−18.37	16.31	0.28	−2.06	−1.78
	nHex	−6.01	7.63	0.04	1.63	1.67
	nHex	−6.01	7.63	0.04	1.63	1.67
	MeOH	−18.37	16.31	0.28	−2.06	−1.78
	EtOH	−13.02	8.84	0.11	−4.17	−4.06
	1PrOH	−9.99	6.12	0.05	−3.87	−3.82
	iPrOH	−10.21	6.03	0.05	−4.19	−4.14
	1BuOH	−7.98	4.63	0.02	−3.35	−3.33
	2BuOH	−8.26	4.45	0.02	−3.80	−3.78
	2M1PrOH	−8.39	4.98	0.02	−3.41	−3.39
PPB	H2O	−3.57	11.19	0.29	7.62	7.92
	EtOH	−0.76	−0.71	0.05	−1.48	−1.43
	MeOH	−1.41	0.28	0.17	−1.14	−0.97
	1PrOH	−0.43	−0.83	0.01	−1.26	−1.25
	iPrOH	−0.45	−0.96	0.01	−1.40	−1.39
	1BuOH	−0.24	−0.85	0.00	−1.09	−1.09
	Ace	−0.51	−1.16	0.07	−1.67	−1.61
	EtAc	−0.21	−0.38	0.01	−0.58	−0.57
	MeAc	−0.37	−0.35	0.05	−0.72	−0.68
	2M1PrOH	−0.25	−0.16	0.00	−0.41	−0.41
	BuAc	−0.04	0.02	0.00	−0.02	−0.02
	ACN	−0.89	1.26	0.00	0.38	0.38

[a] Except TBP, which is incompatible with COSMO-SAC-dsp due to the presence of phosphorus atom.

References

(S1) Mullins, E.; Liu, Y. A.; Ghaderi, A.; Fast, S. D. Sigma Profile Database for Predicting Solid Solubility in Pure and Mixed Solvent Mixtures for Organic Pharmacological Compounds with COSMO-Based Thermodynamic Methods. *Ind. Eng. Chem. Res.* **2008**, *47*, 1707–1725.

(S2) Klajmon, M. Purely Predicting the Pharmaceutical Solubility: What to Expect from PC-SAFT and COSMO-RS? *Mol. Pharmaceutics* **2022**, *19*, 4212–4232.

(S3) Antolovic, I.; Vrabec, J.; Klajmon, M. COSMOPharm: Drug-Polymer Compatibility of Pharmaceutical Amorphous Solid Dispersions from COSMO-SAC. *Mol. Pharmaceut.* **2024**, *21*, 4395–4415.

(S4) Klamt, A. COSMO-RS for Aqueous Solvation and Interfaces. *Fluid Phase Equilib.* **2016**, *407*, 152–158.

(S5) Groom, C. R.; Bruno, I. J.; Lightfoot, M. P.; Ward, S. C. The Cambridge Structural Database. *Acta Cryst. B* **2016**, *72*, 171–179.

(S6) Kim, S.; Chen, J.; Cheng, T. J.; Gindulyte, A.; He, J.; He, S. Q.; Li, Q. L.; Shoemaker, B. A.; Thiessen, P. A.; Yu, B.; Zaslavsky, L.; Zhang, J.; Bolton, E. E. PubChem 2019 Update: Improved Access to Chemical Data. *Nucleic Acids Res.* **2019**, *47*, D1102–D1109.

(S7) PubChem Database. National Center for Biotechnology Information, `https://pubchem.ncbi.nlm.nih.gov/`.

(S8) Xiong, R. C.; Sandler, S. I.; Burnett, R. I. An Improvement to COSMO-SAC for Predicting Thermodynamic Properties. *Ind. Eng. Chem. Res.* **2014**, *53*, 8265–8278.

(S9) Bell, I. H.; Mickoleit, E.; Hsieh, C. M.; Lin, S. T.; Vrabec, J.; Breitkopf, C.; Jager, A. A Benchmark Open-Source Implementation of COSMO-SAC. *J. Chem. Theory Comput.* **2020**, *16*, 2635–2646.

(S10) Neese, F. Software Update: The ORCA Program System—Version 6.0. *Wires Comput. Mol. Sci.* **2025**, *15*.

(S11) Hsieh, C. M.; Lin, S. T.; Vrabec, J. Considering the Dispersive Interactions in the COSMO-SAC Model for More Accurate Predictions of Fluid Phase Behavior. *Fluid Phase Equilib.* **2014**, *367*, 109–116.

(S12) Klamt, A. Conductor-Like Screening Model for Real Solvents: A New Approach to the Quantitative Calculation of Solvation Phenomena. *J. Phys. Chem.* **1995**, *99*, 2224–2235.

(S13) Klamt, A.; Jonas, V.; Bürger, T.; Lohrenz, J. C. W. Refinement and Parametrization of COSMO-RS. *J. Phys. Chem. A* **1998**, *102*, 5074–5085.

(S14) Klamt, A.; Eckert, F.; Hornig, M. COSMO-RS: A Novel View to Physiological Solvation and Partition Questions. *J. Comput. Aid. Mol. Des.* **2001**, *15*, 355–365.

(S15) Chang, S. S.; Maurey, J. R. Solubilities of BHT in Various Solvents. *J. Chem. Eng. Data* **1985**, *30*, 384–387.

(S16) Ksiazczak, A.; Kosinski, J. J. Vapor Pressure of Binary, Three-Phase (S-L-V) Systems and Solubility. *Fluid Phase Equilib.* **1988**, *44*, 211–236.

(S17) Baluja, S.; Bhesaniya, K.; Bhalodia, R.; Chanda, S. Solubility of Butylated Hydroxytoluene (BHT) in Aqueous and Alcohol Solutions from 293.15 to 313.15 K. *Int. Lett. Chem. Phys. Astron.* **2014**, *9*, 48–58.

(S18) Yang, J. Y.; Hou, B. H.; Huang, J. J.; Li, X.; Tian, B. Q.; Wang, N.; Bi, J. T.; Hao, H. X. Solution Thermodynamics of Tris-(2,4-ditert-butylphenyl)-phosphite in a Series of Pure Solvents. *J. Mol. Liq.* **2019**, *283*, 713–724.

(S19) Wei, D. W.; Chen, L. M. Solubility of Antioxidant 1010 in Pure Alkanols. *Fluid Phase Equilib.* **2009**, *277*, 9–14.

(S20) Wei, D. W.; Chen, L. M.; Xu, J. J.; Li, F. S. Measurement and Correlation of Solid-Liquid Equilibria of Irganox 1010 with n-Hexane. *Fluid Phase Equilib.* **2009**, *287*, 39–42.

(S21) Ouyang, J. B.; Chen, J.; Zhou, L. M.; Liu, Z. R.; Zhang, C. T. Solubility Measurement, Modeling, and Dissolution Thermodynamics of Propylparaben in 12 Pure Solvents. *J Chem Eng Data* **2020**, *65*, 4725–4734.

(S22) BASF Technical Datasheet: Irganox 1010. 2015; https://dispersions-resins-products.basf.us/products/irganox-1010, Accessed on June 30, 2025.

(S23) BASF Technical Datasheet: Irganox 1076. 2019; https://dispersions-resins-products.basf.us/products/irganox-1076, Accessed on June 30, 2025.

(S24) BASF Technical Datasheet: Irgafos 168. 2021; https://plastics-rubber.basf.com/northamerica/en/plastic_additives/products/antioxidants/irgafos/168, Accessed on June 30, 2025.
