# Supplementary figures and images for: COSMOSol: efficient solvent screening for polymer additives with open-source COSMO-SAC
Source: RSC Adv. 2026 Jul 21. Online ahead of print. doi: 10.1039/d6ra05129d (PMC13386427; doi:10.1039/d6ra05129d)

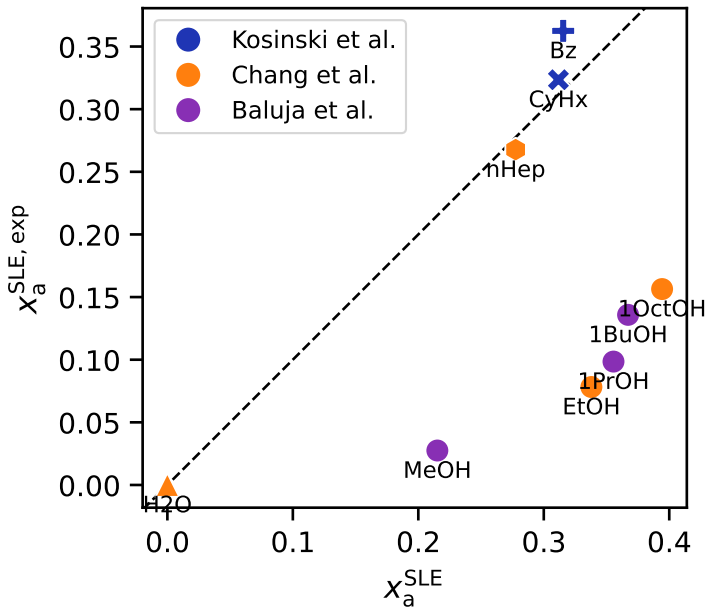

Supplement: RA-OLF-D6RA05129D-s001 [file RA-OLF-D6RA05129D-s001.zip › COSMOSol-main/results/manuscript_solubility_validation/x_SLE_vs_x_exp_BHT.pdf]

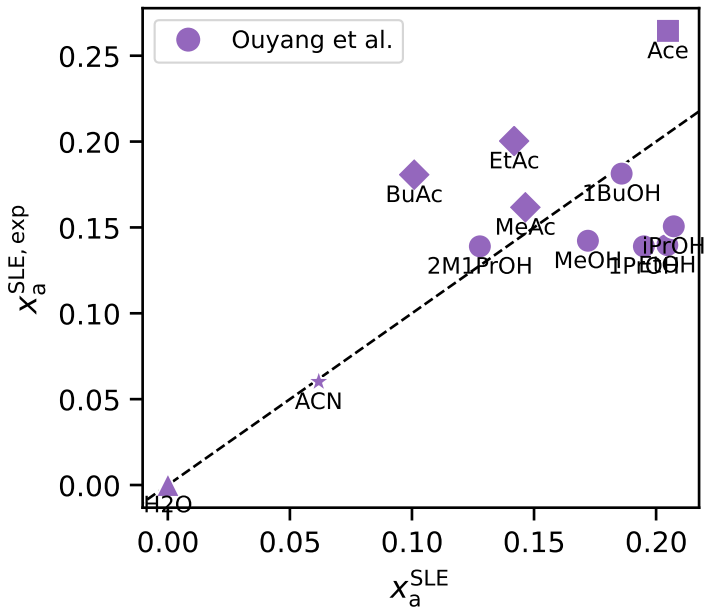

Supplement: RA-OLF-D6RA05129D-s001 [file RA-OLF-D6RA05129D-s001.zip › COSMOSol-main/results/manuscript_solubility_validation/x_SLE_vs_x_exp_PPB.pdf]

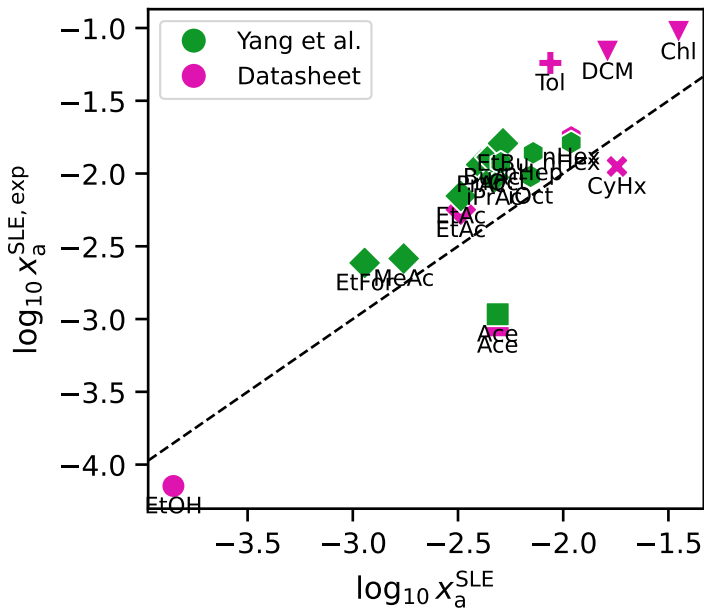

Supplement: RA-OLF-D6RA05129D-s001 [file RA-OLF-D6RA05129D-s001.zip › COSMOSol-main/results/manuscript_solubility_validation/x_SLE_vs_x_exp_TBP_log.pdf]

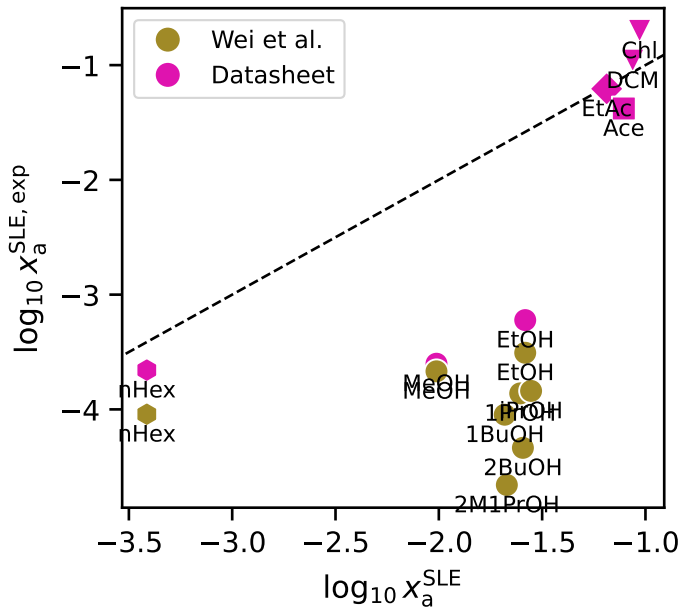

Supplement: RA-OLF-D6RA05129D-s001 [file RA-OLF-D6RA05129D-s001.zip › COSMOSol-main/results/manuscript_solubility_validation/x_SLE_vs_x_exp_NPH_log.pdf]

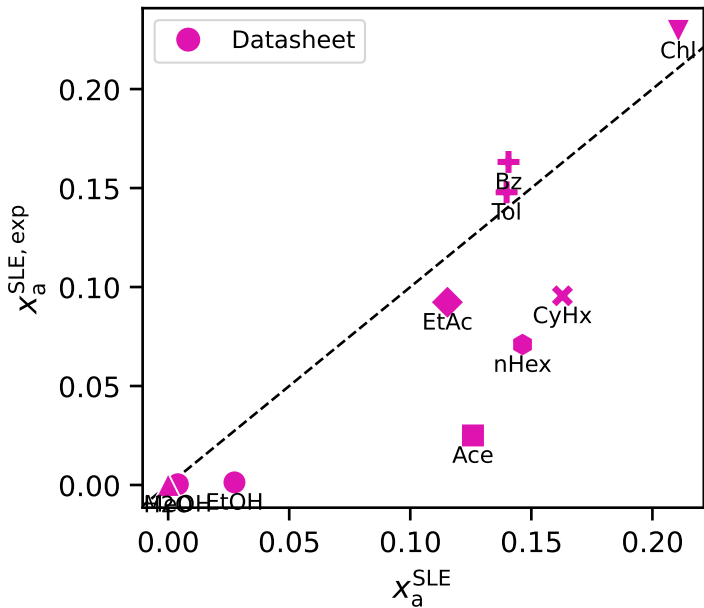

Supplement: RA-OLF-D6RA05129D-s001 [file RA-OLF-D6RA05129D-s001.zip › COSMOSol-main/results/manuscript_solubility_validation/x_SLE_vs_x_exp_ODP.pdf]

COSMO-SAC

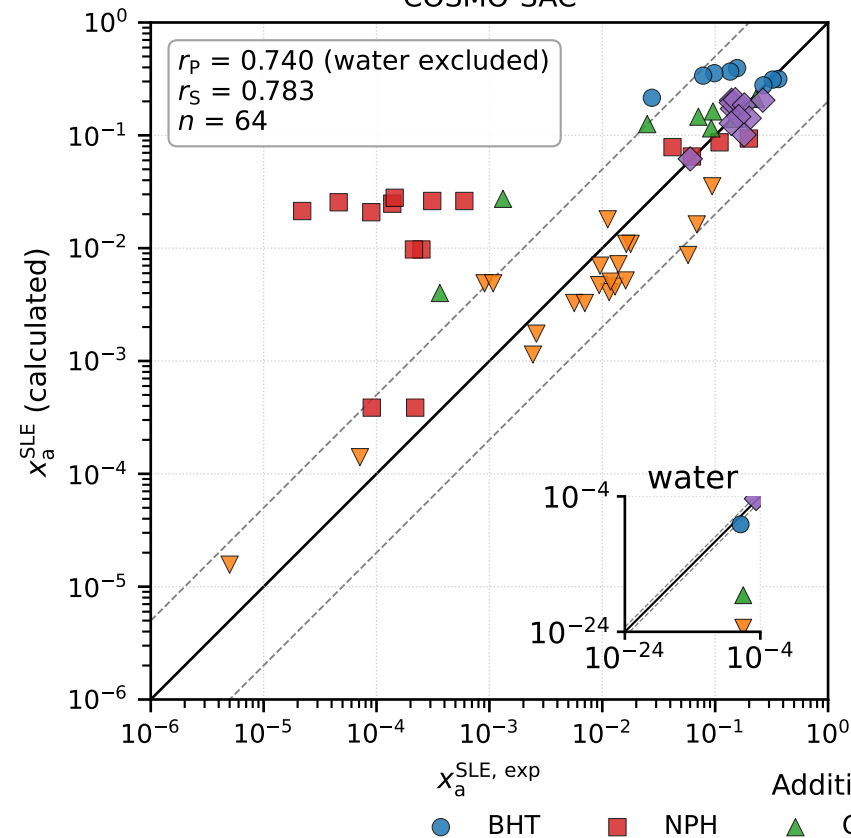

HANNA

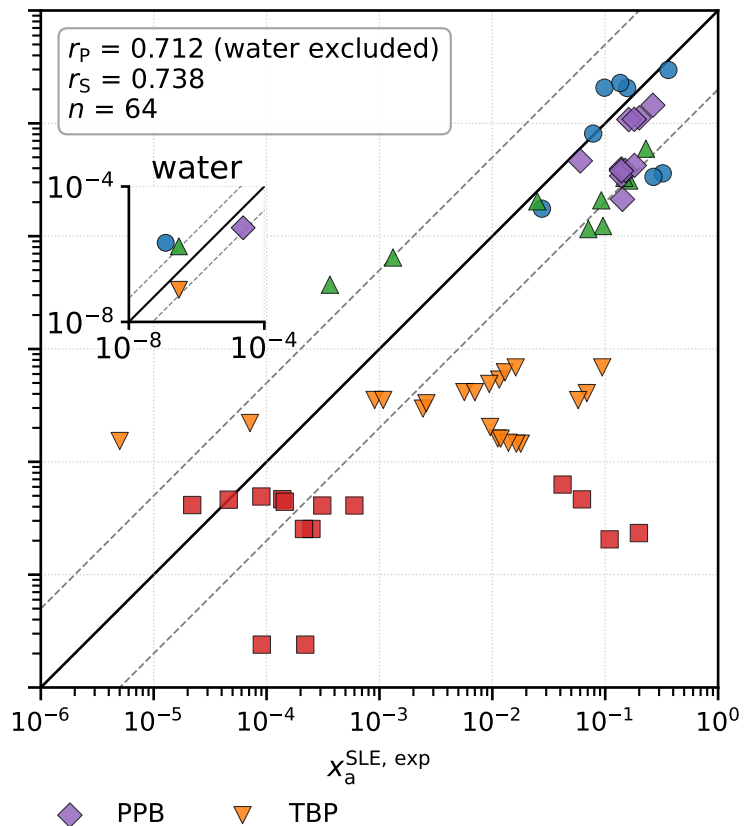

Supplement: RA-OLF-D6RA05129D-s001 [file RA-OLF-D6RA05129D-s001.zip › COSMOSol-main/results/manuscript_solubility_validation/parity_cosmo_vs_hanna.pdf]

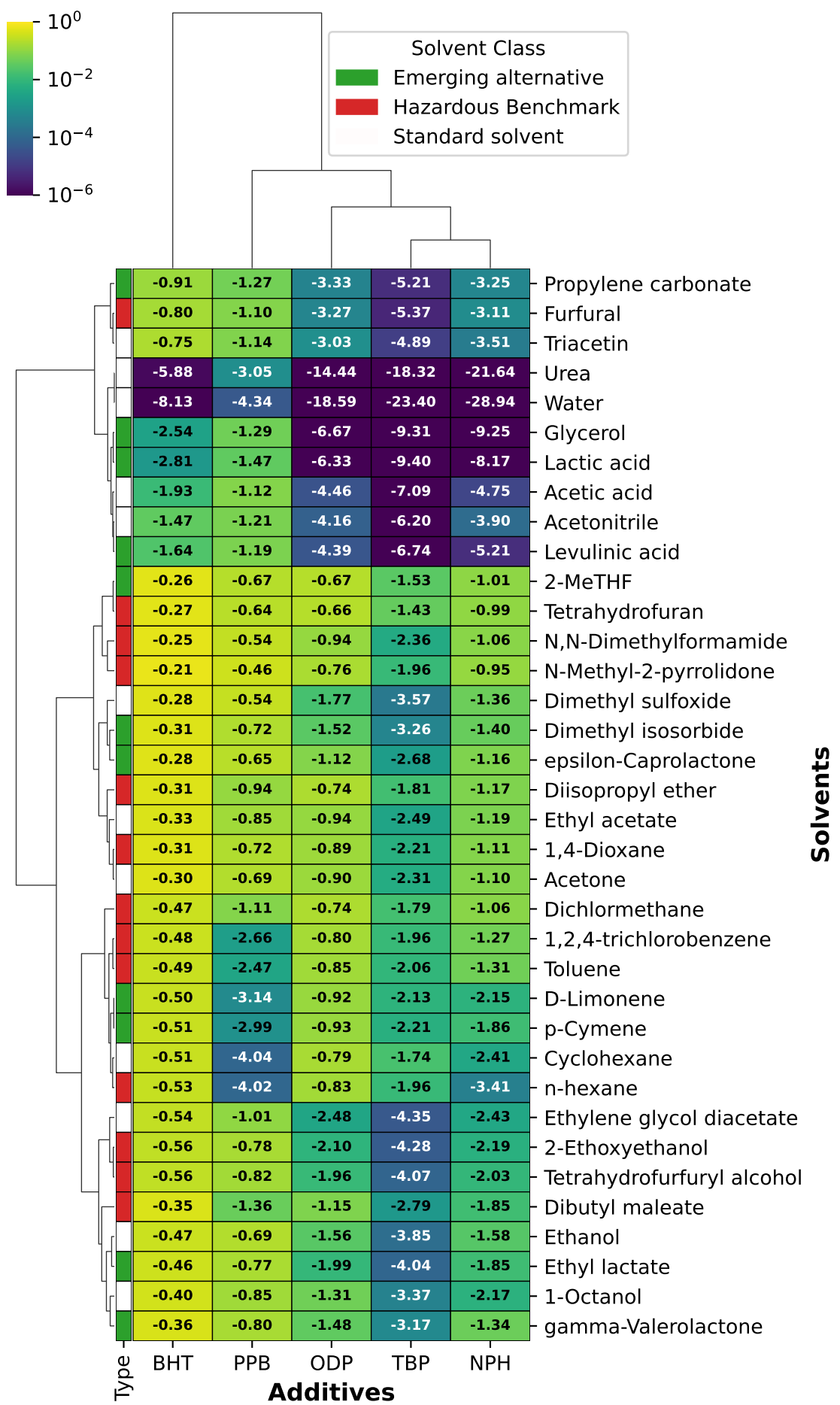

Supplement: RA-OLF-D6RA05129D-s001 [file RA-OLF-D6RA05129D-s001.zip › COSMOSol-main/results/manuscript_solvent_screening/solubility_screening_clustermap.pdf]

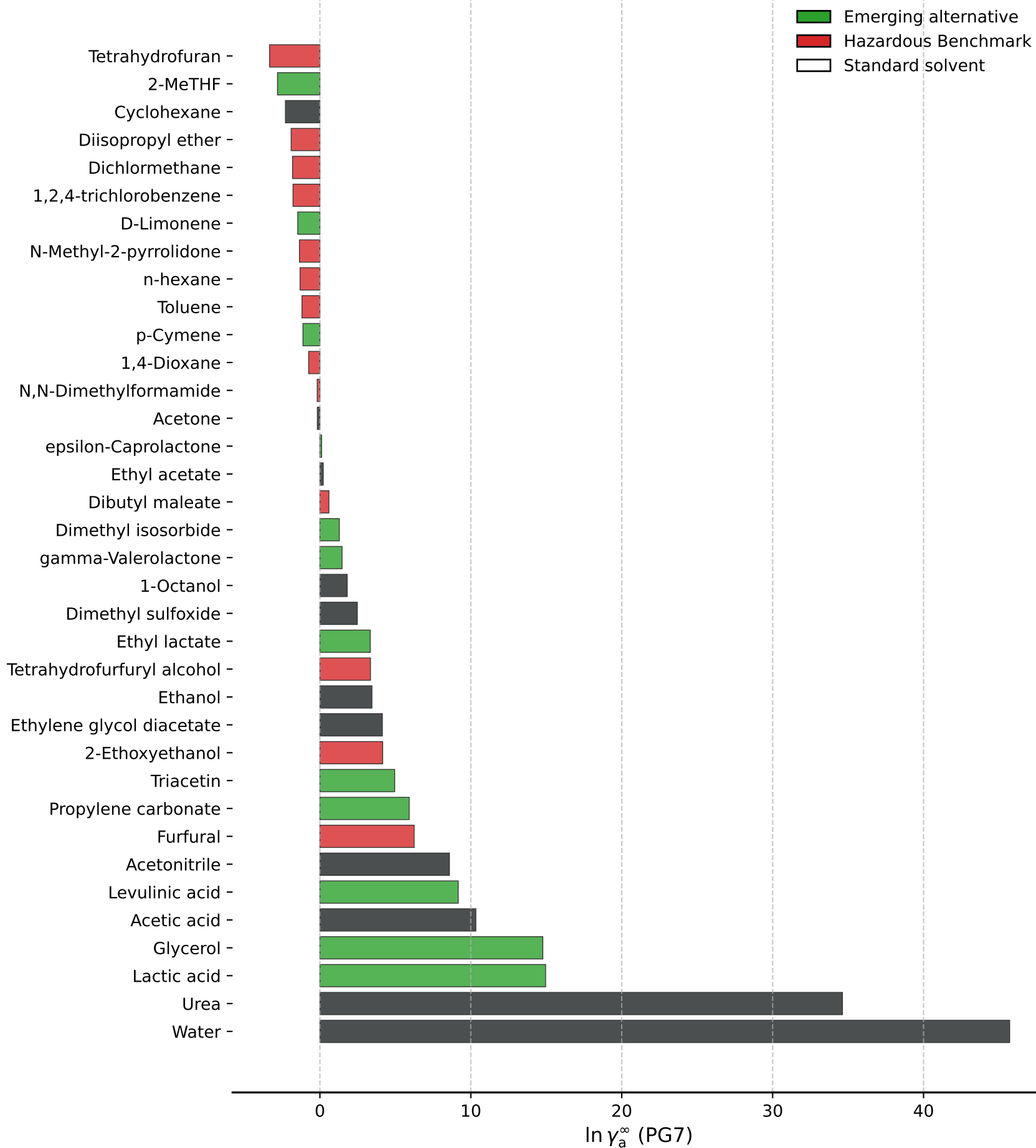

Supplement: RA-OLF-D6RA05129D-s001 [file RA-OLF-D6RA05129D-s001.zip › COSMOSol-main/results/manuscript_solvent_screening/PG7_screening_lngammainf.pdf]

NHB

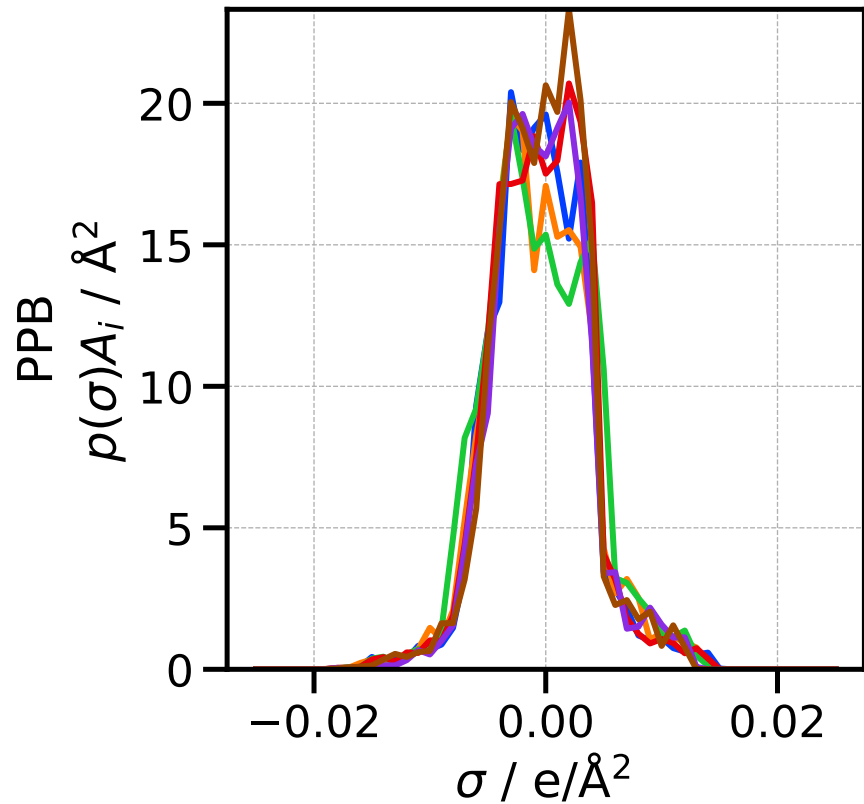

OH

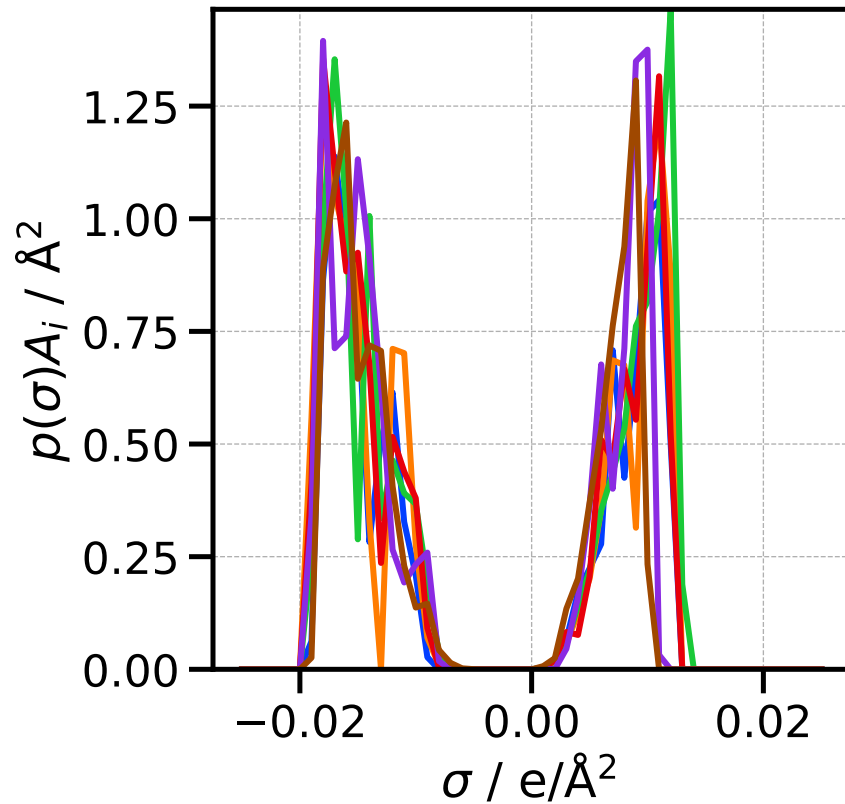

OT

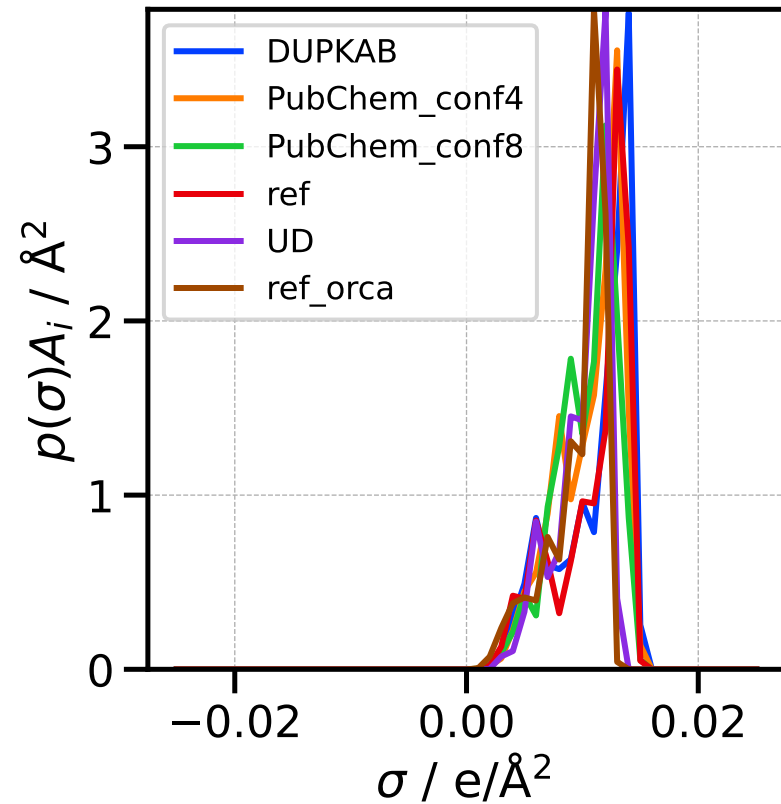

Supplement: RA-OLF-D6RA05129D-s001 [file RA-OLF-D6RA05129D-s001.zip › COSMOSol-main/parameters/sigma_profiles/additives/PPB_sensitivity/PPB_sigma_profiles_all.pdf]
